# Medical Therapies for Uterine Fibroids – A Systematic Review and Network Meta-Analysis of Randomised Controlled Trials

**DOI:** 10.1371/journal.pone.0149631

**Published:** 2016-02-26

**Authors:** Kurinchi S. Gurusamy, Jessica Vaughan, Ian S. Fraser, Lawrence M. J. Best, Toby Richards

**Affiliations:** 1 University College London, Division of Surgery & Interventional Science, 9th Floor, Royal Free Hospital, Pond Street, London, NW3 2QG, United Kingdom; 2 Sydney Centre for Reproductive Health Research, Family Planning New South Wales, Sydney, NSW 2131, Australia; 3 University of Sydney, Sydney, NSW 2006, Australia; University of Edinburgh, UNITED KINGDOM

## Abstract

**Background:**

Uterine fibroids are common, often symptomatic and a third of women need repeated time off work. Consequently 25% to 50% of women with fibroids receive surgical treatment, namely myomectomy or hysterectomy. Hysterectomy is the definitive treatment as fibroids are hormone dependent and frequently recurrent. Medical treatment aims to control symptoms in order to replace or delay surgery. This may improve the outcome of surgery and prevent recurrence.

**Purpose:**

To determine whether any medical treatment can be recommended in the treatment of women with fibroids about to undergo surgery and in those for whom surgery is not planned based on currently available evidence.

**Study Selection:**

Two authors independently identified randomised controlled trials (RCT) of all pharmacological treatments aimed at the treatment of fibroids from a list of references obtained by formal search of MEDLINE, EMBASE, Cochrane library, Science Citation Index, and ClinicalTrials.gov until December 2013.

**Data Extraction:**

Two authors independently extracted data from identified studies.

**Data Synthesis:**

A Bayesian network meta-analysis was performed following the National Institute for Health and Care Excellence—Decision Support Unit guidelines. Odds ratios, rate ratios, or mean differences with 95% credible intervals (CrI) were calculated.

**Results and Limitations:**

A total of 75 RCT met the inclusion criteria, 47 of which were included in the network meta-analysis. The overall quality of evidence was very low. The network meta-analysis showed differing results for different outcomes.

**Conclusions:**

There is currently insufficient evidence to recommend any medical treatment in the management of fibroids. Certain treatments have future promise however further, well designed RCTs are needed.

## Introduction

Uterine fibroids are benign tumours of the uterus known as leiomyomas. Malignant transformation is rare. The prevalence of uterine fibroids varies between 5% and 65% depending on age, ethnicity, geographical region and quality of imaging techniques [1–5]. They can occur as single or multiple focal fibroids or can be diffuse [5, 6]. The mechanism for development of uterine fibroids is poorly understood. Both genetic factors such as mutations and environmental factors such as obesity have been implicated in the development of fibroids [7].Additionally they can be estrogen and progesterone dependent [8]. Symptoms related to fibroids include bleeding irregularities such as heavy, prolonged or irregular periods which may result in iron deficiency, anaemia, subfertility and preterm birth [4, 9–11]. Enlargement of the tumor may cause a mass effect such as pressure on the urinary bladder depending on the anatomical location of the fibroids, or may experience chronic pelvic pain, and pain during sexual intercourse. The proportion of women with fibroids who are symptomatic varies with the size and location of the fibroids with at least 60% of women suffering from one or more symptoms [4, 10, 11]. Classification and sub-classification by fibroid position and size is important. Such factors have clinical and research implications. The FIGO PALM-COEIN classification listed eight types of leiomyoma however there is ongoing debate regarding interpretation [12]. Approximately 25% to 50% require treatment [5]. It is suggested that these symptoms and sequelae may decrease the health-related quality of life [13, 14], with 30% suffering symptoms severe enough to miss work [14].

Fibroid treatment includes medical and surgical management. In the USA, between 22 and 63% of women who seek medical help for symptoms related to uterine fibroids undergo surgical management while the remaining women undergo short-term medical treatment with hormonal agonists and antagonists [15]. Of the women who undergo surgical treatment, 84–94% undergo hysterectomy (mostly open or vaginal hysterectomies), 5–9% undergo myomectomy (removal of fibroids; mostly open), 1–4% undergo endometrial ablation (removal or destruction of the endometrium), and 1–3% undergo uterine artery embolization (obstruction of blood flow to uterine artery). The direct treatment costs (including the costs of medical treatments involved in surgery) in US have been estimated to be between US $6,000 and $12,000 for hysterectomy, between $7,000 and $15,000 for myomectomy, between US $7,000 and $13,000 for uterine artery embolization, US$5,000 for endometrial ablation, and between US $6,000 and $9,000 for non-surgical treatment [15, 16]. Thus, uterine fibroids cause a large socioeconomic burden.

Although hysterectomy is generally considered a safe operation, complications occur in a significant proportion of patients [15]. These include intra-operative bleeding (about 5% of people undergoing hysterectomy), post-operative fever (about 40% of people undergoing hysterectomy), post-operative surgical site infection (20%), deep vein thrombosis (symptomatic in <1%), vaginal cuff dehiscence (<1%), lower urinary tract injury (5%), gastrointestinal injury (<1%), and femoral or sciatic neuropathy (1% to 2%) [17]. There are alternative medical treatments. A variety of treatments have been used for uterine fibroids which take advantage of their hormonal dependence. These include gonadotropin releasing hormone (GnRH) analogues such as buserelin and, goserelin, selective estrogen receptor modulators (SERM) such as raloxifene, selective progesterone receptor modulators (SPRM) such as ulipristal, and progesterone antagonists such as mifepristone [18–21]. These drugs shrink the size of the fibroid and uterine volume [22] and hence have the potential to provide relief from symptoms in patients who undergo medical treatment. These drugs also provide symptomatic relief in patients waiting for surgery and enable vaginal or laparoscopic surgery, allowing a shorter hospital stay and quicker return to normal activities compared with open surgery [23]. Many of these drugs have a significant adverse event profile which limit the duration of administration. For example, GnRH analogues cause hypoestrogenism which leads to hot flushes and bone loss, limiting the treatment to a maximum of 3 to 6 months. These drugs are also associated with significant costs. While several meta-analyses have been performed comparing different medical treatments used in the management of fibroids [18–20], there has been no multiple treatment comparison meta-analysis or network meta-analysis. Such an analysis allows comparison of multiple treatments simultaneously and a Bayesian analysis allows ranking of treatments based on probability of being the best treatment [24]. The aim of this research is to determine whether any medical treatment is useful in the treatment of women with fibroids about to undergo surgery and in those for whom surgery is not planned.

## Methods

The systematic review was conducted following the PRISMA (Transparent Reporting of Systematic Reviews and Meta-analyses) reporting standards, those of the Cochrane collaboration, and the National Institute for Health Research and Clinical Excellence Decision Support Unit (NICE DSU) guidelines [25–27]. The detailed process is described in S1 Appendix. In short, randomised controlled trials which addressed one of the following comparisons were included.

1. Medical versus surgical treatments for fibroids.
2. Different medical treatments for fibroids (studies that compared different doses of the same drug were excluded unless the different drugs were compared with another drug or inactive control).
3. Different medical treatments prior to surgical treatment of fibroids (as before, studies that compared different doses of the same drug were excluded unless the different drugs were compared with another drug or inactive control).

The outcomes assessed for the first comparison included proportion requiring hysterectomy, quality of life, successful pregnancies, and costs.

The outcomes assessed for the second and third comparisons included proportion requiring subsequent surgery, treatment related adverse events, quality of life, blood transfusion requirements (proportion transfused and amount transfused), haemoglobin levels, successful pregnancies, length of hospital stay, and costs. In the comparison between different medical treatments prior to surgical treatment, the outcomes assessed included mortality, proportion undergoing laparoscopic or vaginal hysterectomy and laparoscopic or hysteroscopic myomectomy as applicable, treatment related adverse events, quality of life, blood transfusion requirements, haemoglobin levels, successful pregnancies (in only those in reproductive age group undergoing myomectomy), and resource measures such as length of hospital stay, operating time, and overall costs. The Cochrane library, MEDLINE, EMBASE, Science Citation Index Expanded, and ClinicalTrials.gov were searched until December 2013. The search strategies are available in S1 Appendix. The references of the included trials were searched to identify further trials. Two authors (KG and JV), independently identified the trials for inclusion and extracted data related to the outcomes mentioned above and assessed the risk of bias (according to Cochrane tools) in the trials. All differences in opinion were resolved by discussion until consensus was reached.

The software Winbugs 1.4 was used to perform the network meta-analysis using a Bayesian framework. The models used for analysis were based on those available from NICE DSU. Binomial likelihood was used for binary outcomes such as proportion of people with successful pregnancies, poisson likelihood for count outcomes such as number of adverse events and for binary outcomes with too many zeros that did not allow the analysis by binomial likelihood, and normal likelihood for continuous outcomes to calculate the odds ratio, rate ratio, and mean difference (MD) with 95% credible intervals (CrI) respectively. We used normal distribution with large variance (10,000) (non-informative priors) for treatment effects to ensure that the choice of prior does not influence the posterior probabilities [27]. Three different starting points (initial values) (three chains) were used and a burn-in of 30,000 iterations to ensure that the final results were not dependent on the starting point. A further 30,000 iterations were run to obtain the effect estimates. The probability of being the best treatment, the probability of being one of the best two treatments, best three treatments and so on [24] was calculated for each outcome to generate a cumulative ranking probability.

Exploration of publication bias and other reporting bias by funnel plot asymmetry and Egger's regression method of exploration of publication bias [28] was planned but not performed because there were less than 10 trials for comparing the same intervention and control.

Summary of findings tables providing the number of studies and participants included in the network meta-analysis, quality of the evidence based on GRADE methodology [29], the relative effect (odds ratio or rate ratio) or the mean difference for each pairwise comparison, and illustrative absolute effect for odds ratio or rate ratio were created for each outcome based on the mean control group proportion or rate (for odds ratio and rate ratio respectively) and control group mean for mean difference and are available in S3 Appendix.

## Results

**Fig 1:**
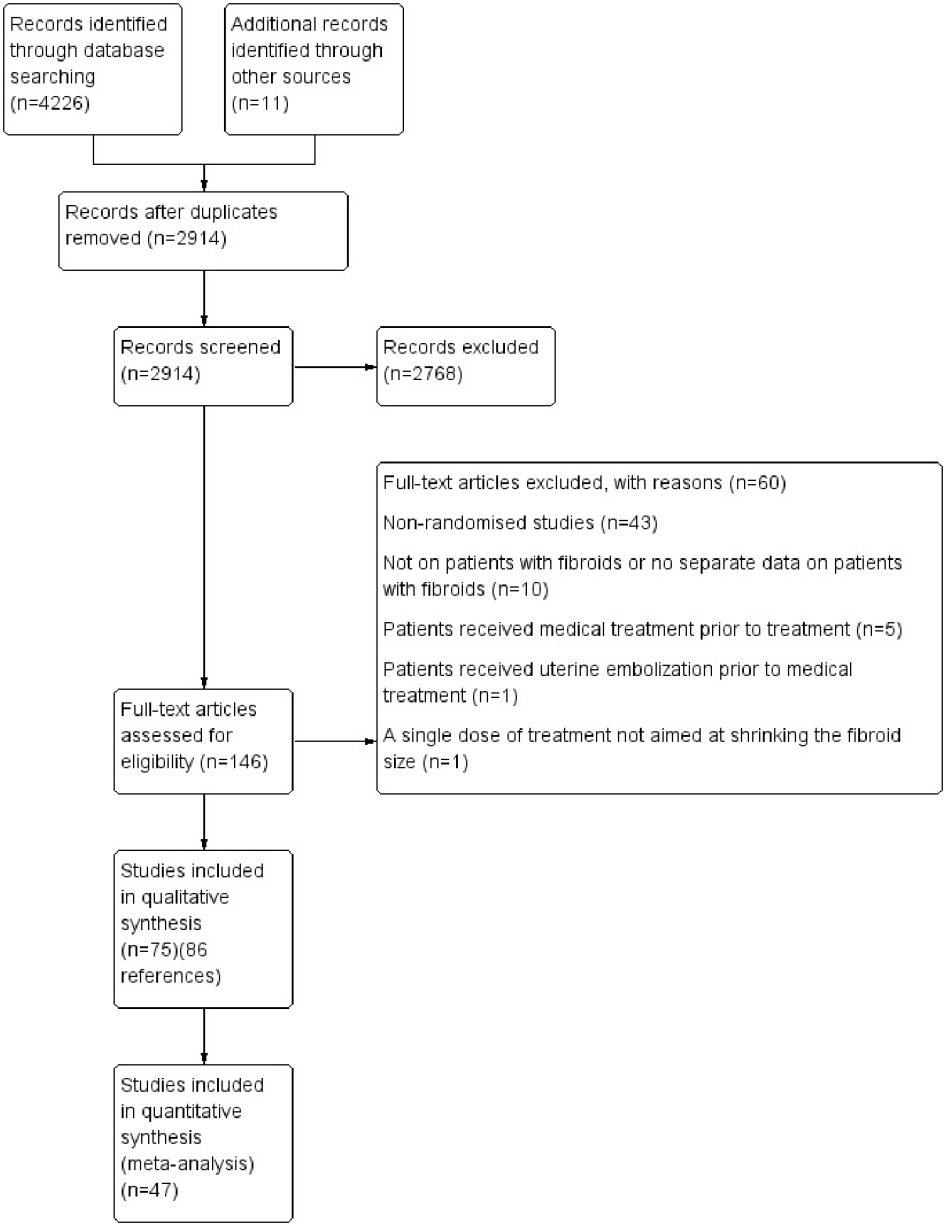
Reference flow diagram.

A total of 4237 references were identified by searching the electronic databases and the other sources. A total of 146 full texts were sought and 86 references [30–115] of 75 randomised controlled trials were included for this systematic review. Forty seven trials contributed to the network meta-analysis. The reference flow is shown in Fig 1. The characteristics of included studies are given in tables 1 and 11 in S3 Appendix for medical treatment and surgical treatment respectively.

Most trials included premenopausal women with symptomatic fibroids. The size of the fibroids varied between the studies. The details of size of the fibroids, menstrual status of the women, and the type of surgery, if any, that the women underwent are available in S2 Appendix and S3 Appendix.

### Effect estimates

Results are summarised in Table 1. The detailed results are available in S2 Appendix and S3 Appendix. There was no evidence of inconsistency for any of the analyses.

**Table 1:** Summary of results.

Outcome	Statistically significant comparisons	Effect estimate of statistically significant comparisons (95% CrI) (non-significant comparisons are not shown)
**Medical versus surgical treatment**		
Proportion undergoing hysterectomy	Medical treatment versus routine hysterectomy	RR 0.41; 95% CrI 0.29 to 0.57
**Medical treatment in women not scheduled to undergo surgery**^a^		
Proportion undergoing surgery	Tibolone/leuprolide versus placebo	OR 0.08; 95% CrI 0.01 to 0.47
Proportion with adverse events	No statistically significant differences between any of the pairwise comparisons	Not applicable
Number of adverse events	Leuprolide versus placebo	OR 5.57; 95% CrI 3.63 to 8.57
	Mifepristone versus placebo	OR 1.51; 95% CrI 1.09 to 2.08
	Medroxyprogesterone/leuprolide versus placebo	OR 3.33; 95% CrI 1.1 to 10.03
	Raloxiphene/leuprolide versus placebo	OR 3.78; 95% CrI 1.07 to 13.41
	Leuprolide versus asoprisnil	OR 3.87; 95% CrI 2.16 to 6.92
	Mifepristone versus leuprolide	OR 0.27; 95% CrI 0.16 to 0.46
Haemoglobin	Leuprolide versus placebo	MD 0.77; 95% CrI 0.37 to 1.17
	Mifepristone versus placebo	MD 1.88; 95% CrI 1.06 to 2.69
	Raloxiphene/leuprolide versus placebo	MD 0.97; 95% CrI 0.23 to 1.70
	Ulipristal versus placebo	MD 0.96; 95% CrI 0.61 to 1.31
	Mifepristone versus Leuprolide	MD 1.11; 95% CrI 0.2 to 2.02
**Medical treatment prior to planned surgery**^b^		
Proportion with adverse events	Goserelin versus placebo	OR 6.35; 95% CrI 3.33 to 12.10
Number with adverse events	Goserelin versus no active treatment	OR 1.66; 95% CrI 1.33 to 2.06
	Leuprolide versus no active treatment	OR 1.38; 95% CrI 1.17 to 1.62
Proportion undergoing abdominal hysterectomy	Leuprolide versus no active treatment	OR 0.55; 95% CrI 0.4 to 0.75
Proportion undergoing blood transfusion	Goserelin versus no active treatment	OR 0.40; 95% CrI 0.22 to 0.75
	Leuprolide versus no active treatment	OR 0.38; 95% CrI 0.2 to 0.71
Hospital stay	No statistically significant differences between any of the pairwise comparisons	Not applicable
Operating time	Leuprolide versus no active treatment	MD -8.56; 95% CrI -15.28 to -1.84
Haemoglobin	Leuprolide versus no active treatment	MD 1.28; 95% CrI 0.93 to 1.63
	Mifepristone versus no active treatment	MD 1.10; 95% CrI 0.04 to 2.15
	Tibolone/leuprolide versus no active treatment	MD 1.16; 95% CrI 0.65 to 1.66

OR = odds ratio. RaR = rate ratio. MD = mean difference. CrI = credible interval

^a^ Proportion of people who underwent blood transfusion or amount of blood transfused, proportion of people with successful pregnancies, length of hospital stays, and costs were not reported in any of the trials. Meta-analysis of quality of life outcomes was not performed because of incompatibility of reporting methods. A narrative summary can be found in S2 Appendix.

^b^ Quality of life, amount of blood transfused, cost of treatment, proportion with a successful pregnancy and proportion undergoing abdominal myomectomy did not provide data for meta-analysis

#### Medical versus surgical treatment

**Fig 2:**
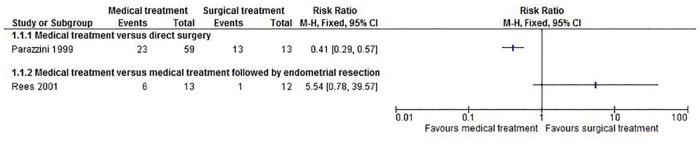
Medical treatment versus surgical treatments for uterine fibroids—proportion undergoing abdominal hysterectomy—forest plot.

Two trials compared medical versus surgical treatment [64] [77]. In one trial, the control group received surgery immediately [64] and in another trial, the control group underwent medical treatment and endometrial resection routinely [77]. The randomised participants were followed up for 3 years in the first trial [64] which included 72 premenopausal women with > 10 cm fibroids randomised to medical and surgical treatment and for 1 year in the second trial which included 25 premenopausal women with symptomatic fibroids with uterine size between 12 weeks and 16 weeks gestation randomised to medical and surgical treatment [77]. Both trials were at unclear or high risk of bias in most domains. The risk ratio of undergoing hysterectomy at 3 years in the first trial [64] was statistically significantly lower in the medical treatment group than direct surgery group (RR 0.41; 95% CrI 0.29 to 0.57; P < 0.00001). The proportion of people who underwent hysterectomy at 12 months was not statistically significant between the medical treatment and medical treatment followed by endometrial resection groups in the second trial (RR 5.54; 95% CrI 0.78 to 39.57; P = 0.09) [77]. However, neither of these trials reported any other outcome and so we were unable to determine whether there was any significant difference in mortality, number of successful pregnancies or the overall quality of life between medical and surgical treatment. These results are shown in Fig 2.

#### Medical treatment in women not scheduled to undergo surgery

Outcomes investigated were: proportion requiring surgery; treatment related adverse events; quality of life; blood transfusion requirements (proportion and amount transfused); haemoglobin levels; successful pregnancies; length of hospital stay and costs.)

**Fig 3:**
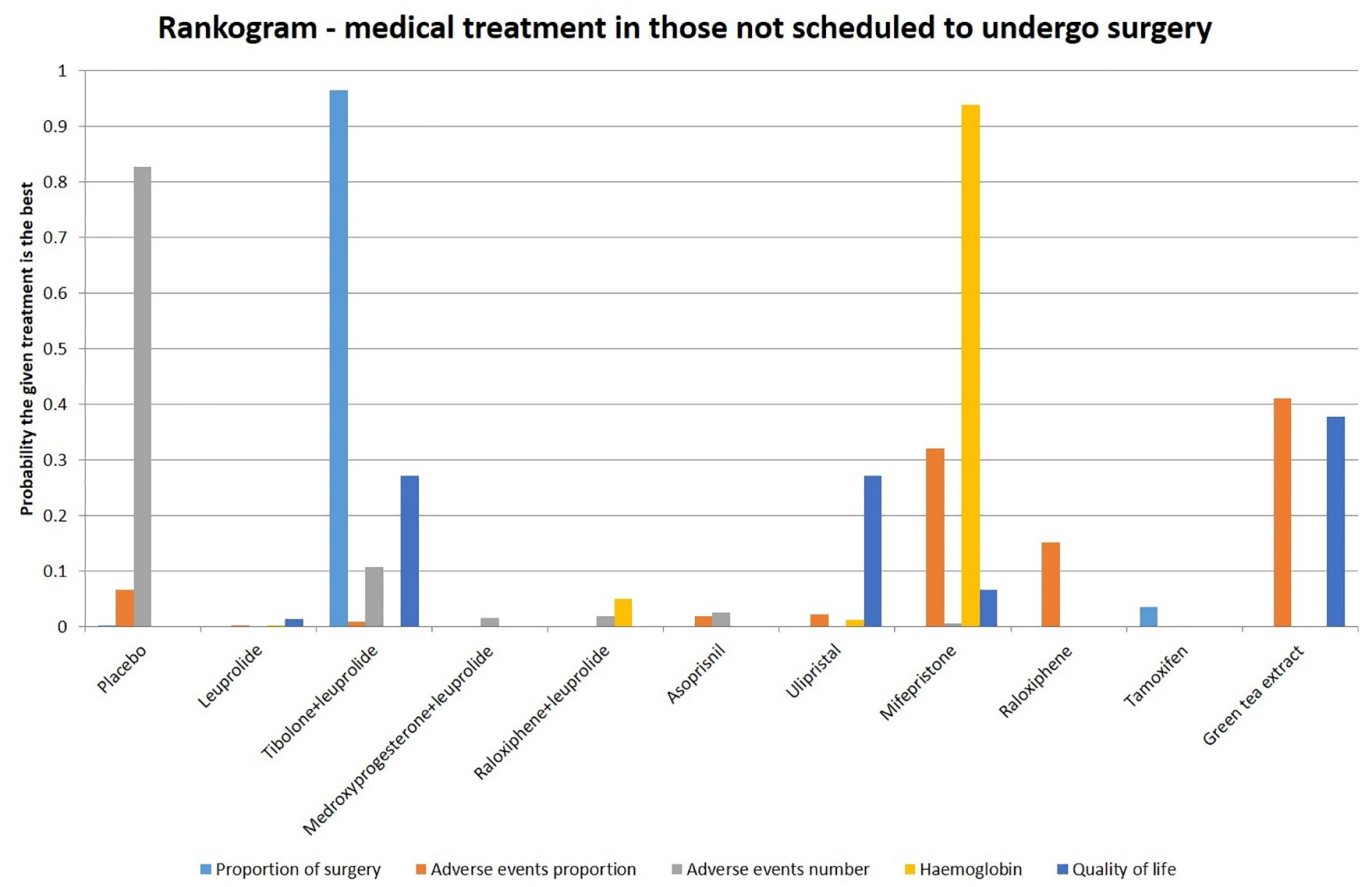
Different medical treatments in those not scheduled to undergo surgery—rankogram.

The proportion of women requiring surgery in the short-term was statistically significantly lower in the tibolone/leuprolide group compared to no treatment (OR 0.08; 95% CrI 0.01 to 0.47). There were no statistically significant differences in the proportion of people with adverse events between any of the treatments and the placebo. Leuprolide (OR 5.57; 95% CrI 3.63 to 8.57), mifepristone (OR 1.51; 95% CrI 1.09 to 2.08), medroxyprogesterone/leuprolide (OR 3.33; 95% CrI 1.1 to 10.03) and raloxiphene/leuprolide (OR 3.78; 95% CrI 1.07 to 13.41) had statistically significant more adverse events than the placebo group. Leuprolide had statistically significantly more adverse events than asoprisnil (OR 3.87; 95% CrI 2.16 to 6.92) and mifepristone had significantly fewer adverse events than leuprolide (OR 0.27; 95% CrI 0.16 to 0.46). All six studies reporting quality of life as an outcome found an increased quality of life scores with medical treatments, because of incompatibility of different reporting methos used meta-analysis is not appropriate. A more detailed narrative summary can be found in S2 App. Four treatments were found to statistically significantly increase haemoglobin levels compared to a group receiving placebo. These included: leuprolide (MD 0.77; 95% CrI 0.37 to 1.17), mifepristone (MD 1.88; 95% CrI 1.06 to 2.69), raloxiphene/leuprolide (MD 0.97; 95% CrI 0.23 to 1.7) and ulipristal (MD 0.96; 95% CrI 0.61 to 1.31). Mifepristone was found to cause significantly higher haemoglobin levels than leuprolide (MD 1.11; 95% CrI 0.20 to 2.02). These results are summarised in Fig 3. More detailed results are described in S2–S17 Figs, including forest plots and cumulative probability rankings.

The effect estimates of each pairwise comparison are available in tables 3–10 in S3 Appendix. The proportion of people with successful pregnancies, proportion of people who underwent blood transfusion or amount of blood transfused, length of hospital days, and costs were not reported in any of the trials.

#### Medical treatment in women prior to planned surgery

Outcomes investigated were: proportion undergoing abdominal myomectomy; proportion undergoing abdominal hysterectomy; treatment related adverse events; quality of life; blood transfusion requirements (proportion and amount transfused); haemoglobin levels; successful pregnancies; length of hospital stay; operating time and costs

**Fig 4:**
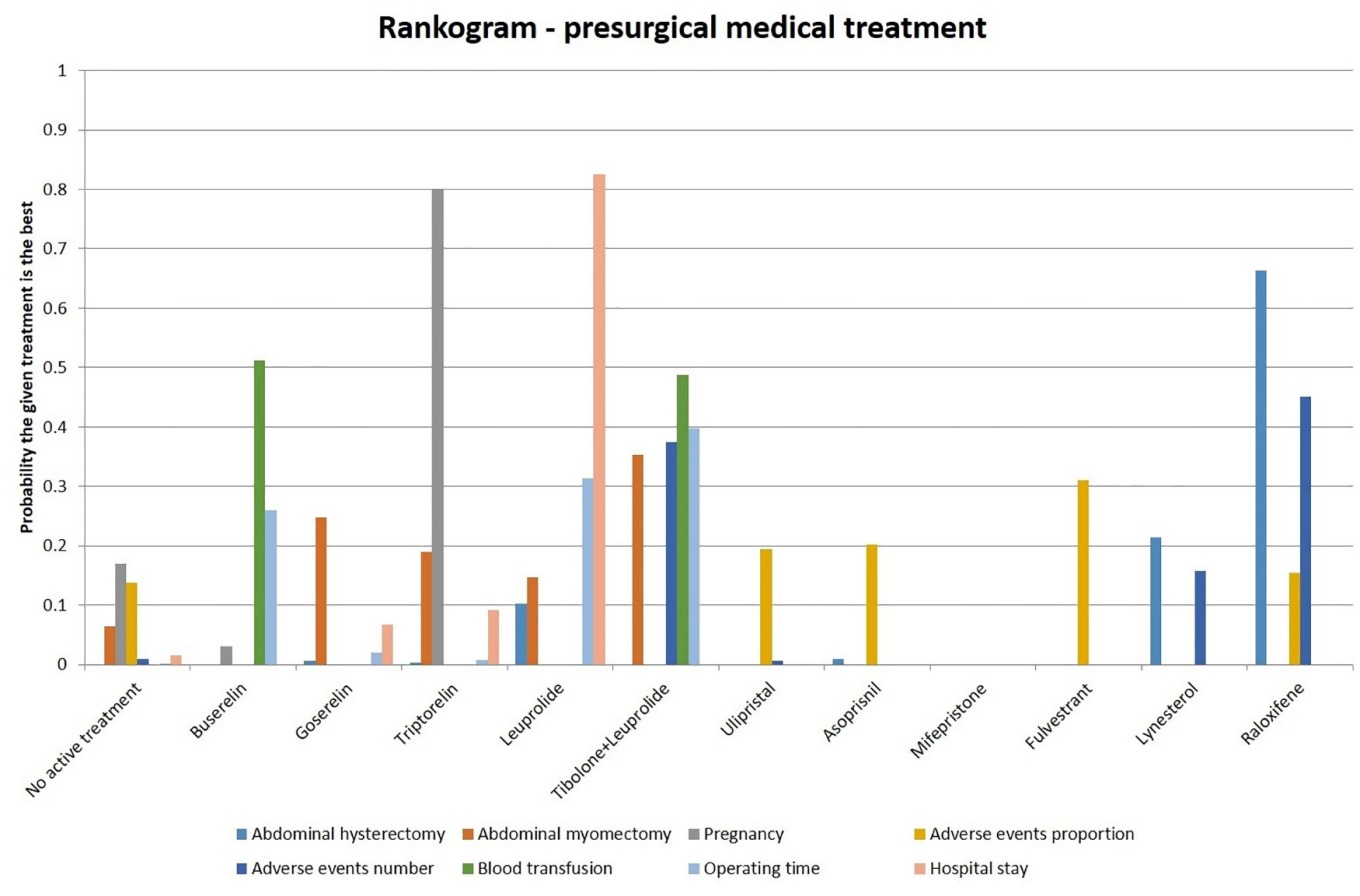
Different presurgical medical treatments—rankogram.

The proportion of women with adverse events was statistically significantly higher in the goserelin group compared to those receiving placebo (OR 6.35; 95% CrI 3.33 to 12.10). Goserelin also caused a statistically significant increase in the number of women with adverse events compared to the group with no active treatment (OR 1.66; 95% CrI 1.33 to 2.06), as did leuprolide (OR 1.38; 95% CrI 1.17 to 1.62). Leuprolide did significantly reduce the proportion of women undergoing abdominal hysterectomy in the short-term compared to women receiving no active treatment (OR 0.55; 95% CrI 0.40 to 0.75). Both goserelin (OR 0.40; 95% CrI 0.22 to 0.75) and leuprolide (OR 0.38; 95% CrI 0.20 to 0.71) significantly reduced the proportion of women undergoing blood transfusion. There were no statistically significant differences in hospital stay between any of the treatments and no active treatment. Leuprolide reduced the operating time compared to the group receiving no active treatment (MD -8.56; 95% CrI -15.28 to -1.84). Three treatments were found to statistically increase haemoglobin levels compared to the group receiving no active treatment. These were: leuprolide (MD 1.28; 95% CrI 0.93 to 1.63), mifepristone (MD 1.10; 95% CrI 0.04 to 2.15) and tibolone/leuprolide (MD 1.16; 95% CrI 0.65 to 1.66). These results are summarised in Fig 4. More detailed results are described in S18–S45 Figs, including forest plots and cumulative probability rankings.

The effect estimates of each pairwise comparison are available in tables 13–26 in S3 Appendix. Quality of life, amount of blood transfused, cost of treatment, proportion with a successful pregnancy and proportion undergoing abdominal myomectomy did not have available data for meta-analysis.

## Discussion

This is the first network meta-analysis assessing the effect of different pharmacological interventions in the treatment of uterine fibroids.

### Medical versus surgical treatment

Only two trials compared medical with surgical treatment. Network meta-analysis could not be performed because of differences in the treatments in the two trials. The trials were at unclear or high risk of bias for most domains and the overall quality of evidence was poor. The only outcome of interest reported was the proportion that underwent hysterectomy. The two trials reported different results and so there is currently no evidence to recommend medical treatment as a substitute for surgical treatment.

### Medical treatment in women not scheduled to undergo surgery

The network meta-analyses showed different results for different outcomes. In the ranking of treatments for different outcomes, more than 80% probability of one treatment being the best treatment was noted only in the proportion undergoing surgery in which Tibolone/leuprolide combination was the best treatment, the number of adverse events for which placebo was the best treatment, and haemoglobin for which mifepristone was the best treatment. The number of adverse events included disease-related and treatment-related adverse events. The severity of the adverse events was not reported but most of the adverse events reported appeared to be mild. In addition, there is currently no evidence to suggest that patients consider one type of adverse event to be worse than another type. Until the relative importance and the variability in the relative importance that patients assign to these adverse events is obtained in relation to the benefits seen in avoiding or postponing surgery, one cannot advocate medical treatment for the control of symptoms. This may be captured in quality of life instruments. In addition, the treatment can be given only for short periods of time because of the long-term risks, such as increased rate of bone loss for GnRH analogues, and it is not clear whether medical treatment only delays the inevitable at significant costs and adverse events without any major advantages. Approximately, 45% of patients who received no active treatment had surgery during the follow-up period. A minimum sample size of 932 participants will be required to detect a 20% reduction in the proportion of patients who require surgery with an alpha error of 0.05 and power of 0.8. The final haemoglobin level is higher for a number of interventions compared to no active treatment but the clinical significance of this difference in haemoglobin is not known since the trials did not report the blood transfusion requirements. Iron deficiency itself may be relevant for the treatment of fibroids with associated heavy menstrual bleeding and post-surgically where it may be slow to correct. Short-term quality of life appears to be improved for a number of treatments compared to placebo despite the increase in adverse events. However, long-term follow-up is necessary to determine whether this improvement in short-term quality of life and reduction in the proportion of people who undergo hysterectomy persists in the long run. The same reporting methodology must be used in future trials to allow for meta-analysis of results. Clinically long term assessment is crucial because fibroids are a long term condition. Short-term trials are of limited value to a gynaecologist deciding treatment for a given patient. A well designed randomised controlled trial with cost-effectiveness analysis is necessary before medical treatment can be routinely recommended for women with fibroids who are not scheduled to undergo hysterectomy.

### Medical treatment in women prior to planned surgery

The network meta-analyses again showed different results for different outcomes. In the ranking of treatments for different outcomes, more than 80% probability of one treatment being the best treatment was noted only in the length of hospital stay in which leuprolide was the best treatment. The trials were at high risk of bias and overall quality of evidence was low. A number of treatments had more adverse events than inactive treatment. As mentioned earlier, until the relative importance that patients ascribe to these adverse events is determined, one cannot advocate medical treatment for the control of symptoms during the waiting time. Although leuoprolide appears to show benefit in a number of outcomes such as reduction in the proportion of women requiring abdominal hysterectomy, blood transfusion requirement, and length of hospital stay, the trials were at high risk of bias and the overall quality of evidence was low. So, no medical treatment can be recommended currently during the waiting time for surgery. Approximately, 75% of patients who received no active treatment had abdominal hysterectomy. A minimum sample size of 304 participants will be required to detect a 20% reduction in the proportion of patients who require hysterectomy with an alpha error of 0.05 and power of 0.8. Again, a well-designed randomised controlled trial with cost-effectiveness analysis is necessary before medical treatment can be routinely recommended for women waiting for surgery for fibroids.

The major strengths of this review were that we followed the PRISMA guidance for reporting and included all randomized controlled trials on the topic without any restriction on the language of publication. We also selected studies and extracted data independently, which decreases the errors. We chose non-informative priors and used three different chains of initial values which decreases the risk of error due to the choice or prior and initial values. The major weaknesses of the review are the risk of bias in the included trials and the sparse data for many of the outcomes despite the number of trials included in this review.

This is the first network meta-analysis on this topic. Some previous head-to-head comparisons found insufficient evidence to support medical treatment for fibroids [18] [20]while one review advocated the use of pre-operative GnRH analogues [22]. The differences in the conclusion between our review and the review that advocated the use of medical treatment may be because of new trials conducted in this field in the last 15 years and the use of clinical outcomes rather than surrogate outcomes such as decrease in fibroid size.

There is currently no evidence to support the routine use of medical treatment in women with uterine fibroids. Several treatments appear promising but the efficacy of these drugs should be assessed in low risk of bias trials powered to measure differences in clinical outcomes. Ideally these would be both large scale and long term. Ultimately medical treatment may involve continued treatment over a period of time to prevent surgery.

## Supporting Information

S1 Appendix Methods. (DOCX)

S2 Appendix Detailed results. (DOCX)

S3 Appendix Tables. (DOCX)

S1 Fig Medical treatment versus surgical treatment for uterine fibroids-proportion undergoing abdominal hysterectomy-forest plot. (JPG)

S2 Fig Medical treatment in women not scheduled to undergo surgery-Proportion undergoing surgery-forest plot. (JPG)

S3 Fig Medical treatment in women not scheduled to undergo surgery-Proportion undergoing surgery-network plot. (JPG)

S4 Fig Medical treatment in women not scheduled to undergo surgery-Proportion undergoing surgery-probability of best treatment. (JPG)

S5 Fig Medical treatment in women not scheduled to undergo surgery-Proportion undergoing surgery-cumulative ranking probability. (JPG)

S6 Fig Medical treatment in women not scheduled to undergo surgery-Proportion with adverse events-forest plot. (JPG)

S7 Fig Medical treatment in women not scheduled to undergo surgery-Proportion with adverse events-network plot. (JPG)

S8 Fig Medical treatment in women not scheduled to undergo surgery-Proportion with adverse events-probability of best treatment. (JPG)

S9 Fig Medical treatment in women not scheduled to undergo surgery-Proportion with adverse events-cumulative ranking probability. (JPG)

S10 Fig Medical treatment in women not scheduled to undergo surgery-Number of adverse events-forest plot. (JPG)

S11 Fig Medical treatment in women not scheduled to undergo surgery-Number of adverse events-network plot. (JPG)

S12 Fig Medical treatment in women not scheduled to undergo surgery-Number of adverse events-probability of best treatment. (JPG)

S13 Fig Medical treatment in women not scheduled to undergo surgery-Number of adverse events-cumulative ranking probability. (JPG)

S14 Fig Medical treatment in women not scheduled to undergo surgery-Haemoglobin-forest plot. (JPG)

S15 Fig Medical treatment in women not scheduled to undergo surgery-Haemoglobin-network plot. (JPG)

S16 Fig Medical treatment in women not scheduled to undergo surgery-Haemoglobin-probability of best treatment. (JPG)

S17 Fig Medical treatment in women not scheduled to undergo surgery-Haemoglobin-cumulative ranking probability. (JPG)

S18 Fig Presurgical medical treatment-Proportion with adverse events-forest plot. (JPG)

S19 Fig Presurgical medical treatment-proportion with adverse events-network plot. (JPG)

S20 Fig Presurgical medical treatment-proportion with adverse events-probability of best treatment. (JPG)

S21 Fig Presurgical medical treatment-proportion with adverse events-cumulative ranking probability. (JPG)

S22 Fig Presurgical medical treatment-number with adverse events-forest plot. (JPG)

S23 Fig Presurgical medical treatment-number with adverse events-network plot. (JPG)

S24 Fig Presurgical medical treatment-number with adverse events-probability of best treatment. (JPG)

S25 Fig Presurgical medical treatment-number with adverse events-cumulative ranking probability. (JPG)

S26 Fig Presurgical medical treatment-proportion undergoing abdominal hysterectomy-forest plot. (JPG)

S27 Fig Presurgical medical treatment-proportion undergoing abdominal hysterectomy-network plot. (JPG)

S28 Fig Presurgical medical treatment-proportion undergoing abdominal hysterectomy-probability of best treatment. (JPG)

S29 Fig Presurgical medical treatment-proportion undergoing abdominal hysterectomy-cumulative ranking probability. (JPG)

S30 Fig Presurgical medical treatment-proportion undergoing blood transfusion-forest plot. (JPG)

S31 Fig Presurgical medical treatment-proportion undergoing blood transfusion-network plot. (JPG)

S32 Fig Presurgical medical treatment-proportion undergoing blood transfusion-probability of best treatment. (JPG)

S33 Fig Presurgical medical treatment-proportion undergoing blood transfusion-cumulative ranking probability. (JPG)

S34 Fig Presurgical medical treatment-hospital stay-forest plot. (JPG)

S35 Fig Presurgical medical treatment-hospital stay-network plot. (JPG)

S36 Fig Presurgical medical treatment-hospital stay-probability of best treatment. (JPG)

S37 Fig Presurgical medical treatment-hospital stay-cumulative ranking probability. (JPG)

S38 Fig Presurgical medical treatment-operating time-forest plot. (JPG)

S39 Fig Presurgical medical treatment-operating time-network plot. (JPG)

S40 Fig Presurgical medical treatment-operating time-probability of best treatment. (JPG)

S41 Fig Presurgical medical treatment-operating time-cumulative ranking probability. (JPG)

S42 Fig Presurgical medical treatment-haemoglobin-forest plot. (JPG)

S43 Fig Presurgical medical treatment-haemoglobin-network plot. (JPG)

S44 Fig Presurgical medical treatment-haemoglobin-probability of best treatment. (JPG)

S45 Fig Presurgical medical treatment-haemoglobin-cumulative ranking probability. (JPG)

S1 PRISMA Checklist Completed PRISMA Checklist for meta-analyses. (DOC)
