# Supplementary material for: Medical Therapies for Uterine Fibroids – A Systematic Review and Network Meta-Analysis of Randomised Controlled Trials
Source: PLoS One. 2016 Feb 26;11(2):e0149631. doi: 10.1371/journal.pone.0149631 (PMC4769153; doi:10.1371/journal.pone.0149631)
Supplement: S1 Appendix — (DOCX) [file pone.0149631.s001.docx]

# Appendix 1: Methods

## Types of studies

Randomised controlled trials irrespective of language, blinding, sample size, or publication status were included.

## Types of comparisons

All studies that compared different medical treatments to one another or to inactive control (placebo or no treatment) in women with fibroids, irrespective of size or symptoms, about to undergo surgery and in those who do not undergo surgery were included. Studies that compared different doses of the same drug were excluded unless the different drugs were compared with another drug or inactive control. The data from different doses of the same drug were pooled together for the meta-analysis. We performed three different comparisons.

1. Medical versus surgical treatments.
2. Different medical treatments.
3. Different medical treatments prior to surgical treatment.

## Outcomes

The outcomes assessed for the first comparison included proportion requiring hysterectomy, quality of life, successful pregnancies, and costs.

The outcomes assessed for the second comparison included proportion requiring surgery, treatment related adverse events, quality of life, blood transfusion requirements (proportion and amount transfused), haemoglobin levels, successful pregnancies, number of hospital days, and costs. In the comparison between different medical treatments prior to surgical treatment, the outcomes assessed included mortality, proportion undergoing laparoscopic or vaginal hysterectomy and laparoscopic or hysteroscopic myomectomy as applicable, treatment related adverse events, quality of life, blood transfusion requirements, haemoglobin levels, successful pregnancies (in only those in reproductive age group undergoing myomectomy), and resource measures such as number of hospital days, operating time, and overall costs.

## Study selection

The Cochrane library, MEDLINE, EMBASE, Science Citation Index Expanded, and ClinicalTrials.gov were searched until December 2013 using the following search strategies.

### Pubmed

((fibroid OR fibroids OR leiomyoma OR leiomyomas OR leiomyomata OR fibromyoma OR fibromyomas OR fibroma OR fibromas OR "Leiomyoma"[Mesh]) AND (uterus OR uteri OR uterine OR myometrium OR myometrial OR cervix OR cervical OR "Uterus"[Mesh])) AND ((randomized controlled trial [pt] OR controlled clinical trial [pt] OR randomized [tiab] OR placebo [tiab] OR drug therapy [sh] OR randomly [tiab] OR trial [tiab] OR groups [tiab]) NOT (animals [mh] NOT humans [mh]))

### Cochrane

#1 fibroid or fibroids or leiomyoma or leiomyomas or leiomyomata or fibromyoma or fibromyomas or fibroma or fibromas

#2 MeSH descriptor: [Leiomyoma] explode all trees

#3 #1 or #2

#4 uterus or uteri or uterine or myometrium or myometrial OR cervix OR cervical

#5 MeSH descriptor: [Uterus] explode all trees

#6 #4 or #5

#7 #3 and #6

*Embase*

1. (fibroid or fibroids or leiomyoma or leiomyomas or leiomyomata or fibromyoma or fibromyomas or fibroma or fibromas).af.

2. exp leiomyoma/

3. 1 or 2

4. (uterus or uteri or uterine or myometrium or myometrial or cervix or cervical).af.

5. exp uterus/

6. 4 or 5

7. 3 and 6

8. exp uterus myoma/

9. 7 or 8

10. exp crossover-procedure/ or exp double-blind procedure/ or exp randomized controlled trial/ or single-blind procedure/

11. (((((random* or factorial* or crossover* or cross over* or cross-over* or placebo* or double*) adj blind*) or single*) adj blind*) or assign* or allocat* or volunteer*).af.

12. 10 or 11

13. 9 and 12

### Science citation index

#1 TS=(fibroid or fibroids or leiomyoma or leiomyomas or leiomyomata or fibromyoma or fibromyomas or fibroma or fibromas)

#2 TS=(uterus or uteri or uterine or myometrium or myometrial or cervix or cervical)

#3 TS=(random* OR rct* OR crossover OR masked OR blind* OR placebo* OR meta-analysis OR systematic review* OR meta-analys*)

#4 #1 AND #2 AND #3

### WHO Trial registry

fibroid or fibroids or leiomyoma or leiomyomas or leiomyomata or fibromyoma or fibromyomas or fibroma or fibromas

### Other sources

The references of the included trials were searched to identify further trials. Two authors (KG and JV), independently identified the trials for inclusion by screening the titles and abstracts of the references and extracted data related to the outcomes mentioned above and assessed the risk of bias in the trials. All differences in opinion were resolved by discussion until consensus was reached.

## Risk of bias assessment

The risk of bias was assessed according to the guidelines of the Cochrane Collaboration. The assessment of the risk of bias in the trials was based on sequence generation, allocation concealment, blinding of participants, personnel, and outcome assessors, incomplete outcome data, selective outcome reporting, and source of funding bias[1, 2].

## Statistical methods

The software Winbugs 1.4 was used to perform the network meta-analysis using a Bayesian framework. The models used for analysis were based on those available from NICE DSU. Binomial likelihood was used for binary outcomes (such as proportion of people with successful pregnancies), poisson likelihood for count outcomes (such as number of adverse events and for binary outcomes with too many zeros that did not allow the analysis by binomial likelihood), and normal likelihood for continuous outcomes to calculate the odds ratio, rate ratio, and mean difference with 95% credible intervals respectively. Three different starting points or initial values (three chains) were used and a burn-in of 30,000 iterations to ensure that the final results were not dependent on the starting point. A further 30,000 iterations were run to obtain the effect estimates. The choice of fixed-effect model versus random-effects model was based on the deviance information criteria (DIC) as per NICE DSU guidelines[3]. Heterogeneity was assessed using the tau-square (between study variance). The transitivity assumption was tested by identifying whether the inconsistency model resulted in a better model fit which would indicate that there is statistical evidence of incoherence between direct and indirect evidence.

For the continuous outcomes, if the mean values were not available, the medians were used for meta-analysis. If the standard deviations were not available for the continuous outcomes, the standard deviations were calculated according to the guidelines of The Cochrane Collaboration guidelines [2]. This involves assumptions that both groups have the same variance, which may not be true. In order to assess the impact of such imputations, a sensitivity analysis was performed by excluding such trials. A subgroup analysis based on symptom status and menstrual status was planned for the medical versus surgical treatments and different medical treatments but this could not be carried out because of the few trials that could be included in the subgroup analysis. A subgroup analysis based on the type of surgical procedure (hysterectomy versus myomectomy) was performed for the comparison of different medical treatments prior to surgical treatment whenever possible. All the analyses were based on the intention-to-treat principle [4] whenever possible.

The probability of being the best treatment and the probability of being in the best two treatments, best three treatments etc (cumulative ranking probability) [5] was calculated for each outcome.

## Exploration of publication bias and other reporting bias

Exploration of publication bias and other reporting bias was planned using funnel plot where there were at least 10 trials comparing the same intervention and control. Visual inspection of funnel plot asymmetry and Egger's regression approach [6] for funnel plot asymmetry was to be used for identifying the reporting bias. A P-value of 0.10 was considered to be statistically significant.

## Summary of findings tables

Summary of findings tables providing the number of studies and participants included in the network meta-analysis, quality of the evidence based on GRADE methodology [7], the relative effect (odds ratio or rate ratio) or the mean difference for each pairwise comparison, and illustrative absolute effect for odds ratio or rate ratio were created for each outcome based on the mean control group proportion or rate (for odds ratio and rate ratio respectively) and control group mean for mean difference.

1. Lundh A, Sismondo S, Lexchin J, Busuioc OA, Bero L. Industry sponsorship and research outcome. The Cochrane database of systematic reviews. 2012;12:MR000033. doi: 10.1002/14651858.MR000033.pub2. PubMed PMID: 23235689.

2. Higgins J, Green S, (editors). Cochrane Handbook for Systematic Reviews of Interventions 5.1.0 [updated March 2011]. The Cochrane Collaboration, 2011. Available from wwwcochrane-handbookorg.

3. Dias S, Welton N, Sutton A, Ades A. NICE DSU Technical Support Document 1: Introduction to evidence synthesis for decision making. <http://wwwnicedsuorguk/TSD1%20Introductionfinal080512pdf>. 2012 (accessed on 11th March 2014).

4. Newell DJ. Intention-to-treat analysis: implications for quantitative and qualitative research. International Journal of Epidemiology. 1992;21(5):837-41.

5. Salanti G, Ades AE, Ioannidis JP. Graphical methods and numerical summaries for presenting results from multiple-treatment meta-analysis: an overview and tutorial. Journal of clinical epidemiology. 2011;64(2):163-71. doi: 10.1016/j.jclinepi.2010.03.016. PubMed PMID: 20688472.

6. Egger M, Davey Smith G, Schneider M, Minder C. Bias in meta-analysis detected by a simple, graphical test. Bmj. 1997;315(7109):629-34. PubMed PMID: 9310563; PubMed Central PMCID: PMC2127453.

7. Guyatt GH, Oxman AD, Schunemann HJ, Tugwell P, Knottnerus A. GRADE guidelines: a new series of articles in the Journal of Clinical Epidemiology. Journal of clinical epidemiology. 2011;64(4):380-2. doi: 10.1016/j.jclinepi.2010.09.011. PubMed PMID: 21185693.
