# Supplementary material for: Medical Therapies for Uterine Fibroids – A Systematic Review and Network Meta-Analysis of Randomised Controlled Trials
Source: PLoS One. 2016 Feb 26;11(2):e0149631. doi: 10.1371/journal.pone.0149631 (PMC4769153; doi:10.1371/journal.pone.0149631)
Supplement: S2 Appendix — (DOCX) [file pone.0149631.s002.docx]

# Appendix 2: Detailed results

## Medical versus surgical treatment

Parazzini et al, 1999 [1]: Only one trial directly compared medical versus surgical treatment. In this trial, 72 premenopausal women aged more than 45 years (mean age = 50 years) with one or more uterine fibroids > 10 cm in diameter at ultrasound evaluation and had symptomatic menorrhagia lasting three months or more but with a haemoglobin at least 9 g/ dl and were eligible to undergo hysterectomy were included. The medical treatment was Goserelin 3.6 mg (gonatropin releasing hormone analogue) subcutaneously every 4 weeks for 4 months. The control group underwent direct surgery (hysterectomy). The trial was at unclear or high risk of bias in all the domains. Of the 59 participants who were randomised to the medical treatment, 23 participants (23/59 or 39.0% had undergone hysterectomy because of worsening symptoms). The risk ratio of undergoing hysterectomy at 3 years was statistically significantly lower in the medical treatment group than direct surgery group (RR 0.41; 95% CrI 0.29 to 0.57; P < 0.00001). However, this trial did not report any other outcome of interest and so we were unable to determine whether there was any significant difference in mortality, blood transfusion requirements, resources used, or the overall quality of life in the two groups.

Rees et al, 2001 [2]: One other trial compared medical treatment alone versus medical treatment followed by routine surgical treatment. In this trial, 25 premenopausal women who had symptomatic fibroids with uterine size between 12 weeks gestation (255 to 345 ml) and 16 weeks gestation (425 to 575 ml) were included. The medical treatment was Goserelin 3.6 mg (gonatropin releasing hormone analogue) subcutaneously every 4 weeks for 4 months in both groups but only the control group underwent routine surgery. The trial had low risk of bias in the allocation sequence generation, allocation concealment, and missing outcome domains and high risk of bias in the remaining domains. Of the 13 participants who underwent medical treatment alone, 7 required further treatment in the form of hysterectomy (6) and endometrial resection while only one of the 12 participants who underwent medical treatment and endometrial resection required hysterectomy in the control group. The proportion of people who underwent hysterectomy at 12 months was not statistically significant between the medical treatment and medical treatment followed by endometrial resection groups (RR 5.54; 95% CrI 0.78 to 39.57; P = 0.09). Again, this trial did not report any other outcome of interest and so we were unable to determine whether there was any significant difference in mortality, blood transfusion requirements, resources used, or the overall quality of life in the two groups.

The effect estimates of these two studies are given in S1 Figure.

## Medical treatment in women not scheduled to undergo surgery

### Characteristics of included trials

A total of 32 trials were included in the systematic review [3-34]. Eight trials had multiple arms that could be included in the review [4, 6, 9, 10, 15, 18, 20, 25]. Only one of the trials [6] had more than two different treatments [6]. The remaining multiple-armed trials included different doses of the interventional drug and compared it with a control treatment [4, 9, 10, 15, 18, 20, 25]. The different doses of a drug were combined to form a single group in these trials. The remaining trials provided data for two arms. Fourteen trials did not contribute to the meta-analysis. This was because these trials did not report any of the outcomes included in this review [6, 7, 11, 15-19, 21, 26, 27, 32-34].

The characteristics of the patients such as the fibroid size, menstrual status, and whether they were symptomatic, the details of the intervention and control including the dose, frequency, and duration are provided in table 1 in S3 Appendix.

### Risk of bias

The risk of bias in these trials is provided in Supplementary table 2. All the trials were at unclear or high risk of bias.

### Effect estimates

#### Proportion requiring surgery

Five studies reported this outcome [9, 10, 24, 29, 31]. However, only four studies could be included in the network meta-analysis since one of the studies was not connected to the network [31]. All studies reported the number of proportion of women who underwent surgery only until end of treatment. The effect estimates from the individual studies is provided in S2 Figure. The network plot, plot of the probability of best treatment, and plot of the cumulative ranking probability are provided in S3-5 Figures. The summary of findings is provided in table 3 in S3 Appendix. The pairwise effect estimates obtained from network meta-analysis are provided in table 4 in S3 Appendix. The proportion of women requiring surgery was statistically significantly lower in the tibolone/leuprolide group compared to no treatment (OR 0.08; 95% CrI 0.01 to 0.47; probability of being best treatment 0.9652). There were no statistically significant results between any of the other comparisons.

#### Proportion with adverse events

Seven studies reported this outcome [3, 4, 9, 10, 24, 25, 28]. The effect estimates from the individual studies is provided in S6 Figure. The network plot, plot of the probability of best treatment, and plot of the cumulative ranking probability are provided in S7-9 Figures. The summary of findings is provided in table 5 in S3 Appendix. The pairwise effect estimates obtained from network meta-analysis are provided in table 6 in S3 Appendix. None of the comparisons resulted in statistically significant results but the 95% credible intervals were wide.

#### Number with adverse events

Eight studies reported this outcome [3-5, 13, 14, 22, 23, 35]. The adverse events were mostly mild and included hot flushes, headache, breast pain, tenderness or discomfort, abdominal pain, pyrexia, hypercholesterolemia, hypothyroidism, constipation, hypertriglyceridemia, influenza, dizziness, nasopharyngitis, and dysmenorrhea. The effect estimates from the individual studies is provided in S10 Figure. One of the trials was not connected to the network and was not included in the network meta-analysis [5]. The network plot, plot of the probability of best treatment, and plot of the cumulative ranking probability are provided in S11-13 Figures. The summary of findings is provided in table 7 in S3 Appendix. The pairwise effect estimates obtained from network meta-analysis are provided in table 8 in S3 Appendix. As indicated in the table, leuprolide, mifepristone, medroxyprogesterone/leuprolide and raloxiphene/leuprolide had statistically significant more adverse events than placebo group; and leuprolide had statistically significant more adverse events than asoprisnil and mifepristone. There were no statistically significant differences between other comparisons.

#### Quality of life

Six studies reported this outcome [10, 12, 20, 24, 28, 35]. All the trials reported quality of life only for the duration of treatment. Five trials used Uterine Fibroid Symptom Quality of Life scale [10, 12, 20, 28, 35] and one trial used SF-36 quality of life scale [24]. As combination of these two scales would be inappropriate we have presented a narrative summary rather than performing meta-analysis.

#### Haemoglobin

Eight studies reported this outcome [3, 9, 10, 12-14, 23, 30]. The effect estimates from the individual studies is provided in S14 Figure. One trial could not be included in the network meta-analysis because it was not connected to the network [30]. The network plot, plot of the probability of best treatment, and plot of the cumulative ranking probability are provided in S15-17 Figures. The summary of findings is provided in table 9 in S3 Appendix. The pairwise effect estimates obtained from network meta-analysis are provided in table 10 in S3 Appendix. As indicated in the table, leuprolide, mifepristone, raloxifene/leuprolide, and ulipristal statistically significantly improved the haemoglobin levels by about 0.8 to 1 gm/dl compared to placebo group. The other statistically significant comparisons included medroxyprogesterone/leuprolide versus leuprolide (favouring leuprolide), mifepristone versus leuprolide (favouring mifepristone), mifepristone versus medroxyprogesterone/leuprolide (favouring mifepristone), raloxifene/leuprolide versus medroxyprogesterone/leuprolide (favouring raloxifene/leuprolide), ulipristal versus medroxyprogesterone/leuprolide (favouring ulipristal), and ulipristal versus mifepristone (favouring mifepristone). The remaining comparisons were not statistically significant. Standard deviation was imputed in three studies [3, 13, 14]. Excluding these studies did not result in major alterations in conclusions of the comparisons against placebo.

### Results

#### Proportion requiring surgery

A fixed-effects model was used because of near-equivalent DIC values. Tibolone/leuprolide had a 96.5% probability of being the best treatment for reducing the need for surgery. The tau value for between study variability was 0.093.

#### Proportion with adverse events

A fixed-effects model was used because of near-equivalent DIC values. No treatment reached a 45% probability of being the best treatment. The tau value for between study variability was 0.15

#### Number with adverse events

A fixed-effects model was used because of near-equivalent DIC values. Placebo had an 82.8% probability of being the best treatment for preventing adverse events. The tau value for between study variability was 0.003.

### Quality of life

A narrative summary is provided because of inconsistency in reporting measures between the studies reporting this outcome. All scores reported are out of a maximum of 100. Donnez et al. performed a comparison of ulipristal and leuprolide with a resulting quality of life score of 79 (SD 22.7) and 73.2 (SD 23) respectively [10]. Esteve et al. performed a comparison of mifepristone and placebo with a resulting quality of life score of 76.2 (SD 23.4) and 70.2 (SD 22) respectively [35]. Fiscella et al. also performed a comparison of mifepristone and placebo with a resulting quality of life score of 75.8 (SD 16.5) and 39.1 (SD 32.2) respectively[12]. Nieman et al. performed a comparison of ulipristal and placebo with a resulting quality of life score of 27.8 (SD 18.4) and 8.6 (SD 19.4) respectively [20]. Palomba et al. performed a comparison of tibolone and leuprolide with leuprolide alone with a resulting quality of life score of 83.1 (SD 9.4) and 78.5 (SD 10.1) respectively [24]. Roshdy et al. performed a comparison of green tea extract and placebo with a resulting quality of life score of 20.7 (SD 21.1) and 2.19 (SD 17.4) respectively [28]. All six studies show increased quality of life scores with medical treatment. As statistical significance of this is unclear any conclusions would be inappropriate.

#### Haemoglobin

A fixed-effects model was used because of a DIC value of 6.85 compared to 8.67 for the random-effects model. Mifepristone had a greater than 93.9% probability of being the best treatment in terms of increasing haemoglobin levels. Between-study variability was extremely low with a tau value of 0.0009.

## Medical treatment prior to planned surgery

### Characteristics of included trials

A total of 41 trials were included in the systematic review. Eight trials had multiple arms that could be included in the review. Three of these compared two different medical treatments. The remaining multiple-armed trials included different doses of the interventional drug and compared it with a control treatment. The different doses of a drug were combined to form a single group in these trials. Two trials reported data for different patients groups separately. Hudecek 2012 separated data for women who underwent hysterectomy and myomectomy. Stovall 1994 separated data for women with uterine fibroids 14-18 weeks gestation compared to greater than 18 weeks gestation. These patients were included as if they belonged to different trials which were denoted as Hudecek 2012a, Hudecek 2012b, Stovall 1994a and Stovall 1994b. The remaining trials were two-armed. Thirteen trials did not contribute to the meta-analysis. This was because these trials did not report any of the outcomes included in this review.

The characteristics of the patients such as fibroid size, menstrual status, whether they are symptomatic and the details of the intervention and control are provided in table 11 in S3 Appendix.

### Risk of bias

The risk of bias in these trials is provided in table 12 in S3 Appendix. All but one of the studies were at unclear or high risk of bias.

### Effect estimates

#### Mortality

Three trials reported mortality; however there were no deaths in any of the groups meaning meta-analysis could not be performed.

#### Proportion undergoing abdominal myomectomy

All participants underwent either abdominal or laproscopic myomectomy in the trial irrespective of the group to which they belonged. This meant meta-analysis could not be performed.

#### Quality of life

Not reported in any of the trials

#### Amount of blood transfused

Not reported in any of the trials.

#### Cost of treatment

Not reported in any of the trials.

#### Proportion with a successful pregnancy

There were only two trials examining this so no subgroup analysis was performed.

#### Proportion with adverse events

Six studies reported this outcome. The effect estimates from the individual studies is provided in S18 Figure. One trial could not be included in the network meta-analysis because it was not connected to the network. The network plot, plot of the probability of best treatment, and plot of the cumulative ranking probability are provided in S19-21 Figure. The summary of findings is provided in table 13 in S3 Appendix. The pairwise effect estimates obtained from network meta-analysis are provided in table 14 in S3 Appendix. As indicated in the table goserelin statistically significantly increases the proportion of women suffering adverse events compared to the placebo group (OR 6.35; 95% CrI 3.33 to 12.1). The remaining comparisons were not statistically significant.

#### Number with adverse events

Nine studies reported this outcome. The effect estimates from the individual studies is provided in S22 Figure. One trial could not be included in the network meta-analysis because it was not connected to the network. The network plot, plot of the probability of best treatment, and plot of the cumulative ranking probability are provided in S23-25 Figures. The summary of findings is provided in table 15 in S3 Appendix. The pairwise effect estimates obtained from network meta-analysis are provided in table 16 in S3 Appendix. As indicated in the table goserelin and leuprolide had statistically significantly more adverse events than the group with no active treatment. There were no statistically significant differences between other comparisons.

#### Proportion undergoing abdominal hysterectomy

Fourteen studies reported this outcome. The effect estimates from the individual studies is provided in S26 Figure. The network plot, plot of the probability of best treatment, and plot of the cumulative ranking probability are provided in S27-29 Figures. The summary of findings is provided in table 17 in S3 Appendix. The pairwise effect estimates obtained from network meta-analysis are provided in table 18 in S3 Appendix. As indicated in the table leuprolide statistically significantly reduced the proportion of women undergoing abdominal hysterectomy (OR 0.55; 95% CrI 0.4 to 0.75) compared to the group receiving no active treatment. There were no statistically significant differences between other comparisons.

#### Proportion undergoing blood transfusion

Eighteen studies reported this outcome. The effect estimates from the individual studies is provided in S30 Figure. The network plot, plot of the probability of best treatment, and plot of the cumulative ranking probability are provided in S31-33 Figures. The summary of findings is provided in table 19 in S3 Appendix. The pairwise effect estimates obtained from network meta-analysis are provided in table 21 in S3 Appendix. As indicated in the table goserelin and leuprolide significantly reduce the proportion of women receiving a blood transfusion compared to the group receiving no active treatment. There were no statistically significant differences between other comparisons.

#### Hospital stay

Fourteen studies reported this outcome. The effect estimates from the individual studies is provided in S34 Figure. The network plot, plot of the probability of best treatment, and plot of the cumulative ranking probability are provided in S35-37 Figures. The summary of findings is provided in table 21 in S3 Appendix. The pairwise effect estimates obtained from network meta-analysis are provided in table 22 in S3 Appendix. As indicated in the table none of the comparisons resulted in statistically significant results compared to the group receiving no active treatment. Standard deviation was imputed in four studies. Excluding these studies did not result in major alterations in conclusions of the comparisons against the group receiving no active treatment.

#### Operating time

Twenty-one studies reported this outcome. The effect estimates from the individual studies is provided in S38 Figure. The network plot, plot of the probability of best treatment, and plot of the cumulative ranking probability are provided in S39-41 Figures. The summary of findings is provided in table 23 in S3 Appendix. The pairwise effect estimates obtained from network meta-analysis are provided in table 24 in S3 Appendix. As indicated in the table leuprolide statistically significantly reduced operating time (MD -8.56; 95% CrI -15.28 to -1.84) compared to the group receiving no active treatment. There were no statistically significant differences between other comparisons. Standard deviation was imputed in three studies. Excluding these studies did not result in major alterations in the conclusion of the comparisons against the group receiving no active treatment.

#### Haemoglobin

Eight studies reported this outcome. The effect estimates from the individual studies is provided in S42 Figure. The network plot, plot of the probability of best treatment, and plot of the cumulative ranking probability are provided in S43-45 Figures. The summary of findings is provided in table 25 in S3 Appendix. The pairwise effect estimates obtained from network meta-analysis are provided in table 26 in S3 Appendix. As indicated in the table leuprolide, mifepristone, tibolone/leuprolide and triptorelin significantly improved haemoglobin levels by approximately 0.4-1.2 g/dl compared to the group receiving no active treatment. Other statistically significant comparisons included leuprolide versus goserelin (favouring leuprolide) and tibolone/leuprolide versus goserelin (favouring tibolone/leuprolide). The remaining comparisons were not statistically significant.

### Results

#### Proportion with adverse events

A fixed-effects model was used because of near-equivalent DIC values. None of the treatments reached a 35% probability of being the best treatment. The tau value for between study variability was 0.003.

Subgroup analysis was only possible for hysterectomy. This analysis showed no change in the results.

#### Number with adverse events

A fixed-effects model was used because of near-equivalent DIC values. None of the treatments reached a 50% probability of being the best treatment. The tau value for between study variability was 0.008.

Subgroup analysis was only possible for hysterectomy. Again a fixed-effects model was used. Raloxifene had a higher probability of being best at approximately 80%. The tau value for between study variability was 0.014.

#### Proportion undergoing abdominal hysterectomy

The Poisson model was used because of a large number of zero events. A fixed-effects model was used because of near-equivalent DIC values. Raloxifene had a 66.3% probability of being the best treatment. The tau value for between study variability was 0.0095.

Subgroup analysis was performed excluding those undergoing myomectomy. The Poisson model was used because of a large number of zero events. A fixed-effects model was used because of near-equivalent DIC values. For these patients leuprolide had an approximately 65% probability of being the best treatment. The tau value for between study variability was 0.033

#### Proportion undergoing blood transfusion

The Poisson model was used because of a large number of zero events and a lack of convergence with the binomial model. A fixed-effects model was used because of near-equivalent DIC values. None of the treatments had a greater than 55% probability of being the best treatment. The tau value for between study variability was 0.013.

Subgroup analysis was performed for those undergoing hysterectomy. The Poisson model was used because of a large number of zero events and a lack of convergence with the binomial model. A fixed-effect model was used because of near-equivalent DIC values. Triporelin had a greater than 99% probability of being the best treatment for reducing blood transfusion in patients undergoing hysterectomy. The tau value for between study variability was 0.005.

Subgroup analysis was performed for women undergoing myomectomy. The Poisson model was used because of too many zero events and a lack of convergence with the binomial model. A fixed-effect model was used. Triporelin and leuprolide had an approximately 60% probability of being the best treatment for reducing blood transfusion in patients undergoing myomectomy. The tau value for between study variability was 0.008.

#### Hospital stay

A random-effects model was used because of a DIC value of 37.35 compared to 97.52 for the fixed-effect model. Leuprolide had an 82.6% probability of being the best treatment in terms of reducing length of hospital stay. The tau value for between study variability was 0.90. Sensitivity analyses were performed to investigate the large tau value. They showed no change in the results.

Subgroup analysis was performed for women undergoing hysterectomy. A random-effects model was used. None of the treatments reached a 55% probability of being the best treatment. The tau value for between study variability was 1.11.

Subgroup analysis was performed for women undergoing myomectomy. A random-effects model was used. None of the treatments reached a 60% probability of being the best treatment. The tau value for between study variability was 0.80

#### Operating time

A random-effects model was used because of a DIC value of 332.66 compared to 360.18 for the fixed-effect model. None of the treatments had a greater than 40% probability of being the best treatment for reducing operating time. The tau value for between study variability was 4.39. Sensitivity analyses were performed to investigate the large tau value. They showed no change in the result.

Subgroup analysis was performed for women undergoing hysterectomy. A fixed-effect model was used. None of the treatments reached beyond a 65% probability of being the best treatment. The tau value for between study variability was 0.13.

Subgroup analysis was performed for women undergoing myomectomy. A random-effects model was used. None of the treatments had a greater than 65% probability of being the best treatment. The tau value for between study variability was 0.84.

#### Haemoglobin

A fixed-effect model was used because of near-equivalent DIC values. None of the treatments had a greater than 40% probability of being the best treatment for increasing haemoglobin levels. The tau value for between study variability was 0.005.

Subgroup analysis was performed for women undergoing hysterectomy. A fixed-effect model was used. Leuprolide had a greater than 95% probability of being the best treatment for increasing haemoglobin levels in women undergoing hysterectomy. The tau value for between study variability was 0.01.

Subgroup analysis was performed for women undergoing myomectomy. A fixed-effect model was used. Leuprolide had an approximately 65% probability of being the best treatment. The tau value for between study variability was 0.03.

1. Parazzini F, Bortolotti A, Chiantera V, Scollo P, Del Monaco D, Bianchi M, et al. Goserelin acetate to avoid hysterectomy in pre-menopausal women with fibroids requiring surgery. European Journal of Obstetrics Gynecology and Reproductive Biology. 1999;87(1):31-3. doi: 10.1016/s0301-2115(99)00089-5. PubMed PMID: WOS:000083143900005.

2. Rees M, Chamberlain P, Gillmer M. Management of uterine fibroids with goserelin acetate alone or goserelin acetate plus endometrial resection. Gynaecological Endoscopy. 2001;10(1):33-5. doi: 10.1046/j.1365-2508.2001.00396.x. PubMed PMID: WOS:000168262200008.

3. Bagaria M, Suneja A, Vaid NB, Guleria K, Mishra K. Low-dose mifepristone in treatment of uterine leiomyoma: A randomised double-blind placebo-controlled clinical trial. Aust N Z J Obstet Gynaecol. 2009;49(1):77-83. doi: 10.1111/j.1479-828X.2008.00931.x. PubMed PMID: WOS:000263855900016.

4. Chwalisz K, Larsen L, Mattia-Goldberg C, Edmonds A, Elger W, Winkel CA. A randomized, controlled trial of asoprisnil, a novel selective progesterone receptor modulator, in women with uterine leiomyomata. Fertility and sterility. 2007;87(6):1399-412. doi: 10.1016/j.fertnstert.2006.11.094. PubMed PMID: WOS:000247150100024.

5. Constantini S, Anserini P, Valenzano M, Remorgida V, Venturini PL, De Cecco L. Luteinizing hormone-releasing hormone analog therapy of uterine fibroid: Analysis of results obtained with buserelin administered intranasally and goserelin administered subcutaneously as a monthly depot. European Journal of Obstetrics Gynecology and Reproductive Biology. 1990;37(1):63-9. PubMed PMID: 1990206478.

6. Daniels A, Pike M, Daniels J, Spicer D. Treatment with the GnRH agonist (GnRHa) deslorelin (D) and low-dose add-back estradiol (E2) is effective in reducing pain, bleeding and uterine volume(UV) while maintaining bone mineral density (BMD) in women with symptomatic uterine fibroids (UF). Fertility & Sterility. 2002;Vol 78(3 Suppl 1):S65-6, Abstract no: O-170. PubMed PMID: CN-00404980.

7. De Aloysio D, Altieri P, Penacchioni P, Salgarello M, Ventura V. Bleeding patterns in recent postmenopausal outpatients with uterine myomas: Comparison between two regimens of HRT. Maturitas. 1998;29(3):261-4. doi: <http://dx.doi.org/10.1016/S0378-5122%2898%2900014-0>. PubMed PMID: 1998237358.

8. De Falco M, Staibano S, Mascolo M, Mignogna C, Improda L, Ciociola F, et al. Leiomyoma pseudocapsule after pre-surgical treatment with gonadotropin-releasing hormone agonists: Relationship between clinical features and immunohistochemical changes. European Journal of Obstetrics Gynecology and Reproductive Biology. 2009;144(1):44-7. doi: <http://dx.doi.org/10.1016/j.ejogrb.2009.02.006>. PubMed PMID: 2009171587.

9. Donnez J, Tatarchuk TF, Bouchard P, Puscasiu L, Zakharenko NF, Ivanova T, et al. Ulipristal acetate versus placebo for fibroid treatment before surgery. The New England journal of medicine. 2012;366(5):409-20. doi: 10.1056/NEJMoa1103182. PubMed PMID: CN-00804100.

10. Donnez J, Tomaszewski J, Vazquez F, Bouchard P, Lemieszczuk B, Baro F, et al. Ulipristal acetate versus leuprolide acetate for uterine fibroids. New England Journal of Medicine. 2012;366(5):421-32. doi: <http://dx.doi.org/10.1056/NEJMoa1103180>. PubMed PMID: 2012072934.

11. Fedele L, Bianchi S, Raffaelli R, Zanconato G. A randomized study of the effects of tibolone and transdermal estrogen replacement therapy in postmenopausal women with uterine myomas. European journal of obstetrics, gynecology, and reproductive biology. 2000;88(1):91-4. PubMed PMID: CN-00274902.

12. Fiscella K, Eisinger SH, Meldrum S, Feng C, Fisher SG, Guzick DS. Effect of mifepristone for symptomatic leiomyomata on quality of life and uterine size: a randomized controlled trial. Obstetrics and gynecology. 2006;108(6):1381-7. doi: 10.1097/01.AOG.0000243776.23391.7b. PubMed PMID: CN-00574102.

13. Friedman AJ, Barbieri RL, Doubilet PM, Fine C, Schiff I. A randomized, double-blind trial of a gonadotropin releasing-hormone agonist (leuprolide) with or without medroxyprogesterone acetate in the treatment of leiomyomata uteri. Fertility and sterility. 1988;49(3):404-9. PubMed PMID: WOS:A1988M343000004.

14. Friedman AJ, Hoffman DI, Comite F, Browneller RW, Miller JD. Treatment of leiomyomata uteri with leuprolide acetate depot: a double-blind, placebo-controlled, multicenter study. The Leuprolide Study Group. Obstetrics and gynecology. 1991;77(5):720-5. PubMed PMID: CN-00074529.

15. Green LJ, Levy G, Wesley R, Nieman L, Armstrong A. Efficacyof ulipristal acetate forthe treatment of symptomatic uterine leiomyomas in African Americans. Fertility and sterility. 2012;1):S96. doi: <http://dx.doi.org/10.1016/j.fertnstert.2012.07.351>. PubMed PMID: 70871922.

16. Gregoriou O, Vitoratos N, Papadias C, Konidaris S, Costomenos D, Chryssikopoulos A. Effect of tibolone on postmenopausal women with myomas. Maturitas. 1997;27(2):187-91. doi: 10.1016/s0378-5122(97)00036-4. PubMed PMID: WOS:A1997XL50900012.

17. Jirecek S, Lee A, Pavo I, Crans G, Eppel W, Wenzl R. Raloxifene prevents the growth of uterine leiomyomas in premenopausal women. Fertility and sterility. 2004;81(1):132-6. Epub 2004/01/09. PubMed PMID: 14711556.

18. Levy G, Avila N, Armstrong AY, Nieman L. Does the selective progesterone receptor modulator ulipristal normalize the uterine cavity in women with leiomyoma. Reproductive Sciences. 2011;1):95A. doi: <http://dx.doi.org/10.1177/193371912011183s067>. PubMed PMID: 70491653.

19. Morris EP, Rymer J, Robinson J, Fogelman I. Efficacy of tibolone as "add-back therapy" in conjunction with a gonadotropin-releasing hormone analogue in the treatment of uterine fibroids. Fertility and sterility. 2008;89(2):421-8. doi: 10.1016/j.fertnstert.2007.02.064. PubMed PMID: WOS:000253246100024.

20. Nieman LK, Blocker W, Nansel T, Mahoney S, Reynolds J, Blithe D, et al. Efficacy and tolerability of CDB-2914 treatment for symptomatic uterine fibroids: a randomized, double-blind, placebo-controlled, phase IIb study. Fertility and sterility. 2011;95(2):767-U9. doi: 10.1016/j.fertnstert.2010.09.059. PubMed PMID: WOS:000286419000071.

21. Orsini G, Pinto V, Biase S, D'Altorio C, Lanzilotti G. Effects of hormone replacement therapy on postmenopausal women with uterine fibroids. Minerva Ginecologica. 1999;51(11):421-5. PubMed PMID: CN-00295054.

22. Palomba S, Affinito P, Tommaselli GA, Nappi C. A clinical trial of the effects of tibolone administered with gonadotropin-releasing hormone analogues for the treatment of uterine leiomyomata. Fertility and sterility. 1998;70(1):111-8. doi: 10.1016/s0015-0282(98)00128-9. PubMed PMID: WOS:000074479600020.

23. Palomba S, Orio F, Jr., Morelli M, Russo T, Pellicano M, Nappi C, et al. Raloxifene administration in women treated with gonadotropin-releasing hormone agonist for uterine leiomyomas: effects on bone metabolism. The Journal of clinical endocrinology and metabolism. 2002;87(10):4476-81. Epub 2002/10/05. PubMed PMID: 12364422.

24. Palomba S, Orio Jr F, Falbo A, Oppedisano R, Tolino A, Zullo F. Tibolone reverses the cognitive effects caused by leuprolide acetate administration, improving mood and quality of life in patients with symptomatic uterine leiomyomas. Fertility and sterility. 2008;90(1):165-73. doi: <http://dx.doi.org/10.1016/j.fertnstert.2007.05.061>. PubMed PMID: 2008307526.

25. Palomba S, Orio Jr F, Morelli M, Russo T, Pellicano M, Zupi E, et al. Raloxifene administration in premenopausal women with uterine leiomyomas: A pilot study. Journal of Clinical Endocrinology and Metabolism. 2002;87(8):3603-8. doi: <http://dx.doi.org/10.1210/jc.87.8.3603>. PubMed PMID: 2002288881.

26. Parsanezhad ME, Azmoon M, Alborzi S, Rajaeefard A, Zarei A, Kazerooni T, et al. A randomized, controlled clinical trial comparing the effects of aromatase inhibitor (letrozole) and gonadotropin-releasing hormone agonist (triptorelin) on uterine leiomyoma volume and hormonal status. Fertility and sterility. 2010;93(1):192-8. doi: 10.1016/j.fertnstert.2008.09.064. PubMed PMID: WOS:000273601200029.

27. Polatti F, Viazzo F, Colleoni R, Nappi RE. Uterine myoma in postmenopause: a comparison between two therapeutic schedules of HRT. Maturitas. 2000;37(1):27-32. doi: 10.1016/s0378-5122(00)00159-6. PubMed PMID: WOS:000165650900003.

28. Roshdy E, Rajaratnam V, Maitra S, Sabry M, Ait Allah AS, Al-Hendy A. Treatment of symptomatic Uterine fibroids with green tea extract: A pilot randomized controlled clinical study. International Journal of Women's Health. 2013;5(1):477-86. doi: <http://dx.doi.org/10.2147/IJWH.S41021>. PubMed PMID: 2013496484.

29. Sadan O, Ginath S, Sofer D, Rotmensch S, Debby A, Glezerman M, et al. The role of tamoxifen in the treatment of symptomatic uterine leiomyomata -- a pilot study. European journal of obstetrics, gynecology, and reproductive biology. 2001;96(2):183-6. PubMed PMID: CN-00348348.

30. Sayed GH, Zakherah MS, El-Nashar SA, Shaaban MM. A randomized clinical trial of a levonorgestrel-releasing intrauterine system and a low-dose combined oral contraceptive for fibroid-related menorrhagia. Int J Gynecol Obstet. 2011;112(2):126-30. doi: 10.1016/j.ijgo.2010.08.009. PubMed PMID: WOS:000286704100011.

31. Sayyah-Melli M, Tehrani-Gadim S, Dastranj-Tabrizi A, Gatrehsamani F, Morteza G, Ouladesahebmadarek E, et al. Comparison of the effect of gonadotropin-releasing hormone agonist and dopamine receptor agonist on uterine myoma growth Histologic, sonographic, and intra-operative changes. Saudi Med J. 2009;30(8):1024-33. PubMed PMID: WOS:000270844200007.

32. Schlaff WD, Zerhouni EA, Huth JA, Chen J, Damewood MD, Rock JA. A placebo-controlled trial of a depot gonadotropin-releasing hormone analogue (leuprolide) in the treatment of uterine leiomyomata. Obstetrics and gynecology. 1989;74(6):856-62. PubMed PMID: CN-00063914.

33. Simsek T, Karakus C, Trak B. Impact of different hormone replacement therapy regimens on the size of myoma uteri in postmenopausal period: tibolone versus transdermal hormonal replacement system. Maturitas. 2002;42(3):243-6. PubMed PMID: CN-00405090.

34. Varun N, Kumar A, Prasad S. Effect of low dose mifepristone on uterine leiomyoma in reproductive age group. Fertility and sterility. 2013;1):S78. doi: <http://dx.doi.org/10.1016/j.fertnstert.2013.07.1924>. PubMed PMID: 71164058.

35. Esteve JL, Acosta R, Perez Y, Rodriguez B, Seigler I, Sanchez C, et al. Mifepristone versus placebo to treat uterine myoma: a double-blind, randomized clinical trial. International journal of women's health. 2013;5:361-9. Epub 2013/07/12. doi: 10.2147/ijwh.s42770. PubMed PMID: 23843709; PubMed Central PMCID: PMCPmc3702243.
