# Supplementary material for: Medical Therapies for Uterine Fibroids – A Systematic Review and Network Meta-Analysis of Randomised Controlled Trials
Source: PLoS One. 2016 Feb 26;11(2):e0149631. doi: 10.1371/journal.pone.0149631 (PMC4769153; doi:10.1371/journal.pone.0149631)
Supplement: S3 Appendix — (DOCX) [file pone.0149631.s003.docx]

# Appendix 3: Tables

## Table 1: Medical treatment in women not scheduled to undergo surgery – Characteristics of included studies

| **Study** | **Fibroid size** | **Menstrual status** | **Symptomatic/asymptomatic** | **Intervention name** | **Intervention class** | **Intervention dose and route** | **Intervention duration** | **Intervention number of people randomised** | **Control name** | **Control class** | **Control dose and route** | **Control duration** | **Control number of people randomised** | **Number of people excluded from the study** | **Outcomes reported** |
| --- | --- | --- | --- | --- | --- | --- | --- | --- | --- | --- | --- | --- | --- | --- | --- |
| Bagaria 2009[1] | Not stated | Not stated | Symptomatic | Mifepristone | Progesterone antagonist | 10 mg/day orally | 90 days | 19 | Placebo |  |  |  | 16 | 5 | Proportion of patients with adverse events, number of adverse events, haemoglobin |
| Carbonell Esteve 2013[2] | Not stated | Not stated | Symptomatic | Mifepristone | Progesterone antagonist | 5 mg/day orally | 90 days | 58 | Placebo |  |  |  | 47 | 19 | Number of adverse events, quality of life |
| Chwalisz 2007[3] | > 3 cm diameter | Not stated | Not stated | Asoprisnil | Selective progesterone receptor modulator | 25 mg, 10 mg, or 5 mg/day orally (randomised to three different doses) | 12 weeks | 98 | Placebo |  |  | 12 weeks | 31 | 0 | Proportion of patients with adverse events, number of adverse events |
| Constantini 1990[4] | > 5 cm diameter | Not stated | Not stated | Buserelin | Gonatropin releasing hormone analogue | 500 µg thrice a day subcutaneously for first 10 days followed by 200 μg four times a day intranasally | 6 months | 21 | Goserelin | Gonatropin releasing hormone analogue | 3.6 mg subcutaneously every 4 weeks | 6 months | 21 |  | Number of adverse events |
| Daniels 2002[5] | Not stated | Not stated | Symptomatic | Deslorelin | Gonadotropin releasing hormone analogue | 1 mg once a day intranasally | 12 months | Not stated | Second to fourth controls: Deslorelin/estradiol Fifth control: Placebo | Gonadotropin releasing hormone analogue/oestrogen | Second to fourth control: 1 mg once a day intranasally/75 mg or 50 mg or 25 mg (3 different doses) transdermal patch | 12 months | Not stated |  | None of the outcomes of interest for this review were reported |
| De Aloysio 1998[6] | Not stated | Not stated | Not stated | Tibolone | Estrogen agonist | 2.5 mg/day orally | 8 weeks | 24 | Conjugated equine estrogens and medroxyprogesterone acetate | Estrogen and progesterone agonist | 0.625 mg/0.5 mg once a day orally | 8 weeks | 23 | 3 | None of the outcomes of interest for this review were reported |
| Donnez 2012a[7] | > 3 cm and < 10 cm (16 weeks size of gestation) | Premenopausal | Symptomatic | Ulipristal acetate | Selective progesterone receptor modulator | 10 mg or 5 mg/day orally (two different dose groups) | 13 weeks | 189 | Placebo |  |  |  | 48 | 5 | Proportion of patients requiring surgery, proportion of patients with adverse events, haemoglobin |
| Donnez 2012b[8] | > 3 cm and < 10 cm (16 weeks size of gestation) | Postmenopausal | Symptomatic | Ulipristal acetate | Selective progesterone receptor modulator | 10 mg or 5 mg/day orally (two different dose groups) | 13 weeks | 200 | Leuprolide acetate | Gonadotropin hormone analogue | 3.75 mg intramuscularly once a month | 13 weeks | 101 | 41 | Proportion of patients requiring surgery, proportion of patients with adverse events, quality of life, haemoglobin |
| Fedele 2000[9] | > 3 cm diameter | Not stated | Symptomatic | Estradiol/medroxyprogesterone acetate | Estrogen/progesterone agonist | 50 mg/day orally for 12 days per month | 12 months | 19 | Tibolone | Estrogen agonist | 2.5 mg/day orally | 12 months | 18 | 1 | None of the outcomes of interest for this review were reported |
| Fiscella 2006[10] | 160 ml or more/>2.5 cm | Premenopausal | Symptomatic | Mifepristone | Progesterone antagonist | 5 mg/day orally | 6 months | 22 | Placebo |  |  |  | 20 | 0 | Quality of life, haemoglobin |
| Friedman 1988[11] | > 2.5 cm in diameter (or the uterus was at least twice normal volume) | Premenopausal | Not stated | Medroxyprogesterone acetate/leuprolide | Progesterone agonist/gonatrotopin releasing hormone analogue | 20 mg oral/0.5 mg subcutaneously daily | 24 weeks | 9 | Leuprolide/Placebo | Gonatrotopin releasing hormone analogue | 0.5 mg subcutaneously daily | 24 weeks | 7 |  | Number of adverse events, haemoglobin |
| Friedman 1991[12] | > 3 cm diameter | Premenopausal | Not stated | Leuprolide acetate | Gonadotropin hormone analogue | 3.75 mg intramuscularly every 4 weeks | 24 weeks | 60 | Placebo |  |  |  | 64 | 4 | Number of adverse events, haemoglobin |
| Green 2002[13] | Not stated | Not stated | Not stated | Ulipristal acetate | Selective progesterone receptor modulator | 20 mg or 10 mg (two groups at different doses) (further details not known) | not stated | Not stated | Placebo |  |  |  | Not stated |  | None of the outcomes of interest for this review were reported |
| Gregoriou 1997[14] | > 2 cm | Not stated | Asymptomatic | Tibolone | Estrogen agonist | 20 mg/day orally | 12 months | 20 | No treatment |  |  |  | 20 |  | None of the outcomes of interest for this review were reported |
| Jirecek 2004[15] | Not stated | Premenopausal | Asymptomatic | Raloxiphene | Selective estrogen receptor modulator | 180 mg/day orally | 3 months | 11 | No treatment |  |  |  | 12 | 2 | None of the outcomes of interest for this review were reported |
| Levy 2011[16] | > 2 cm diameter | Not stated | Symptomatic | Ulipristal acetate | Selective progesterone-receptor modulator | 20 mg or 10 mg/day (two different groups) orally | 3 menstural cycles | 19 | Placebo |  |  |  | 9 |  | None of the outcomes of interest for this review were reported |
| Morris 2008[17] | Not stated | Premenopausal | Symptomatic | Tibolone/goserelin | Estrogen agonist/gonatrotopin releasing hormone analogue | 2.5 mg orally/3.6 mg subcutaneously every 4 weeks | 6 months | 25 | Goserelin/Placebo | Gonatrotopin releasing hormone analogue | 3.6 mg subcutaneously every 4 weeks | 6 months | 25 | 4 | None of the outcomes of interest for this review were reported |
| Nieman 2011[18] | Not stated | Premenopausal | Not stated | Ulipristal acetate | Selective progesterone-receptor modulator | 20 mg or 10 mg/day (two different groups) orally | 3 menstrual cycles | 26 | Placebo |  |  |  | 12 | 4 | Quality of life |
| Orsini 1999[19] | Not stated | Not stated | Asymptomatic | E2 50/medroxyprogesterone acetate | Estrogen/progesterone agonist | 50 pg transdermal/5 mg once a day | 1 year | 15 | Tibolone | Estrogen agonist | 2.5 mg/day orally | 1 year | 15 | 1 | None of the outcomes of interest for this review were reported |
| Palomba 1998[20] | Not stated | Not stated | Symptomatic | Tibolone/leuprolide | Estrogen agonist/gonatrotopin releasing hormone analogue | 2.5 mg orally/3.75 mg intramuscularly every 4 weeks | 6 months | 24 | Leuprolide/Placebo | Gonatrotopin releasing hormone analogue | 3.75 mg intramuscular every 4 weeks | 6 months | 25 | 2 | Proportion of patients requiring surgery, number of adverse events |
| Palomba 2002b[21] | > 2 cm | Premenopausal | Not stated | Raloxiphene | Selective estrogen receptor modulator | 180 mg or 60 mg/day (two different doses) orally | 6 cycles of 28 days each | 59 | Placebo |  |  |  | 29 | 9 | Proportion of patients with adverse events |
| Palomba 2002c[22] | Not stated | Not stated | Symptomatic | Raloxiphene/leuprolide | Selective estrogen receptor modulator/gonatrotopin releasing hormone analogue | 60 mg/day orally/3.75 mg intramuscular every 4 weeks | 6 cycles of 28 days each | 46 | Leuprolide/Placebo | Gonatrotopin releasing hormone analogue | 3.75 mg intramuscular every 4 weeks | 6 cycles of 28 days each | 45 | 0 | Number of adverse events, haemoglobin |
| Palomba 2008[23] | Not stated | Not stated | Symptomatic | Tibolone/leuprolide | Estrogen agonist/gonatrotopin releasing hormone analogue | 2.5 mg orally/11.25 mg intramuscularly every 3 months | 6 months | 55 | Leuprolide/Placebo | Gonatrotopin releasing hormone analogue | 11.25 mg intramuscular every 3 months | 6 months | 55 |  | Proportion of patients with adverse events, quality of life |
| Parsanezhad 2010[24] | > 5 cm diameter | Not stated | Symptomatic | Letrozole | Aromatase inhibitor | 2.5 mg/ day orally | not stated | 33 | Triptorelin | Gonatropin releasing hormone analogue | 3.75 mg intramuscularly once a month | not stated | 27 | 10 | None of the outcomes of interest for this review were reported |
| Polatti 2000[25] | Not stated | Menopausal | Not stated | Oestradiol valerate/cyproterone acetate | Oestrogen/antiandrogen drug with additional progestogen and antigonadotropic effect | 2 mg/1 mg for 21 days, followed by a therapy break of 7 days | not stated | 38 | E2/medroxy progesterone acetate | Estrogen/progesterone | 50 μg transdermally for 21 days/10 mg per day orally from day 10 to 21, followed by a 7-day therapy break | not stated | 36 | 6 | None of the outcomes of interest for this review were reported |
| Roshdy 2013[26] | > 2 cm | Premenopausal | Symptomatic | Green tea extract | Polyphenols and epigallocatechin gallate - antioxidants | 800 mg/ day | not stated | 22 | Placebo |  |  |  | 11 | 6 | Proportion of patients with adverse events, quality of life |
| Sadan 2001[27] | Not stated | Premenopausal | Symptomatic | Tamoxifen | Estrogen antagonist | 20 mg/day orally | 6 months | 10 | Placebo |  |  |  | 10 | 0 | Proportion of patients requiring surgery |
| Sayed 2011[28] | Not stated | Premenopausal | Not stated | Levonorgestrel intrauterine system | Progesterone | not stated | not stated | 29 | Ethinyl estradial/levonorgestrel | Estrogens/progesterone | 30 μg/150 μg orally | not stated | 29 | 0 | Haemoglobin |
| Sayyah-Melli 2009[29] | Not stated | Not stated | Symptomatic | Triptorelin | Gonatrotropin releasing hormone analogue | 3.75 mg every 28 days (route not stated) | 4 cycles | 30 | Cabergoline | Dopamine agonist | 0.5 mg once a week (route not stated) | 6 weeks | 30 |  | Proportion of patients requiring surgery |
| Schlaff 1989[30] | > 3.5 cm | Not stated | Not stated | Leuprolide acetate | Gonadotropin hormone analogue | 3.75 mg intramuscular every 4 weeks | 6 cycles of 28 days each | 5 | Placebo |  |  |  | 6 | 1 | None of the outcomes of interest for this review were reported |
| Simsek 2002[31] | Not stated | Postmenopausal | Not stated | Tibolone | Estrogen agonist | 2.5 mg/day orally | not stated | 24 | Estradiol/norethisterone acetate | Estrogen/progesterone | 0.05 mg for 4 week/0.25 mg for 2 weeks transdermally | not stated | 22 | 10 | None of the outcomes of interest for this review were reported |
| Varun 2003[32] | Not stated | Not stated | Symptomatic | Mifepristone | Progesterone antagonist | 10 mg/day orally | 90 days | Not stated | Placebo |  |  |  | Not stated |  | None of the outcomes of interest for this review were reported |

##

## Table 2: Medical treatment in women not scheduled to undergo surgery – Risk of bias

| **Study name** | **Random sequence generation** | **Allocation concealment** | **Blinding of patients and healthcare providers** | **Blinding of outcome assessors** | **Missing outcome bias** | **Selective outcome reporting** | **Source of funding** | **Overall risk of bias** |
| --- | --- | --- | --- | --- | --- | --- | --- | --- |
| Bagaria 2009[1] | + | + | + | + | - | - | ? | Unclear or high |
| Carbonell Esteve 2013[2] | ? | + | + | + | - | - | ? | Unclear or high |
| Chwalisz 2007[3] | + | ? | ? | ? | + | - | - | Unclear or high |
| Constantini 1990[4] | ? | ? | ? | ? | ? | - | ? | Unclear or high |
| Daniels 2002[5] | ? | ? | ? | ? | ? | - | - | Unclear or high |
| De Aloysio 1998[6] | ? | ? | ? | ? | - | - | + | Unclear or high |
| Donnez 2012a[7] | + | + | + | + | - | + | - | Unclear or high |
| Donnez 2012b[8] | + | + | ? | ? | - | + | - | Unclear or high |
| Fedele 2000[9] | + | ? | ? | ? | - | - | ? | Unclear or high |
| Fiscella 2006[10] | + | + | + | + | + | - | - | Unclear or high |
| Friedman 1988[11] | ? | ? | ? | ? | ? | - | - | Unclear or high |
| Friedman 1991[12] | ? | ? | + | + | - | - | - | Unclear or high |
| Green 2002[13] | ? | ? | ? | ? | ? | - | - | Unclear or high |
| Gregoriou 1997[14] | ? | ? | ? | ? | ? | - | ? | Unclear or high |
| Jirecek 2004[15] | ? | ? | ? | ? | - | - | ? | Unclear or high |
| Levy 2011[16] | ? | ? | ? | ? | ? | - | ? | Unclear or high |
| Morris 2008[17] | + | + | ? | ? | - | - | ? | Unclear or high |
| Nieman 2011[18] | + | + | + | + | ? | - | ? | Unclear or high |
| Orsini 1999[19] | ? | ? | ? | ? | ? | - | ? | Unclear or high |
| Palomba 1998[20] | + | + | + | + | - | + | + | Unclear or high |
| Palomba 2002b[21] | + | + | - | + | - | - | + | Unclear or high |
| Palomba 2002c[22] | + | + | - | + | - | + | + | Unclear or high |
| Palomba 2008[23] | + | + | - | + | + | + | + | Unclear or high |
| Parsanezhad 2010[24] | + | ? | ? | + | - | - | ? | Unclear or high |
| Polatti 2000[25] | + | ? | ? | ? | - | - | ? | Unclear or high |
| Roshdy 2013[26] | + | + | + | + | - | + | + | Unclear or high |
| Sadan 2001[27] | ? | ? | ? | ? | + | - | ? | Unclear or high |
| Sayed 2011[28] | + | + | - | - | + | - | - | Unclear or high |
| Sayyah-Melli 2009[29] | ? | ? | ? | ? | ? | - | ? | Unclear or high |
| Schlaff 1989[30] | ? | ? | ? | ? | ? | - | ? | Unclear or high |
| Simsek 2002[31] | ? | ? | ? | ? | - | - | ? | Unclear or high |
| Varun 2003[32] | ? | ? | ? | ? | ? | - | - | Unclear or high |

***tables 3 to 26***

Key:

OR = odds ratio

RaR = rate ratio

MD = mean difference

CrI = credible intervals

Tau = between study standard-deviation

## Table 3: Medical treatment in women not scheduled to undergo surgery – Proportion undergoing surgery – summary of findings table

| **Proportion requiring surgery (4 trials; 596 participants)** | | |
| --- | --- | --- |
| **Overall quality of evidence** | **Very low** | |
| **Groups** | **Illustrative risk** | **Treatment effect** |
| Assumed risk in control group (Placebo) | 448 per 1000 | - |
| Corresponding risk in Ulipristal | 531 per 1000 (372 to 685) | OR 1.40; 95% CrI 0.73 to 2.68 |
| Corresponding risk in Leuprolide | 540 per 1000 (342 to 727) | OR 1.45; 95% CrI 0.64 to 3.28 |
| Corresponding risk in Tibolone+leuprolide | 61 per 1000 (11 to 276) | **OR 0.08; 95% CrI 0.01 to 0.47** |
| Corresponding risk in Tamoxifen | 445 per 1000 (93 to 862) | OR 0.99; 95% CrI 0.13 to 7.71 |

## Table 4: Medical treatment in women not scheduled to undergo surgery – Proportion undergoing surgery – pairwise comparisons

|  | **Ulipristal** | **Leuprolide** | **Tibolone+leuprolide** | **Tamoxifen** |
| --- | --- | --- | --- | --- |
| **Placebo** | OR 1.40; 95% CrI 0.73 to 2.68 | OR 1.45; 95% CrI 0.64 to 3.28 | **OR 0.08; 95% CrI 0.01 to 0.47** | OR 0.99; 95% CrI 0.13 to 7.71 |
| **Ulipristal** | - | OR 1.04; 95% CrI 0.36 to 2.95 | **OR 0.06; 95% CrI 0.01 to 0.38** | OR 0.71; 95% CrI 0.08 to 6.1 |
| **Leuprolide** | - | - | **OR 0.06; 95% CrI 0.01 to 0.39** | OR 0.68; 95% CrI 0.07 to 6.22 |
| **Tibolone+leuprolide** | - | - | - | OR 12.22; 95% CrI 0.81 to 183.49 |

Tau = 0.093

## Table 5: Medical treatment in women not scheduled to undergo surgery – Proportion with adverse events – summary of findings table

| **Proportion adverse events (7 trials; 937 participants)** | | |
| --- | --- | --- |
| **Overall quality of evidence** | **Very low** |  |
| **Groups** | **Illustrative risk** | **Treatment effect** |
| Assumed risk in control group (Placebo) | 321 per 1000 | - |
| Corresponding risk in Asoprisnil | 430 per 1000 (239 to 645) | OR 1.60; 95% CrI 0.66 to 3.85 |
| Corresponding risk in Green tea extract | 193 per 1000 (1 to 991) | OR 0.51; 95% CrI 0 to 240.82 |
| Corresponding risk in Leuprolide | 498 per 1000 (287 to 710) | OR 2.10; 95% CrI 0.85 to 5.19 |
| Corresponding risk in Mifepristone | 286 per 1000 (1 to 995) | OR 0.85; 95% CrI 0 to 410.32 |
| Corresponding risk in Raloxiphene | 386 per 1000 (30 to 927) | OR 1.33; 95% CrI 0.07 to 27 |
| Corresponding risk in Tibolone+leuprolide | 617 per 1000 (234 to 894) | OR 3.4; 95% CrI 0.65 to 17.91 |
| Corresponding risk in Ulipristal | 381 per 1000 (245 to 539) | OR 1.30; 95% CrI 0.69 to 2.47 |

##

## Table 6: Medical treatment in women not scheduled to undergo surgery – Proportion with adverse events – pairwise comparisons

|  | **Asoprisnil** | **Green tea extract** | **Leuprolide** | **Mifepristone** | **Raloxiphene** | **Tibolone+leuprolide** | **Ulipristal** |
| --- | --- | --- | --- | --- | --- | --- | --- |
| **Placebo** | OR 1.60; 95% CrI 0.66 to 3.85 | OR 0.51; 95% CrI 0 to 240.82 | OR 2.10; 95% CrI 0.85 to 5.19 | OR 0.85; 95% CrI 0 to 410.32 | OR 1.33; 95% CrI 0.07 to 27 | OR 3.40; 95% CrI 0.65 to 17.91 | OR 1.30; 95% CrI 0.69 to 2.47 |
| **Asoprisnil** | - | OR 0.32; 95% CrI 0 to 160.34 | OR 1.31; 95% CrI 0.37 to 4.63 | OR 0.53; 95% CrI 0 to 273.15 | OR 0.83; 95% CrI 0.04 to 19.15 | OR 2.13; 95% CrI 0.32 to 13.93 | OR 0.81; 95% CrI 0.27 to 2.42 |
| **Green tea extract** | - | - | OR 4.16; 95% CrI 0.01 to 2114.37 | OR 1.68; 95% CrI 0 to 10389.61 | OR 2.63; 95% CrI 0 to 2510.69 | OR 6.73; 95% CrI 0.01 to 3992.14 | OR 2.58; 95% CrI 0.01 to 1268.11 |
| **Leuprolide** | - | - | - | OR 0.40; 95% CrI 0 to 208.56 | OR 0.63; 95% CrI 0.03 to 14.68 | OR 1.62; 95% CrI 0.24 to 10.72 | OR 0.62; 95% CrI 0.2 to 1.88 |
| **Mifepristone** | - | - | - | - | OR 1.57; 95% CrI 0 to 1518.08 | OR 4.01; 95% CrI 0.01 to 2416.38 | OR 1.54; 95% CrI 0 to 767.91 |
| **Raloxiphene** | - | - | - | - | - | OR 2.56; 95% CrI 0.08 to 79.89 | OR 0.98; 95% CrI 0.05 to 21.33 |
| **Tibolone+leuprolide** | - | - | - | - | - | - | OR 0.38; 95% CrI 0.06 to 2.27 |

Tau = 0.15

## Table 7: Medical treatment in women not scheduled to undergo surgery – Number of adverse events – summary of findings table

| **Number of adverse events (7 trials; 551 participants)** | | |
| --- | --- | --- |
| **Overall quality of evidence** | **Very low** | |
| **Groups** | **Illustrative risk** | **Treatment effect** |
| Assumed risk in control group (Placebo) | 714 per 1000 | - |
| Corresponding risk in Asoprisnil | 782 per 1000 (709 to 842) | RaR 1.44; 95% CrI 0.97 to 2.13 |
| Corresponding risk in Leuprolide | 933 per 1000 (901 to 955) | **RaR 5.57; 95% CrI 3.63 to 8.57** |
| Corresponding risk in Medroxyprogesterone+leuprolide | 893 per 1000 (734 to 962) | **RaR 3.33; 95% CrI 1.1 to 10.03** |
| Corresponding risk in Mifepristone | 790 per 1000 (732 to 839) | **RaR 1.51; 95% CrI 1.09 to 2.08** |
| Corresponding risk in Raloxiphene+leuprolide | 904 per 1000 (727 to 971) | **RaR 3.78; 95% CrI 1.07 to 13.41** |
| Corresponding risk in Tibolone+leuprolide | 898 per 1000 (532 to 985) | RaR 3.51; 95% CrI 0.46 to 27.09 |

## Table 8: Medical treatment in women not scheduled to undergo surgery – Number of adverse events – pairwise comparisons

|  | **Asoprisnil** | **Leuprolide** | **Medroxyprogesterone+leuprolide** | **Mifepristone** | **Raloxiphene+leuprolide** | **Tibolone+leuprolide** |
| --- | --- | --- | --- | --- | --- | --- |
| **Placebo** | OR 1.44; 95% CrI 0.97 to 2.13 | **OR 5.57; 95% CrI 3.63 to 8.57** | **OR 3.33; 95% CrI 1.1 to 10.03** | **OR 1.51; 95% CrI 1.09 to 2.08** | **OR 3.78; 95% CrI 1.07 to 13.41** | OR 3.51; 95% CrI 0.46 to 27.09 |
| **Asoprisnil** | - | **OR 3.87; 95% CrI 2.16 to 6.92** | OR 2.31; 95% CrI 0.72 to 7.45 | OR 1.05; 95% CrI 0.63 to 1.74 | OR 2.63; 95% CrI 0.7 to 9.88 | OR 2.44; 95% CrI 0.31 to 19.52 |
| **Leuprolide** | - | - | OR 0.6; 95% CrI 0.18 to 1.95 | **OR 0.27; 95% CrI 0.16 to 0.46** | OR 0.68; 95% CrI 0.18 to 2.58 | OR 0.63; 95% CrI 0.08 to 5.08 |
| **Medroxyprogesterone+leuprolide** | - | - | - | OR 0.45; 95% CrI 0.14 to 1.43 | OR 1.14; 95% CrI 0.21 to 6.1 | OR 1.06; 95% CrI 0.1 to 10.77 |
| **Mifepristone** | - | - | - | - | OR 2.51; 95% CrI 0.68 to 9.25 | OR 2.33; 95% CrI 0.29 to 18.42 |
| **Raloxiphene+leuprolide** | - | - | - | - | - | OR 0.93; 95% CrI 0.08 to 10.26 |

Tau =0.003

## Table 9: Medical treatment in women not scheduled to undergo surgery – Haemoglobin – summary of findings table

| **Haemoglobin (7 trials; 884 participants)** | | |
| --- | --- | --- |
| **Overall quality of evidence** | **Very low** | |
| **Groups** | **Illustrative means (in gm/dl)** | **Treatment effect** |
| Assumed mean in control group (Placebo) | 12 | - |
| Mean in Leuprolide | 0.77 higher (0.37 higher to 1.17 higher) | **MD 0.77; 95% CrI 0.37 to 1.17** |
| Mean in Medroxyprogesterone+leuprolide | 0.03 lower (0.7 lower to 0.64 higher) | MD -0.03; 95% CrI -0.7 to 0.64 |
| Mean in Mifepristone | 1.88 higher (1.06 higher to 2.69 higher) | **MD 1.88; 95% CrI 1.06 to 2.69** |
| Mean in Raloxiphene+leuprolide | 0.97 higher (0.23 higher to 1.7 higher) | **MD 0.97; 95% CrI 0.23 to 1.7** |
| Mean in Ulipristal | 0.96 higher (0.61 higher to 1.31 higher) | **MD 0.96; 95% CrI 0.61 to 1.31** |

## Table 10: Medical treatment in women not scheduled to undergo surgery – Haemoglobin – pairwise comparisons

|  | **Leuprolide** | **Medroxyprogesterone/leuprolide** | **Mifepristone** | **Raloxiphene/leuprolide** | **Ulipristal** |
| --- | --- | --- | --- | --- | --- |
| **Placebo** | **MD 0.77; 95% CrI 0.37 to 1.17** | MD -0.03; 95% CrI -0.7 to 0.64 | **MD 1.88; 95% CrI 1.06 to 2.69** | **MD 0.97; 95% CrI 0.23 to 1.7** | **MD 0.96; 95% CrI 0.61 to 1.31** |
| **Leuprolide** | - | **MD -0.8; 95% CrI -1.58 to -0.02** | **MD 1.11; 95% CrI 0.2 to 2.02** | MD 0.2; 95% CrI -0.63 to 1.04 | MD 0.19; 95% CrI -0.34 to 0.72 |
| **Medroxyprogesterone/leuprolide** | - | - | **MD 1.91; 95% CrI 0.85 to 2.97** | **MD 1; 95% CrI 0.01 to 2** | **MD 0.99; 95% CrI 0.24 to 1.75** |
| **Mifepristone** | - | - | - | MD -0.91; 95% CrI -2.01 to 0.2 | **MD -0.92; 95% CrI -1.81 to -0.03** |
| **Raloxiphene/leuprolide** | - | - | - | - | MD -0.01; 95% CrI -0.83 to 0.8 |

Tau = 0.0009

## Table 11: Medical treatment prior to planned surgery – characteristics of included studies

| **Study** | **Fibroid size** | **Menstural status** | **Symptomatic/asymptomatic** | **Type of surgery** | **Intervention name** | **Intervention class** | **Intervention dose and route** | **Intervention duration (months)** | **Intervention number of people randomised** | **Control name** | **Control class** | **Control dose and route** | **Control duration (months)** | **Control number of people randomised** |
| --- | --- | --- | --- | --- | --- | --- | --- | --- | --- | --- | --- | --- | --- | --- |
| Audebert 1994 |  |  |  | Hysterectomy or myomectomy | Goserelin | Gonadotropin releasing hormone analogue | 3.6 mg subcutaneous monthly | 3 | 20 | No treatment |  |  |  | 27 |
| Balasch 1995 | > 12 weeks gestation in size | Premenopausal | Symptomatic | Abdominal hysterectomy | Triptorelin | Gonadotropin releasing hormone analogue | 3.75 mg intramuscular monthly | 2 | 23 | No treatment |  |  |  | 27 |
| Barbieri 1993 |  | Premenopausal | Symptomatic | Myomectomy | Leuprolide | Gonadotropin releasing hormone analogue | 3.75 mg intramuscular every 4 weeks | 4 | 7 | Placebo |  |  |  | 8 |
| Baytur 2007 |  |  | Symptomatic | Hysterectomy | Goserelin | Gonadotropin releasing hormone analogue | 3.6 mg subcutaneous every 4 weeks | 3 | 16 | Raloxifene | Selective estrogen receptor modulator | 60 mg/day daily | 3 | 16 |
| Bustos-Lopez 1995 |  | Premenopausal |  | Laparoscopic myomectomy | Naferelin | Gonadotropin releasing hormone analogue | 200 pg intranasal twice a day | 3 | 13 | No treatment |  |  |  | 15 |
| Campo 1999 |  |  | Symptomatic | Laparoscopic myomectomy | Triptorelin | Gonadotropin releasing hormone analogue | 3.75 mg intramuscular every 4 weeks | 3 | 30 | No treatment |  |  |  | 30 |
| Coddington 2009 |  | Premenopausal | Symptomatic | Abdominal myomectomy | Leuprolide | Gonadotropin releasing hormone analogue | 3.75 mg intramuscular every 4 weeks | 3 | 10 | Placebo |  |  |  | 10 |
| D'Anna 1994 | > 3 cm or uterine volume double the normal size | Premenopausal |  | Hysterectomy | Leuprolide | Gonadotropin releasing hormone analogue | 3.75 mg intramuscular monthly | 2 | 15 | Placebo |  |  |  | 15 |
| De Falco 2009 |  | Premenopausal | Symptomatic | Abdominal myomectomy | Triptorelin | Gonadotropin releasing hormone analogue | 3.75 mg subcutaneous monthly | 3 | 33 | No treatment |  |  |  | 29 |
| Donnez 2003 |  |  |  | Hysterectomy | Fulvestrant | Estrogen antagonist | 250 mg, 125 mg, 50 mg (3 different doses) intramuscular once every 4 weeks | 3 | 187 | Placebo |  |  |  | 60 |
| Engel 2007 | > 4 cm | Premenopausal |  | Hysterectomy or myomectomy | Cetrorelix | Gonadotropin releasing hormone antagonist | 10 mg once a week, 10 mg every two weeks, or 5 mg once a week (3 different doses) | 1 | 82 | Placebo |  |  |  | 27 |
| Engman 2009 |  | Premenopausal | Symptomatic | Uterine surgery (further details not available) | Mifepristone | Progesterone antagonist | 50 mg orally every alternate day | 3 | 14 | Placebo |  |  |  | 14 |
| Fedele 1990a |  |  | Symptomatic | Hysterectomy or myomectomy | Buserelin | Gonadotropin releasing hormone analogue | 400 mcg intransally thrice daily | 6 | 22 | No treatment |  |  |  | 20 |
| Fedele 1990b |  |  | Symptomatic | Myomectomy | Buserelin | Gonadotropin releasing hormone analogue | 1200 mcg/day intransally | 3 | 8 | No treatment |  |  |  | 16 |
| Fernandez-Mentoli 1995 |  | Premenopausal |  | Hysterectomy | Buserelin | Gonadotropin releasing hormone analogue | 900 mcg/day intransally | 3 | 20 | No treatment |  |  |  | 10 |
| Friedman 1989 | > 3 cm or uterine volume of at least 150 ml or > 12 weeks gestational size | Premenopausal | Symptomatic | Myomectomy | Leuprolide | Gonadotropin releasing hormone analogue | 3.75 mg intramuscular every 4 weeks | 4 | 9 | Placebo |  |  |  | 9 |
| Gerris 1996 |  | Premenopausal |  | Hysterectomy | Goserelin | Gonadotropin releasing hormone analogue | 3.6 mg subcutaneous monthly | 3 | 123 | No treatment |  |  |  | 124 |
| Golan 1993 | > 12 weeks gestation in size |  | Symptomatic | Hysterectomy or myomectomy | Triptorelin | Gonadotropin releasing hormone analogue | 3.2 mg subcutaneous monthly | 2 | 29 | No treatment |  |  |  | 24 |
| Hudecek 2012a |  |  |  | Laparoscopic myomectomy | Goserelin | Gonadotropin releasing hormone analogue | 3.6 mg subcutaneous every 4 weeks | 3 | 42 | No treatment |  |  |  | 48 |
| Hudecek 2012b |  |  |  | Open myomectomy | Goserelin | Gonadotropin releasing hormone analogue | 3.6 mg subcutaneous every 4 weeks | 3 | 78 | No treatment |  |  |  | 44 |
| Levens 2008 |  | Premenopausal | Symptomatic | Hysterectomy | Ulipristal | Selective progesterone receptor modulator | 20 mg/day and 10 mg/day (2 different doses) oral | 3 | 14 | Placebo |  |  |  | 6 |
| Lieto 2005 |  | Premenopausal | Symptomatic |  | Leuprolide | Gonadotropin releasing hormone analogue | 3.75 mg subcutaneous monthly | 4 | 23 | Tibolone+leuprolide | Estrogen agonist + Gonadotropin releasing hormone analogue | 2.5 mg/day oral + 3.75 mg subcutaneous monthly | 4 | 19 |
| Lim 2008 | > 14 weeks gestation in size | Premenopausal | Symptomatic | Abdominal hysterectomy | Goserelin | Gonadotropin releasing hormone analogue | 3.6 mg subcutaneous every 4 weeks | 3 | 34 | Leuprolide | Gonadotropin releasing hormone analogue | 3.75 mg subcutaneous every 4 weeks | 3 | 31 |
| Lumsden 1994 |  | Premenopausal | Symptomatic | Abdominal hysterectomy | Goserelin | Gonadotropin releasing hormone analogue | 3.6 mg injections (intramuscular or subcutaneous not stated) monthly | 3 | 35 | Placebo |  |  |  | 36 |
| Mavrelos 2010 |  |  |  | Hysteroscopic resection | Goserelin | Gonadotropin releasing hormone analogue | 3.6 mg subcutaneous every 4 weeks | 3 | 24 | Placebo |  |  |  | 23 |
| Melli 2007 | > 5 cm |  |  | Myomectomy | Triptorelin | Gonadotropin releasing hormone analogue | 3.75 mg injections (intramuscular or subcutaneous not stated) every 4 weeks | 4 | 25 | Cabergoline | Dopamine agonist | 0.5 mg once a week | 2 months | 25 |
| Muneyyirci-Delale 2007 | > 8 weeks gestation in size | Premenopausal | Symptomatic | Hysterectomy or myomectomy | Goserelin | Gonadotropin releasing hormone analogue | 10.8 mg injections (intramuscular or subcutaneous not stated) once | 3 | 34 | Placebo |  |  |  | 38 |
| Muzii 2010 | 1 cm to 3 cm | Premenopausal |  | Hysteroscopic resection | Triptorelin | Gonadotropin releasing hormone analogue | 3.75 mg intramuscular every 4 weeks | 2 | 20 | No treatment |  |  |  | 19 |
| Palomba 2002a | largest fibroid < 400 to 500 ml |  | Symptomatic | Laparoscopic myomectomy | Tibolone+Leuprolide | Estrogen agonist + Gonadotropin releasing hormone analogue | 2.5 mg oral daily + 3.75 mg intramuscular every 4 weeks | 2 | 20 | No treatment |  |  |  | 21 |
| Palomba 2005 | > 2 cm | Postmenopausal | Symptomatic | Hysterectomy or myomectomy | Raloxifene | Selective estrogen receptor modulator | 180 mg/day | 3 | 20 | Placebo |  |  |  | 19 |
| Reinsch 1994 |  |  |  | Hysterectomy or myomectomy | Mifepristone | Progesterone antagonist | 25 mg oral every alternate days | 3 | 8 | Leuprolide | Gonadotropin releasing hormone analogue | 3.75 mg intramuscular monthly | 3 | 6 |
| Rutgers 1995 |  |  |  | Hysterectomy or myomectomy | Leuprolide | Gonadotropin releasing hormone analogue | 7.5 mg or 3.75 mg intramuscular monthly (2 different doses) | 3 | 30 | Placebo |  |  |  | 16 |
| Seracchioli 2003 | > 16 weeks gestation < 20 weeks gestation |  | Symptomatic | Laparoscopic hysterectomy | Triptorelin | Gonadotropin releasing hormone analogue | 11.25 mg injections (intramuscular or subcutaneous not stated) once | 3 | 31 | No treatment |  |  |  | 31 |
| Shaw 1997 | > 8 weeks gestation in size |  | Symptomatic | Hysterectomy | Buserelin | Gonadotropin releasing hormone analogue | 3.6 mg injections (intramuscular or subcutaneous not stated) every 4 weeks | 3 | 103 | Placebo |  |  |  | 107 |
| Stovall 1991 | 14 to 18 weeks gestation in size |  | Symptomatic | Hysterectomy | Leuprolide | Gonadotropin releasing hormone analogue | 3.75 mg intramuscular every 4 weeks or 0.5 mg subcutaneous every day | 2 | 25 | No treatment |  |  |  | 25 |
| Stovall 1994a | 14 to 18 weeks gestation in size | Premenopausal | Symptomatic | Hysterectomy | Leuprolide | Gonadotropin releasing hormone analogue | 3.75 mg intramuscular every 4 weeks or 0.5 mg subcutaneous every day | 2 | 45 | No treatment |  |  |  | 45 |
| Stovall 1994b | > 18 weeks gestation in size | Premenopausal | Symptomatic | Hysterectomy | Leuprolide | Gonadotropin releasing hormone analogue | 3.75 mg intramuscular every 4 weeks or 0.5 mg subcutaneous every day | 2 | 30 | No treatment |  |  |  | 30 |
| Stovall 1995 |  | Premenopausal | Symptomatic | Hysterectomy or myomectomy | Leuprolide | Gonadotropin releasing hormone analogue | 7.5 mg or 3.75 mg intramuscular monthly (2 different doses) | 3 | 211 | Placebo |  |  |  | 98 |
| van den Ven 2001 |  |  |  | Hysterectomy or myomectomy | Triptorelin | Gonadotropin releasing hormone analogue | 3.75 mg intramuscular every 4 weeks | 4 | 21 | No treatment |  |  |  | 18 |
| Vercellini 2003 |  | Premenopausal | Symptomatic | Myomectomy | Triptorelin | Gonadotropin releasing hormone analogue | 3.75 mg intramuscular every 4 weeks | 2 | 49 | No treatment |  |  |  | 48 |
| Verspyck 2000 | > 5 cm | Premenopausal | Symptomatic | Uterine surgery (further details not available) | Leuprolide | Gonadotropin releasing hormone analogue | 3.75 mg intramuscular every 4 weeks | 4 | 33 | Lynesterol | Progesterone | 10 mg/day | 4 | 23 |
| Wilkens 2008 | > 2 cm | Premenopausal | Symptomatic | Hysterectomy | Asoprisnil | Selective progesterone receptor modulator | 25 mg/day or 10 mg/day (2 different doses) | 3 | 23 | Placebo |  |  |  | 10 |
| Zullo 1997 | > 400 ml < 500 ml | Premenopausal | Symptomatic | Laparoscopic myomectomy | Leuprolide | Gonadotropin releasing hormone analogue | 3.75 mg intramuscular every 4 weeks | 2 | 35 | No treatment |  |  |  | 32 |

## Table 12: Medical treatment prior to planned surgery – risk of bias

| Study name | Random sequence generation | Allocation concealment | Blinding of patients and healthcare providers | Blinding of outcome assessors | Missing outcome bias | Selective outcome reporting | Source of funding | Overall risk of bias |
| --- | --- | --- | --- | --- | --- | --- | --- | --- |
| Audebert 1994 | ? | ? | ? | ? | - | - | ? | Unclear or high |
| Balasch 1995 | ? | ? | ? | ? | ? | - | ? | Unclear or high |
| Barbieri 1993 | ? | ? | ? | ? | - | - | - | Unclear or high |
| Baytur 2007 | ? | + | ? | ? | ? | - | ? | Unclear or high |
| Bustos-Lopez 1995 | ? | ? | ? | ? | ? | - | ? | Unclear or high |
| Campo 1999 | + | ? | ? | ? | ? | - | ? | Unclear or high |
| Coddington 2009 | ? | ? | ? | ? | + | - | - | Unclear or high |
| D'Anna 1994 | ? | ? | ? | ? | ? | - | ? | Unclear or high |
| De Falco 2009 | ? | ? | ? | ? | ? | - | ? | Unclear or high |
| Donnez 2003 | ? | ? | - | - | + | - | ? | Unclear or high |
| Engel 2007 | ? | ? | + | + | + | - | - | Unclear or high |
| Engman 2009 | + | + | + | + | - | - | + | Unclear or high |
| Fedele 1990a | ? | ? | ? | ? | ? | - | ? | Unclear or high |
| Fedele 1990b | ? | ? | ? | ? | ? | - | ? | Unclear or high |
| Fernandez-Mentoli 1995 | ? | ? | ? | ? | ? | - | + | Unclear or high |
| Friedman 1989 | ? | ? | ? | ? | ? | - | - | Unclear or high |
| Gerris 1996 | ? | ? | ? | ? | - | - | - | Unclear or high |
| Golan 1993 | ? | ? | ? | ? | ? | - | ? | Unclear or high |
| Hudecek 2012a | ? | ? | ? | ? | ? | - | ? | Unclear or high |
| Levens 2008 | + | + | + | + | - | - | - | Unclear or high |
| Lieto 2005 | ? | ? | ? | ? | - | - | ? | Unclear or high |
| Lim 2008 | ? | ? | ? | ? | - | + | ? | Unclear or high |
| Lumsden 1994 | + | + | + | + | + | + | + | Low |
| Mavrelos 2010 | + | + | + | + | + | - | + | Unclear or high |
| Melli 2007 | ? | ? | ? | ? | ? | - | ? | Unclear or high |
| Muneyyirci-Delale 2007 | ? | + | + | + | - | - | - | Unclear or high |
| Muzii 2010 | + | + | ? | ? | + | - | ? | Unclear or high |
| Palomba 2002a | + | + | - | + | - | + | + | Unclear or high |
| Palomba 2005 | + | + | + | + | - | + | + | Unclear or high |
| Reinsch 1994 | ? | ? | ? | ? | ? | - | ? | Unclear or high |
| Rutgers 1995 | + | ? | ? | ? | ? | - | - | Unclear or high |
| Seracchioli 2003 | ? | ? | ? | ? | ? | - | ? | Unclear or high |
| Shaw 1997 | ? | ? | ? | ? | ? | - | - | Unclear or high |
| Stovall 1991 | ? | ? | ? | ? | ? | - | - | Unclear or high |
| Stovall 1994a | + | ? | ? | ? | ? | - | - | Unclear or high |
| Stovall 1995 | ? | ? | ? | ? | + | - | - | Unclear or high |
| van den Ven 2001 | ? | ? | ? | ? | ? | - | ? | Unclear or high |
| Vercellini 2003 | + | + | - | ? | - | - | - | Unclear or high |
| Verspyck 2000 | ? | ? | - | ? | + | - | ? | Unclear or high |
| Wilkens 2008 | + | + | + | + | + | + | - | Unclear or high |
| Zullo 1997 | + | ? | ? | ? | - | - | ? | Unclear or high |

## Table 13: Medical treatment prior to planned surgery – proportion of adverse events – summary of findings table

| **Adverse events proportion (5 trials; 477 participants)** | | |
| --- | --- | --- |
| Overall quality of evidence | Very low |  |
| **Groups** | **Illustrative risk** | **Treatment effect** |
| Assumed risk in control group (Placebo) | 360 per 1000 | - |
| Corresponding risk in Asoprisnil | 589 per 1000 (36 to 982) | OR 2.54; 95% CrI 0.07 to 97.06 |
| Corresponding risk in Fulvestrant | 334 per 1000 (210 to 485) | OR 0.89; 95% CrI 0.47 to 1.68 |
| Corresponding risk in Goserelin | 781 per 1000 (652 to 872) | OR 6.35; 95% CrI 3.33 to 12.1 |
| Corresponding risk in Raloxifene | 605 per 1000 (67 to 971) | OR 2.73; 95% CrI 0.13 to 58.58 |
| Corresponding risk in Ulipristal | 610 per 1000 (37 to 984) | OR 2.78; 95% CrI 0.07 to 111.79 |

## Table 14: Medical treatment prior to planned surgery – proportion of adverse events – pairwise comparisons

|  | **Placebo** | **Asoprisnil** | **Fulvestrant** | **Goserelin** | **Raloxifene** | **Ulipristal** |
| --- | --- | --- | --- | --- | --- | --- |
| **Placebo** | - | OR 2.54; 95% CrI 0.07 to 97.06 | OR 0.89; 95% CrI 0.47 to 1.68 | OR 6.35; 95% CrI 3.33 to 12.1 | OR 2.73; 95% CrI 0.13 to 58.58 | OR 2.78; 95% CrI 0.07 to 111.79 |
| **Asoprisnil** | - | - | OR 0.35; 95% CrI 0.01 to 14.11 | OR 2.5; 95% CrI 0.06 to 100.75 | OR 1.07; 95% CrI 0.01 to 125.3 | OR 1.09; 95% CrI 0.01 to 195.58 |
| **Fulvestrant** | - | - | - | OR 7.13; 95% CrI 2.89 to 17.59 | OR 3.06; 95% CrI 0.13 to 70.17 | OR 3.12; 95% CrI 0.07 to 132.46 |
| **Goserelin** | - | - | - | - | OR 0.43; 95% CrI 0.02 to 9.87 | OR 0.44; 95% CrI 0.01 to 18.62 |
| **Raloxifene** | - | - | - | - | - | OR 1.02; 95% CrI 0.01 to 124.09 |
| **Ulipristal** | - | - | - | - | - | - |

Tau = 0.003

## Table 15: Medical treatment prior to planned surgery – number of adverse events – summary of findings table

| **Adverse events (number) (8 trials; 686 participants)** | | |
| --- | --- | --- |
| Overall quality of evidence: Very low | | |
| **Groups** | **Illustrative risk** | **Treatment effect** |
| Assumed risk in control group (No active treatment) | Check the control group proportion | - |
| Corresponding risk in Goserelin | 1000 per 1000 (1000 to 1000) | OR 1.66; 95% CrI 1.33 to 2.06 |
| Corresponding risk in Leuprolide | 1000 per 1000 (1000 to 1000) | OR 1.38; 95% CrI 1.17 to 1.62 |
| Corresponding risk in Lynesterol | 1000 per 1000 (1000 to 1000) | OR 0.78; 95% CrI 0.45 to 1.37 |
| Corresponding risk in Raloxifene | 1000 per 1000 (0 to 1000) | OR 0.59; 95% CrI 0.22 to 1.59 |
| Corresponding risk in Tibolone+Leuprolide | 1000 per 1000 (0 to 1000) | OR 0.68; 95% CrI 0.11 to 3.99 |
| Corresponding risk in Ulipristal | 1000 per 1000 (1000 to 1000) | OR 1.31; 95% CrI 0.77 to 2.21 |

## Table 16: Medical treatment prior to planned surgery – number of adverse events – pairwise comparisons

|  | **No active treatment** | **Goserelin** | **Leuprolide** | **Lynesterol** | **Raloxifene** | **Tibolone+Leuprolide** | **Ulipristal** |
| --- | --- | --- | --- | --- | --- | --- | --- |
| **No active treatment** | - | OR 1.66; 95% CrI 1.33 to 2.06 | OR 1.38; 95% CrI 1.17 to 1.62 | OR 0.78; 95% CrI 0.45 to 1.37 | OR 0.59; 95% CrI 0.22 to 1.59 | OR 0.68; 95% CrI 0.11 to 3.99 | OR 1.31; 95% CrI 0.77 to 2.21 |
| **Goserelin** | - | - | OR 0.83; 95% CrI 0.63 to 1.09 | OR 0.47; 95% CrI 0.26 to 0.86 | OR 0.36; 95% CrI 0.13 to 0.98 | OR 0.41; 95% CrI 0.07 to 2.44 | OR 0.79; 95% CrI 0.45 to 1.39 |
| **Leuprolide** | - | - | - | OR 0.57; 95% CrI 0.32 to 1.02 | OR 0.43; 95% CrI 0.16 to 1.17 | OR 0.49; 95% CrI 0.08 to 2.92 | OR 0.95; 95% CrI 0.55 to 1.65 |
| **Lynesterol** | - | - | - | - | OR 0.75; 95% CrI 0.24 to 2.35 | OR 0.87; 95% CrI 0.13 to 5.57 | OR 1.67; 95% CrI 0.78 to 3.6 |
| **Raloxifene** | - | - | - | - | - | OR 1.15; 95% CrI 0.15 to 8.79 | OR 2.22; 95% CrI 0.72 to 6.83 |
| **Tibolone+Leuprolide** | - | - | - | - | - | - | OR 1.93; 95% CrI 0.3 to 12.29 |
| **Ulipristal** | - | - | - | - | - | - | - |

# Tau = 0.008

## Table 17: Medical treatment prior to planned surgery – proportion undergoing abdominal hysterecctomy – summary of findings table

| **Abdominal hysterectomy (11 trials; 549 participants)** | | |
| --- | --- | --- |
| Overall quality of evidence: Very low | | |
| **Groups** | **Illustrative risk** | **Treatment effect** |
| Assumed risk in control group (No active treatment) | 800 per 1000 | - |
| Corresponding risk in Asoprisnil | 809 per 1000 (661 to 902) | OR 1.06; 95% CrI 0.49 to 2.3 |
| Corresponding risk in Goserelin | 750 per 1000 (672 to 814) | OR 0.75; 95% CrI 0.51 to 1.09 |
| Corresponding risk in Leuprolide | 686 per 1000 (614 to 750) | OR 0.55; 95% CrI 0.4 to 0.75 |
| Corresponding risk in Lynesterol | 650 per 1000 (438 to 815) | OR 0.46; 95% CrI 0.2 to 1.1 |
| Corresponding risk in Raloxifene | 0 per 1000 (0 to 1000) | OR 0; 95% CrI 0 to 3.92811731858738E+51 |
| Corresponding risk in Triptorelin | 785 per 1000 (703 to 849) | OR 0.91; 95% CrI 0.59 to 1.4 |

## Table 18: Medical treatment prior to planned surgery – proportion undergoing abdominal hysterectomy – pairwise comparisons

|  | **No active treatment** | **Asoprisnil** | **Goserelin** | **Leuprolide** | **Lynesterol** | **Raloxifene** | **Triptorelin** |
| --- | --- | --- | --- | --- | --- | --- | --- |
| **No active treatment** | - | OR 1.06; 95% CrI 0.49 to 2.3 | OR 0.75; 95% CrI 0.51 to 1.09 | OR 0.55; 95% CrI 0.4 to 0.75 | OR 0.46; 95% CrI 0.2 to 1.1 | OR 0; 95% CrI 0 to 3.92811731858738E+51 | OR 0.91; 95% CrI 0.59 to 1.4 |
| **Asoprisnil** | - | - | OR 0.71; 95% CrI 0.3 to 1.68 | OR 0.52; 95% CrI 0.22 to 1.19 | OR 0.44; 95% CrI 0.14 to 1.4 | OR 0; 95% CrI 0 to 3.71816862344198E+51 | OR 0.86; 95% CrI 0.35 to 2.09 |
| **Goserelin** | - | - | - | OR 0.73; 95% CrI 0.45 to 1.19 | OR 0.62; 95% CrI 0.24 to 1.59 | OR 0; 95% CrI 0 to 5.24993208036429E+51 | OR 1.22; 95% CrI 0.69 to 2.16 |
| **Leuprolide** | - | - | - | - | OR 0.85; 95% CrI 0.34 to 2.13 | OR 0; 95% CrI 0 to 7.20142821099523E+51 | OR 1.67; 95% CrI 0.98 to 2.85 |
| **Lynesterol** | - | - | - | - | - | OR 0; 95% CrI 0 to 8.49644666503189E+51 | OR 1.97; 95% CrI 0.75 to 5.18 |
| **Raloxifene** | - | - | - | - | - | - | OR 29059736919475800; 95% CrI 0 to 3.63851105996076E+84 |
| **Triptorelin** | - | - | - | - | - | - | - |

Tau = 0.0095

## Table 19: Medical treatment prior to planned surgery – proportion undergoing blood transfusion – summary of findings table

| **Blood transfusion proportion (18 trials; 1446 participants)** | | |
| --- | --- | --- |
| Overall quality of evidence: Very low | | |
| **Groups** | **Illustrative risk** | **Treatment effect** |
| Assumed risk in control group (No active treatment) | 110 per 1000 | - |
| Corresponding risk in Buserelin | 0 per 1000 (0 to 1000) | OR 0; 95% CrI 0 to 2958917834655950 |
| Corresponding risk in Goserelin | 48 per 1000 (26 to 85) | OR 0.40; 95% CrI 0.22 to 0.75 |
| Corresponding risk in Leuprolide | 45 per 1000 (25 to 81) | OR 0.38; 95% CrI 0.2 to 0.71 |
| Corresponding risk in Lynesterol | 227 per 1000 (18 to 828) | OR 2.38; 95% CrI 0.15 to 38.82 |
| Corresponding risk in Tibolone+Leuprolide | 0 per 1000 (0 to 1000) | OR 0; 95% CrI 0 to 79140409086166400 |
| Corresponding risk in Triptorelin | 61 per 1000 (24 to 147) | OR 0.53; 95% CrI 0.2 to 1.39 |

## Table 20: Medical treatment prior to planned surgery – proportion undergoing blood transfusion – pairwise comparisons

|  | **No active treatment** | **Buserelin** | **Goserelin** | **Leuprolide** | **Lynesterol** | **Tibolone+Leuprolide** | **Triptorelin** |
| --- | --- | --- | --- | --- | --- | --- | --- |
| **No active treatment** | - | OR 0; 95% CrI 0 to 2958917834655950 | OR 0.4; 95% CrI 0.22 to 0.75 | OR 0.38; 95% CrI 0.2 to 0.71 | OR 2.38; 95% CrI 0.15 to 38.82 | OR 0; 95% CrI 0 to 79140409086166400 | OR 0.53; 95% CrI 0.2 to 1.39 |
| **Buserelin** | - | - | OR 8.28881352938031E+34; 95% CrI 0 to 5.04461270992421E+85 | OR 7.83191332353391E+34; 95% CrI 0 to 4.76657083616563E+85 | OR 4.88134963027041E+35; 95% CrI 0 to 3.06648862955365E+86 | OR 5.16; 95% CrI 0 to 1.09266644619992E+73 | OR 1.0826608328165E+35; 95% CrI 0 to 6.60488691385415E+85 |
| **Goserelin** | - | - | - | OR 0.94; 95% CrI 0.39 to 2.28 | OR 5.89; 95% CrI 0.34 to 102.98 | OR 0; 95% CrI 0 to 196380564440088000 | OR 1.31; 95% CrI 0.41 to 4.14 |
| **Leuprolide** | - | - | - | - | OR 6.23; 95% CrI 0.36 to 109.01 | OR 0; 95% CrI 0 to 207838348191431000 | OR 1.38; 95% CrI 0.44 to 4.39 |
| **Lynesterol** | - | - | - | - | - | OR 0; 95% CrI 0 to 34405340963891800 | OR 0.22; 95% CrI 0.01 to 4.27 |
| **Tibolone+Leuprolide** | - | - | - | - | - | - | OR 2.10014594136437E+34; 95% CrI 0 to 6.64692065545494E+85 |
| **Triptorelin** | - | - | - | - | - | - | - |

Tau = 0.013

## Table 21: Medical treatment prior to planned surgery – hospital stay – summary of findings table

| **Hospital stay (14 trials; 1109 participants)** | | |
| --- | --- | --- |
| Overall quality of evidence: Very low | | |
| **Groups** | **Illustrative means (in days)** | **Treatment effect** |
| Assumed mean in control group (No active treatment) | 5.7 | - |
| Mean in Goserelin | 0.02 lower (1.1 lower to 1.06 higher) | MD -0.02; 95% CrI -1.1 to 1.06 |
| Mean in Leuprolide | 0.91 lower (1.95 lower to 0.14 higher) | MD -0.91; 95% CrI -1.95 to 0.14 |
| Mean in Triptorelin | 0.06 higher (1.16 lower to 1.27 higher) | MD 0.06; 95% CrI -1.16 to 1.27 |

## Table 22: Medical treatment prior to planned surgery – hospital stay – pairwise comparisons

|  | **No active treatment** | **Goserelin** | **Leuprolide** | **Triptorelin** |
| --- | --- | --- | --- | --- |
| **No active treatment** | - | MD -0.02; 95% CrI -1.1 to 1.06 | MD -0.91; 95% CrI -1.95 to 0.14 | MD 0.06; 95% CrI -1.16 to 1.27 |
| **Goserelin** | - | - | MD -0.88; 95% CrI -2.38 to 0.62 | MD 0.08; 95% CrI -1.54 to 1.7 |
| **Leuprolide** | - | - | - | MD 0.96; 95% CrI -0.64 to 2.56 |
| **Triptorelin** | - | - | - | - |

Tau = 0.90

## Table 23: Medical treatment prior to planned surgery – operating time – summary of findings table

| **Operating time (21 trials; 1401 participants)** | | |
| --- | --- | --- |
| Overall quality of evidence: Very low | | |
| **Groups** | **Illustrative means (in minutes)** | **Treatment effect** |
| Assumed mean in control group (No active treatment) | 85 | - |
| Mean in Goserelin | 1.38 lower (8.65 lower to 5.89 higher) | MD -1.38; 95% CrI -8.65 to 5.89 |
| Mean in Leuprolide | 8.56 lower (15.28 lower to 1.84 lower) | MD -8.56; 95% CrI -15.28 to -1.84 |
| Mean in Tibolone+Leuprolide | 9.26 lower (23.08 lower to 4.57 higher) | MD -9.26; 95% CrI -23.08 to 4.57 |
| Mean in Triptorelin | 0.25 higher (6.25 lower to 6.75 higher) | MD 0.25; 95% CrI -6.25 to 6.75 |
| Mean in Buserelin | 19.88 higher (73.3 lower to 113.06 higher) | MD 19.88; 95% CrI -73.30 to 113.06 |

## Table 24: Medical treatment prior to planned surgery – operating time – pairwise comparisons

|  | **No active treatment** | **Goserelin** | **Leuprolide** | **Tibolone+Leuprolide** | **Triptorelin** | **Buserelin** |
| --- | --- | --- | --- | --- | --- | --- |
| **No active treatment** | - | MD -1.38; 95% CrI -8.65 to 5.89 | MD -8.56; 95% CrI -15.28 to -1.84 | MD -9.26; 95% CrI -23.08 to 4.57 | MD 0.25; 95% CrI -6.25 to 6.75 | MD 19.88; 95% CrI -73.3 to 113.06 |
| **Goserelin** | - | - | MD -7.18; 95% CrI -17.09 to 2.72 | MD -7.88; 95% CrI -23.5 to 7.74 | MD 1.63; 95% CrI -8.12 to 11.39 | MD 21.26; 95% CrI -72.2 to 114.72 |
| **Leuprolide** | - | - | - | MD -0.69; 95% CrI -16.06 to 14.68 | MD 8.82; 95% CrI -0.53 to 18.17 | MD 28.44; 95% CrI -64.98 to 121.86 |
| **Tibolone+Leuprolide** | - | - | - | - | MD 9.51; 95% CrI -5.77 to 24.78 | MD 29.14; 95% CrI -65.06 to 123.33 |
| **Triptorelin** | - | - | - | - | - | MD 19.63; 95% CrI -73.78 to 113.03 |
| **Buserelin** | - | - | - | - | - | - |

Tau = 4.39

## Table 25: Medical treatment prior to planned surgery – haemoglobin – summary of findings table

| **Haemoglobin (8 trials; 482 participants)** | | |
| --- | --- | --- |
| Overall quality of evidence: Very low | | |
| **Groups** | **Illustrative means (in gm/dl)** | **Treatment effect** |
| Assumed mean in control group (No active treatment) | 11 | - |
| Mean in Goserelin | 0.42 higher (0.08 lower to 0.92 higher) | MD 0.42; 95% CrI -0.08 to 0.92 |
| Mean in Leuprolide | 1.28 higher (0.93 higher to 1.63 higher) | MD 1.28; 95% CrI 0.93 to 1.63 |
| Mean in Mifepristone | 1.10 higher (0.04 higher to 2.15 higher) | MD 1.10; 95% CrI 0.04 to 2.15 |
| Mean in Tibolone+Leuprolide | 1.16 higher (0.65 higher to 1.66 higher) | MD 1.16; 95% CrI 0.65 to 1.66 |
| Mean in Triptorelin | 0.40 higher (0.08 lower to 0.89 higher) | MD 0.40; 95% CrI -0.08 to 0.89 |

## Table 26: Medical treatment prior to planned surgery – haemoglobin – pairwise comparisons

|  | **No active treatment** | **Goserelin** | **Leuprolide** | **Mifepristone** | **Tibolone+Leuprolide** | **Triptorelin** |
| --- | --- | --- | --- | --- | --- | --- |
| **No active treatment** | - | MD 0.42; 95% CrI -0.08 to 0.92 | MD 1.28; 95% CrI 0.93 to 1.63 | MD 1.1; 95% CrI 0.04 to 2.15 | MD 1.16; 95% CrI 0.65 to 1.66 | MD 0.4; 95% CrI -0.08 to 0.89 |
| **Goserelin** | - | - | MD 0.86; 95% CrI 0.24 to 1.47 | MD 0.68; 95% CrI -0.5 to 1.85 | MD 0.74; 95% CrI 0.02 to 1.45 | MD -0.02; 95% CrI -0.72 to 0.68 |
| **Leuprolide** | - | - | - | MD -0.18; 95% CrI -1.29 to 0.93 | MD -0.12; 95% CrI -0.73 to 0.49 | MD -0.88; 95% CrI -1.47 to -0.28 |
| **Mifepristone** | - | - | - | - | MD 0.06; 95% CrI -1.11 to 1.23 | MD -0.7; 95% CrI -1.86 to 0.47 |
| **Tibolone+Leuprolide** | - | - | - | - | - | MD -0.76; 95% CrI -1.46 to -0.06 |
| **Triptorelin** | - | - | - | - | - | - |

Tau = 0.005

1. Bagaria M, Suneja A, Vaid NB, Guleria K, Mishra K. Low-dose mifepristone in treatment of uterine leiomyoma: A randomised double-blind placebo-controlled clinical trial. Aust N Z J Obstet Gynaecol. 2009;49(1):77-83. doi: 10.1111/j.1479-828X.2008.00931.x. PubMed PMID: WOS:000263855900016.

2. Esteve JL, Acosta R, Perez Y, Rodriguez B, Seigler I, Sanchez C, et al. Mifepristone versus placebo to treat uterine myoma: a double-blind, randomized clinical trial. International journal of women's health. 2013;5:361-9. Epub 2013/07/12. doi: 10.2147/ijwh.s42770. PubMed PMID: 23843709; PubMed Central PMCID: PMCPmc3702243.

3. Chwalisz K, Larsen L, Mattia-Goldberg C, Edmonds A, Elger W, Winkel CA. A randomized, controlled trial of asoprisnil, a novel selective progesterone receptor modulator, in women with uterine leiomyomata. Fertility and sterility. 2007;87(6):1399-412. doi: 10.1016/j.fertnstert.2006.11.094. PubMed PMID: WOS:000247150100024.

4. Constantini S, Anserini P, Valenzano M, Remorgida V, Venturini PL, De Cecco L. Luteinizing hormone-releasing hormone analog therapy of uterine fibroid: Analysis of results obtained with buserelin administered intranasally and goserelin administered subcutaneously as a monthly depot. European Journal of Obstetrics Gynecology and Reproductive Biology. 1990;37(1):63-9. PubMed PMID: 1990206478.

5. Daniels A, Pike M, Daniels J, Spicer D. Treatment with the GnRH agonist (GnRHa) deslorelin (D) and low-dose add-back estradiol (E2) is effective in reducing pain, bleeding and uterine volume(UV) while maintaining bone mineral density (BMD) in women with symptomatic uterine fibroids (UF). Fertility & Sterility. 2002;Vol 78(3 Suppl 1):S65-6, Abstract no: O-170. PubMed PMID: CN-00404980.

6. De Aloysio D, Altieri P, Penacchioni P, Salgarello M, Ventura V. Bleeding patterns in recent postmenopausal outpatients with uterine myomas: Comparison between two regimens of HRT. Maturitas. 1998;29(3):261-4. doi: <http://dx.doi.org/10.1016/S0378-5122%2898%2900014-0>. PubMed PMID: 1998237358.

7. Donnez J, Tatarchuk TF, Bouchard P, Puscasiu L, Zakharenko NF, Ivanova T, et al. Ulipristal acetate versus placebo for fibroid treatment before surgery. The New England journal of medicine. 2012;366(5):409-20. doi: 10.1056/NEJMoa1103182. PubMed PMID: CN-00804100.

8. Donnez J, Tomaszewski J, Vazquez F, Bouchard P, Lemieszczuk B, Baro F, et al. Ulipristal acetate versus leuprolide acetate for uterine fibroids. New England Journal of Medicine. 2012;366(5):421-32. doi: <http://dx.doi.org/10.1056/NEJMoa1103180>. PubMed PMID: 2012072934.

9. Fedele L, Bianchi S, Raffaelli R, Zanconato G. A randomized study of the effects of tibolone and transdermal estrogen replacement therapy in postmenopausal women with uterine myomas. European journal of obstetrics, gynecology, and reproductive biology. 2000;88(1):91-4. PubMed PMID: CN-00274902.

10. Fiscella K, Eisinger SH, Meldrum S, Feng C, Fisher SG, Guzick DS. Effect of mifepristone for symptomatic leiomyomata on quality of life and uterine size: a randomized controlled trial. Obstetrics and gynecology. 2006;108(6):1381-7. doi: 10.1097/01.AOG.0000243776.23391.7b. PubMed PMID: CN-00574102.

11. Friedman AJ, Barbieri RL, Doubilet PM, Fine C, Schiff I. A randomized, double-blind trial of a gonadotropin releasing-hormone agonist (leuprolide) with or without medroxyprogesterone acetate in the treatment of leiomyomata uteri. Fertility and sterility. 1988;49(3):404-9. PubMed PMID: WOS:A1988M343000004.

12. Friedman AJ, Hoffman DI, Comite F, Browneller RW, Miller JD. Treatment of leiomyomata uteri with leuprolide acetate depot: a double-blind, placebo-controlled, multicenter study. The Leuprolide Study Group. Obstetrics and gynecology. 1991;77(5):720-5. PubMed PMID: CN-00074529.

13. Green LJ, Levy G, Wesley R, Nieman L, Armstrong A. Efficacyof ulipristal acetate forthe treatment of symptomatic uterine leiomyomas in African Americans. Fertility and sterility. 2012;1):S96. doi: <http://dx.doi.org/10.1016/j.fertnstert.2012.07.351>. PubMed PMID: 70871922.

14. Gregoriou O, Vitoratos N, Papadias C, Konidaris S, Costomenos D, Chryssikopoulos A. Effect of tibolone on postmenopausal women with myomas. Maturitas. 1997;27(2):187-91. doi: 10.1016/s0378-5122(97)00036-4. PubMed PMID: WOS:A1997XL50900012.

15. Jirecek S, Lee A, Pavo I, Crans G, Eppel W, Wenzl R. Raloxifene prevents the growth of uterine leiomyomas in premenopausal women. Fertility and sterility. 2004;81(1):132-6. Epub 2004/01/09. PubMed PMID: 14711556.

16. Levy G, Avila N, Armstrong AY, Nieman L. Does the selective progesterone receptor modulator ulipristal normalize the uterine cavity in women with leiomyoma. Reproductive Sciences. 2011;1):95A. doi: <http://dx.doi.org/10.1177/193371912011183s067>. PubMed PMID: 70491653.

17. Morris EP, Rymer J, Robinson J, Fogelman I. Efficacy of tibolone as "add-back therapy" in conjunction with a gonadotropin-releasing hormone analogue in the treatment of uterine fibroids. Fertility and sterility. 2008;89(2):421-8. doi: 10.1016/j.fertnstert.2007.02.064. PubMed PMID: WOS:000253246100024.

18. Nieman LK, Blocker W, Nansel T, Mahoney S, Reynolds J, Blithe D, et al. Efficacy and tolerability of CDB-2914 treatment for symptomatic uterine fibroids: a randomized, double-blind, placebo-controlled, phase IIb study. Fertility and sterility. 2011;95(2):767-U9. doi: 10.1016/j.fertnstert.2010.09.059. PubMed PMID: WOS:000286419000071.

19. Orsini G, Pinto V, Biase S, D'Altorio C, Lanzilotti G. Effects of hormone replacement therapy on postmenopausal women with uterine fibroids. Minerva Ginecologica. 1999;51(11):421-5. PubMed PMID: CN-00295054.

20. Palomba S, Affinito P, Tommaselli GA, Nappi C. A clinical trial of the effects of tibolone administered with gonadotropin-releasing hormone analogues for the treatment of uterine leiomyomata. Fertility and sterility. 1998;70(1):111-8. doi: 10.1016/s0015-0282(98)00128-9. PubMed PMID: WOS:000074479600020.

21. Palomba S, Orio F, Jr., Morelli M, Russo T, Pellicano M, Nappi C, et al. Raloxifene administration in women treated with gonadotropin-releasing hormone agonist for uterine leiomyomas: effects on bone metabolism. The Journal of clinical endocrinology and metabolism. 2002;87(10):4476-81. Epub 2002/10/05. PubMed PMID: 12364422.

22. Palomba S, Orio Jr F, Morelli M, Russo T, Pellicano M, Zupi E, et al. Raloxifene administration in premenopausal women with uterine leiomyomas: A pilot study. Journal of Clinical Endocrinology and Metabolism. 2002;87(8):3603-8. doi: <http://dx.doi.org/10.1210/jc.87.8.3603>. PubMed PMID: 2002288881.

23. Palomba S, Orio Jr F, Falbo A, Oppedisano R, Tolino A, Zullo F. Tibolone reverses the cognitive effects caused by leuprolide acetate administration, improving mood and quality of life in patients with symptomatic uterine leiomyomas. Fertility and sterility. 2008;90(1):165-73. doi: <http://dx.doi.org/10.1016/j.fertnstert.2007.05.061>. PubMed PMID: 2008307526.

24. Parsanezhad ME, Azmoon M, Alborzi S, Rajaeefard A, Zarei A, Kazerooni T, et al. A randomized, controlled clinical trial comparing the effects of aromatase inhibitor (letrozole) and gonadotropin-releasing hormone agonist (triptorelin) on uterine leiomyoma volume and hormonal status. Fertility and sterility. 2010;93(1):192-8. doi: 10.1016/j.fertnstert.2008.09.064. PubMed PMID: WOS:000273601200029.

25. Polatti F, Viazzo F, Colleoni R, Nappi RE. Uterine myoma in postmenopause: a comparison between two therapeutic schedules of HRT. Maturitas. 2000;37(1):27-32. doi: 10.1016/s0378-5122(00)00159-6. PubMed PMID: WOS:000165650900003.

26. Roshdy E, Rajaratnam V, Maitra S, Sabry M, Ait Allah AS, Al-Hendy A. Treatment of symptomatic Uterine fibroids with green tea extract: A pilot randomized controlled clinical study. International Journal of Women's Health. 2013;5(1):477-86. doi: <http://dx.doi.org/10.2147/IJWH.S41021>. PubMed PMID: 2013496484.

27. Sadan O, Ginath S, Sofer D, Rotmensch S, Debby A, Glezerman M, et al. The role of tamoxifen in the treatment of symptomatic uterine leiomyomata -- a pilot study. European journal of obstetrics, gynecology, and reproductive biology. 2001;96(2):183-6. PubMed PMID: CN-00348348.

28. Sayed GH, Zakherah MS, El-Nashar SA, Shaaban MM. A randomized clinical trial of a levonorgestrel-releasing intrauterine system and a low-dose combined oral contraceptive for fibroid-related menorrhagia. Int J Gynecol Obstet. 2011;112(2):126-30. doi: 10.1016/j.ijgo.2010.08.009. PubMed PMID: WOS:000286704100011.

29. Sayyah-Melli M, Tehrani-Gadim S, Dastranj-Tabrizi A, Gatrehsamani F, Morteza G, Ouladesahebmadarek E, et al. Comparison of the effect of gonadotropin-releasing hormone agonist and dopamine receptor agonist on uterine myoma growth Histologic, sonographic, and intra-operative changes. Saudi Med J. 2009;30(8):1024-33. PubMed PMID: WOS:000270844200007.

30. Schlaff WD, Zerhouni EA, Huth JA, Chen J, Damewood MD, Rock JA. A placebo-controlled trial of a depot gonadotropin-releasing hormone analogue (leuprolide) in the treatment of uterine leiomyomata. Obstetrics and gynecology. 1989;74(6):856-62. PubMed PMID: CN-00063914.

31. Simsek T, Karakus C, Trak B. Impact of different hormone replacement therapy regimens on the size of myoma uteri in postmenopausal period: tibolone versus transdermal hormonal replacement system. Maturitas. 2002;42(3):243-6. PubMed PMID: CN-00405090.

32. Varun N, Kumar A, Prasad S. Effect of low dose mifepristone on uterine leiomyoma in reproductive age group. Fertility and sterility. 2013;1):S78. doi: <http://dx.doi.org/10.1016/j.fertnstert.2013.07.1924>. PubMed PMID: 71164058.
