# Supplementary figures and images for: Medical Therapies for Uterine Fibroids – A Systematic Review and Network Meta-Analysis of Randomised Controlled Trials
Source: PLoS One. 2016 Feb 26;11(2):e0149631. doi: 10.1371/journal.pone.0149631 (PMC4769153; doi:10.1371/journal.pone.0149631)

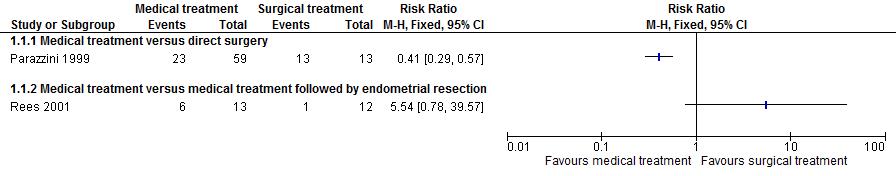

Supplement: S1 Fig — (JPG) [file pone.0149631.s004.jpg]

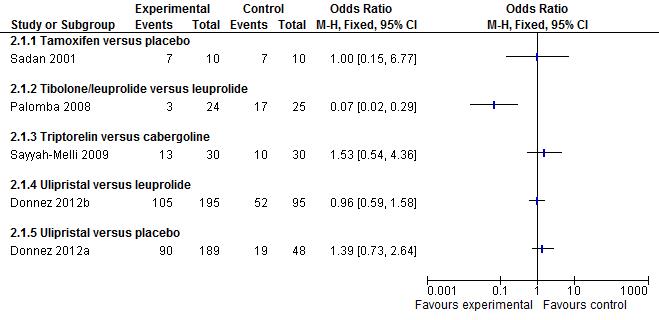

Supplement: S2 Fig — (JPG) [file pone.0149631.s005.jpg]

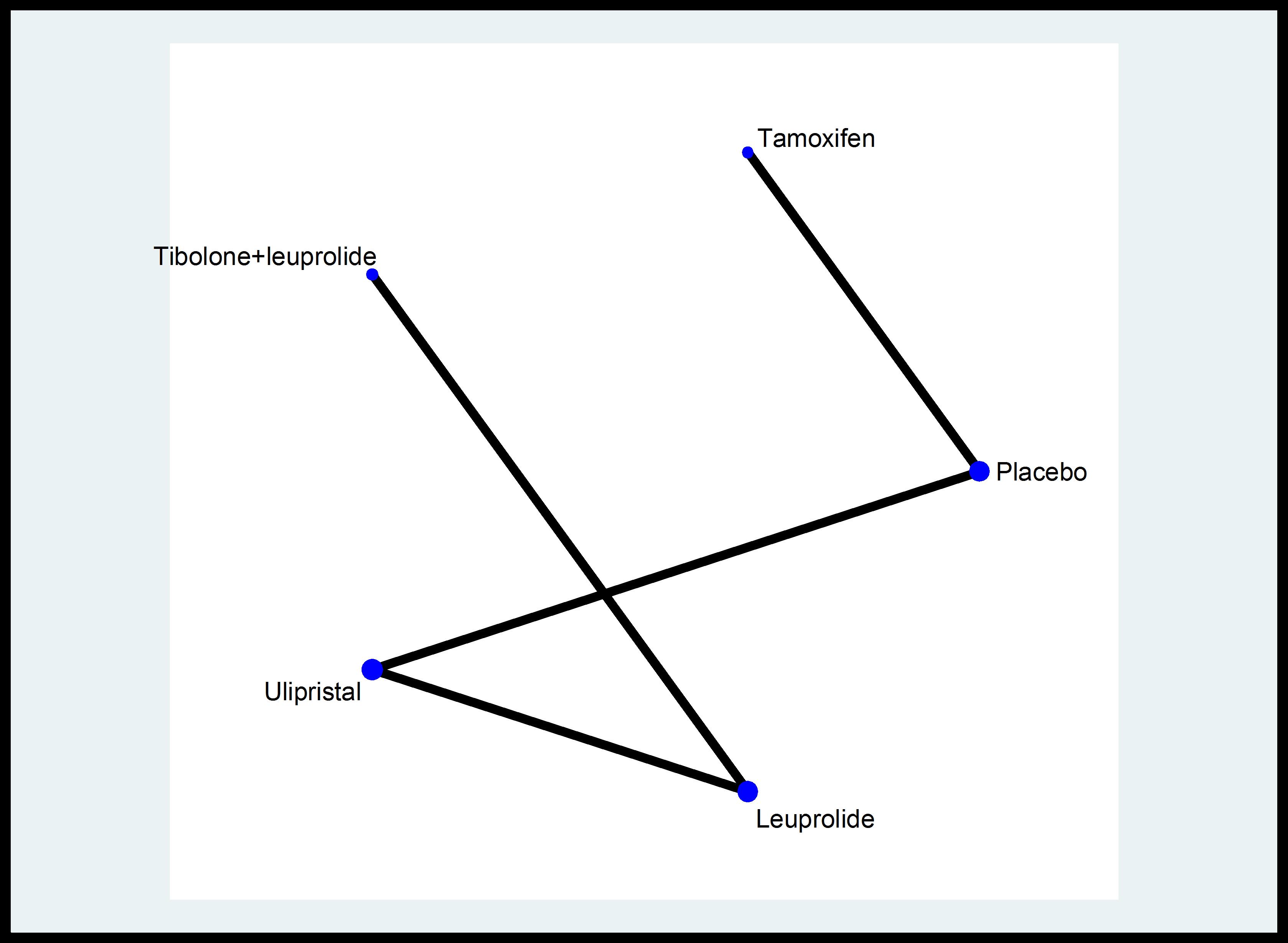

Supplement: S3 Fig — (JPG) [file pone.0149631.s006.jpg]

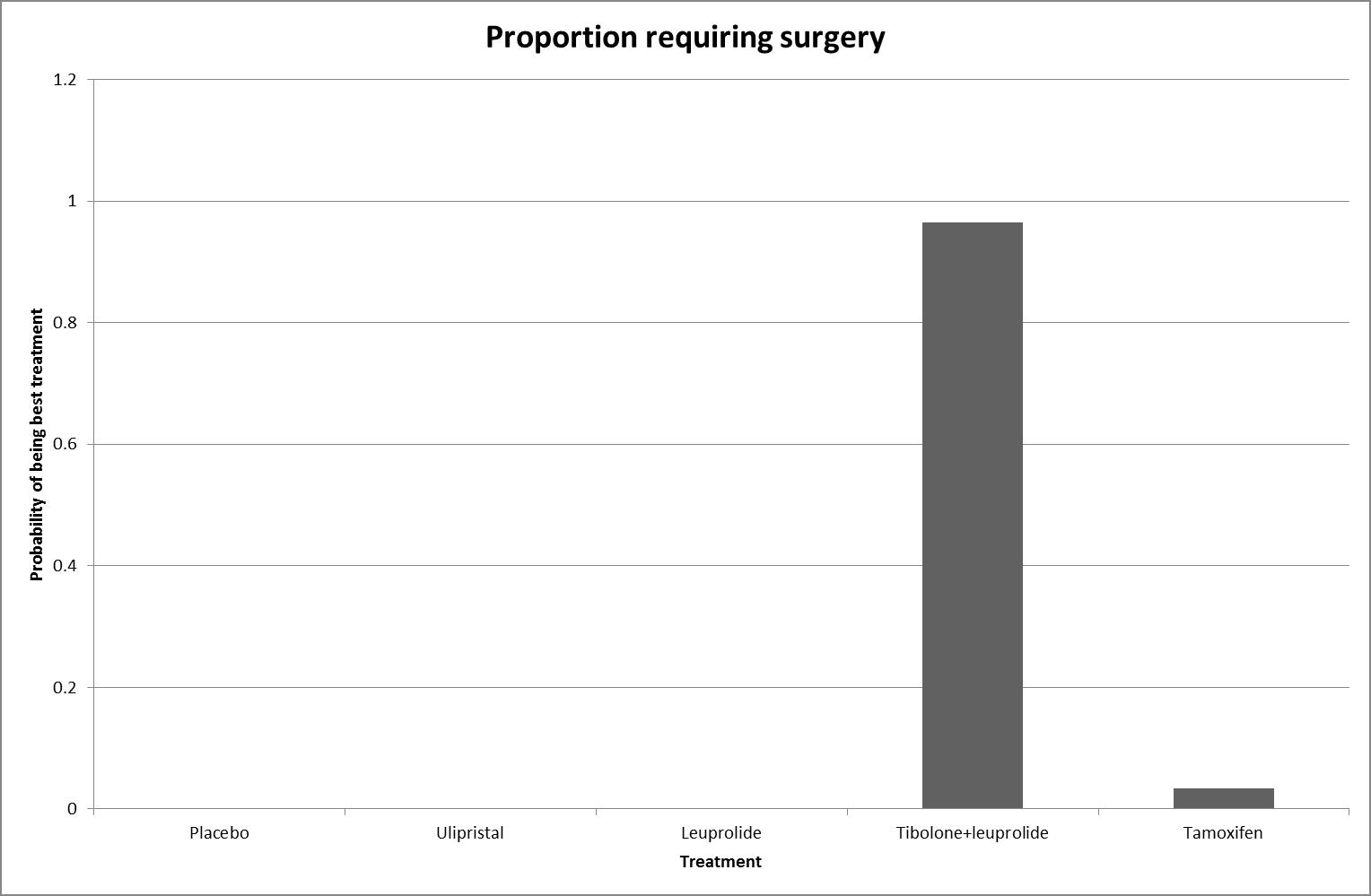

Supplement: S4 Fig — (JPG) [file pone.0149631.s007.jpg]

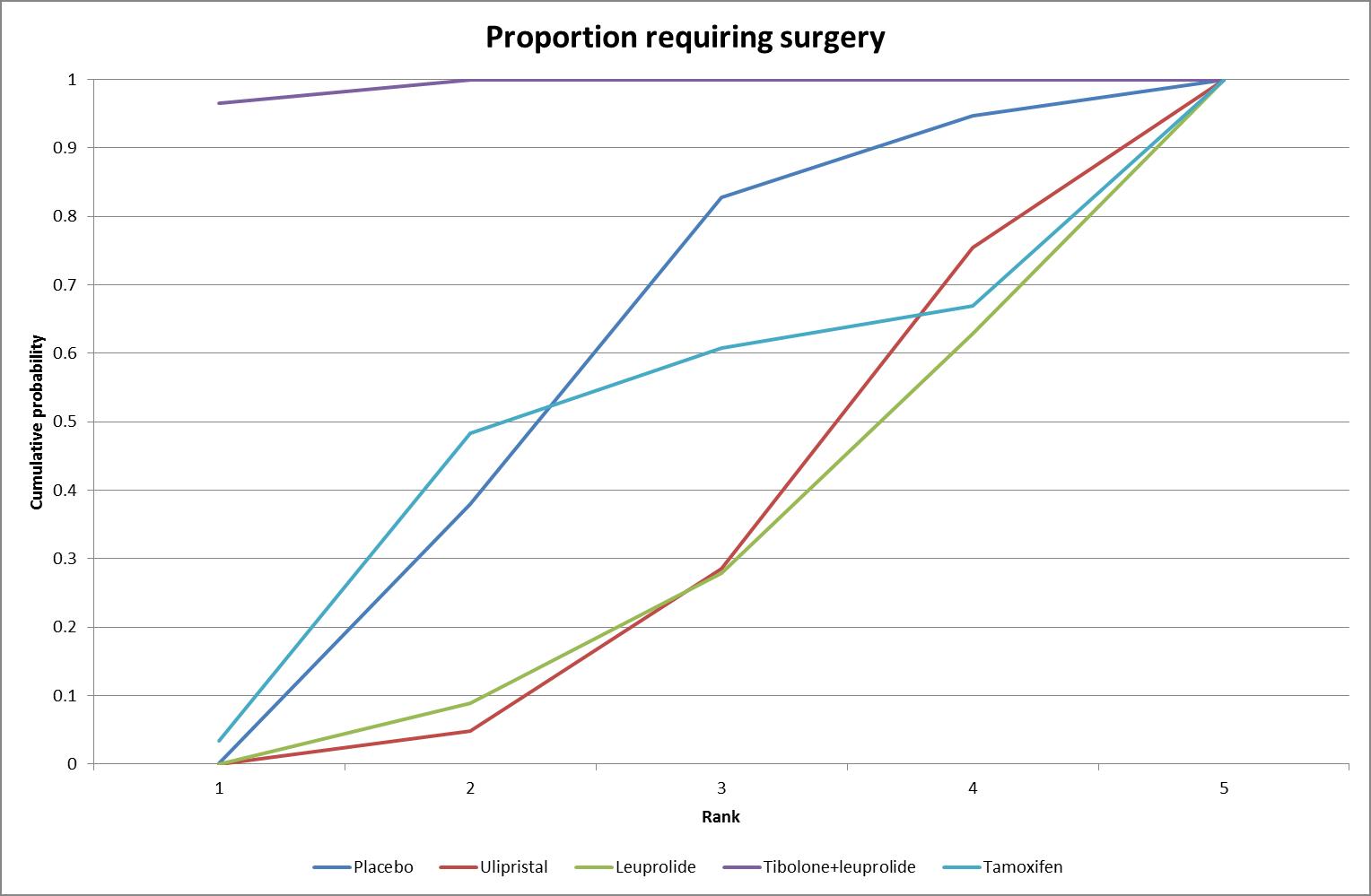

Supplement: S5 Fig — (JPG) [file pone.0149631.s008.jpg]

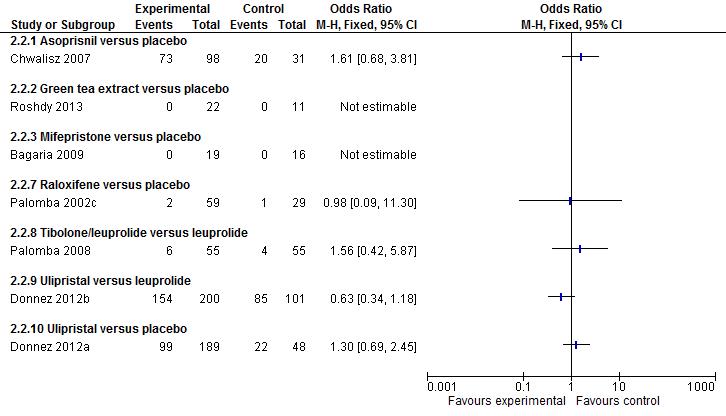

Supplement: S6 Fig — (JPG) [file pone.0149631.s009.jpg]

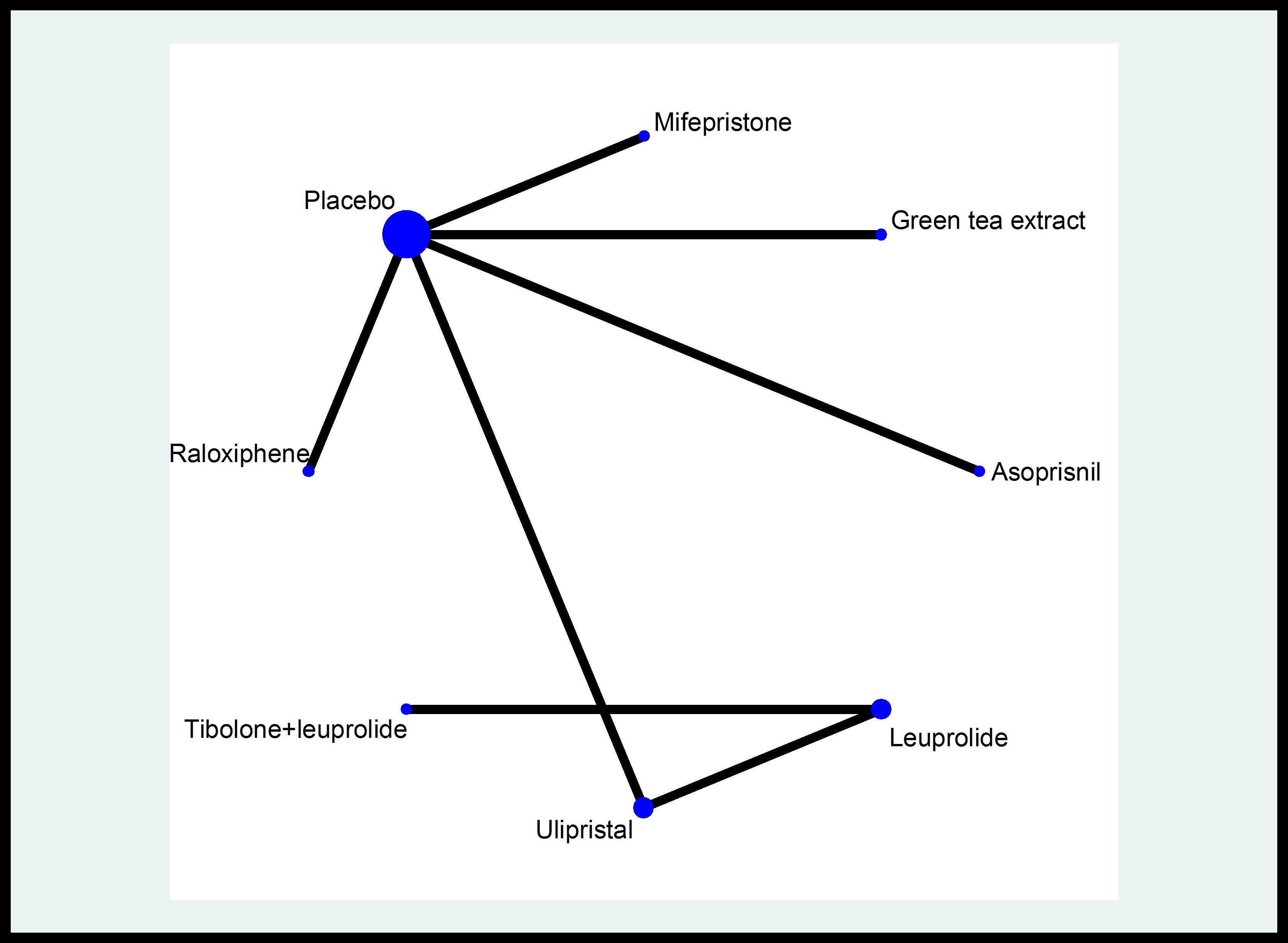

Supplement: S7 Fig — (JPG) [file pone.0149631.s010.jpg]

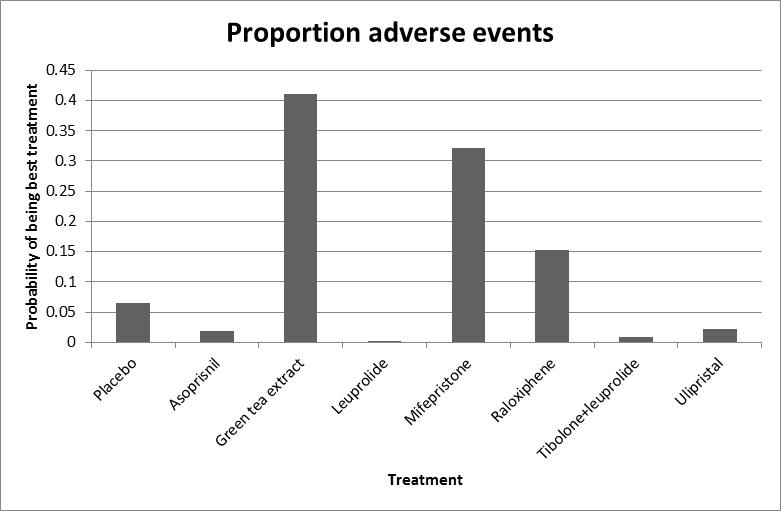

Supplement: S8 Fig — (JPG) [file pone.0149631.s011.jpg]

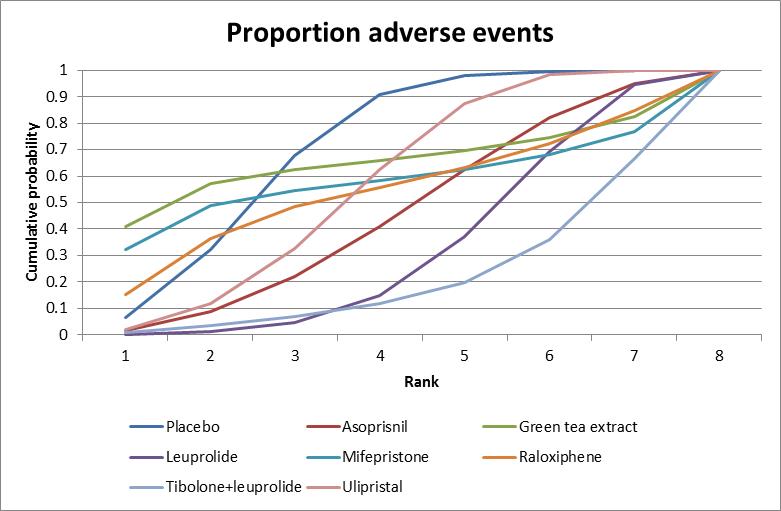

Supplement: S9 Fig — (JPG) [file pone.0149631.s012.jpg]

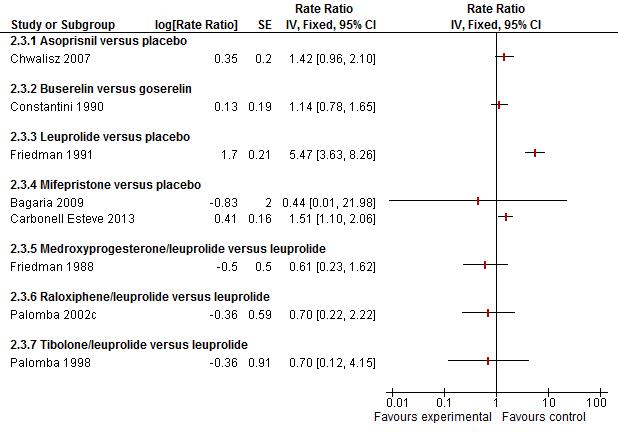

Supplement: S10 Fig — (JPG) [file pone.0149631.s013.jpg]

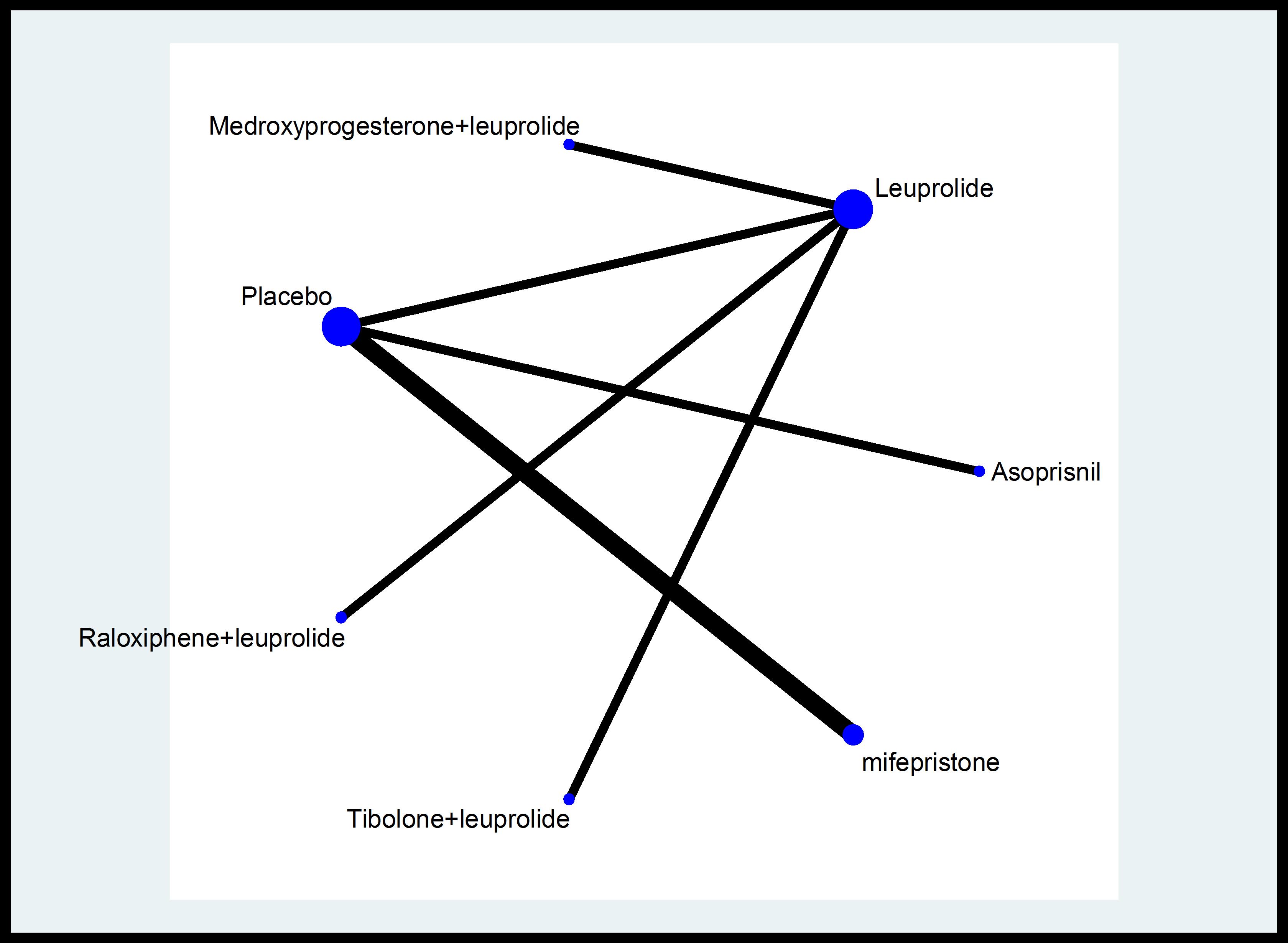

Supplement: S11 Fig — (JPG) [file pone.0149631.s014.jpg]

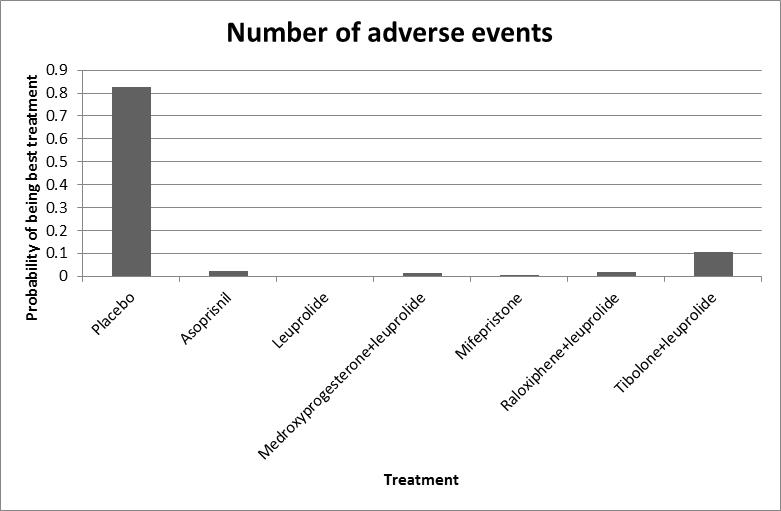

Supplement: S12 Fig — (JPG) [file pone.0149631.s015.jpg]

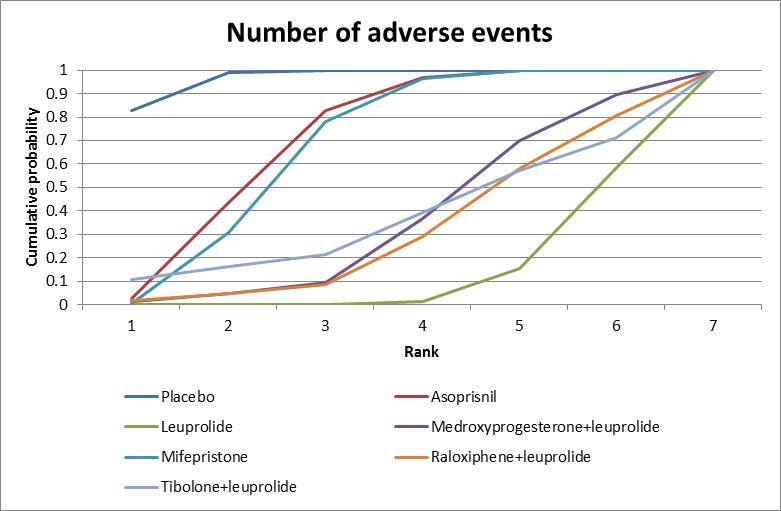

Supplement: S13 Fig — (JPG) [file pone.0149631.s016.jpg]

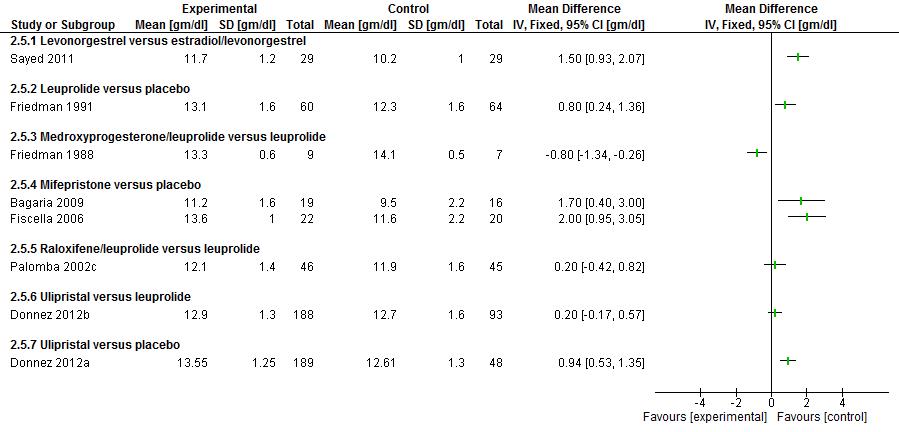

Supplement: S14 Fig — (JPG) [file pone.0149631.s017.jpg]

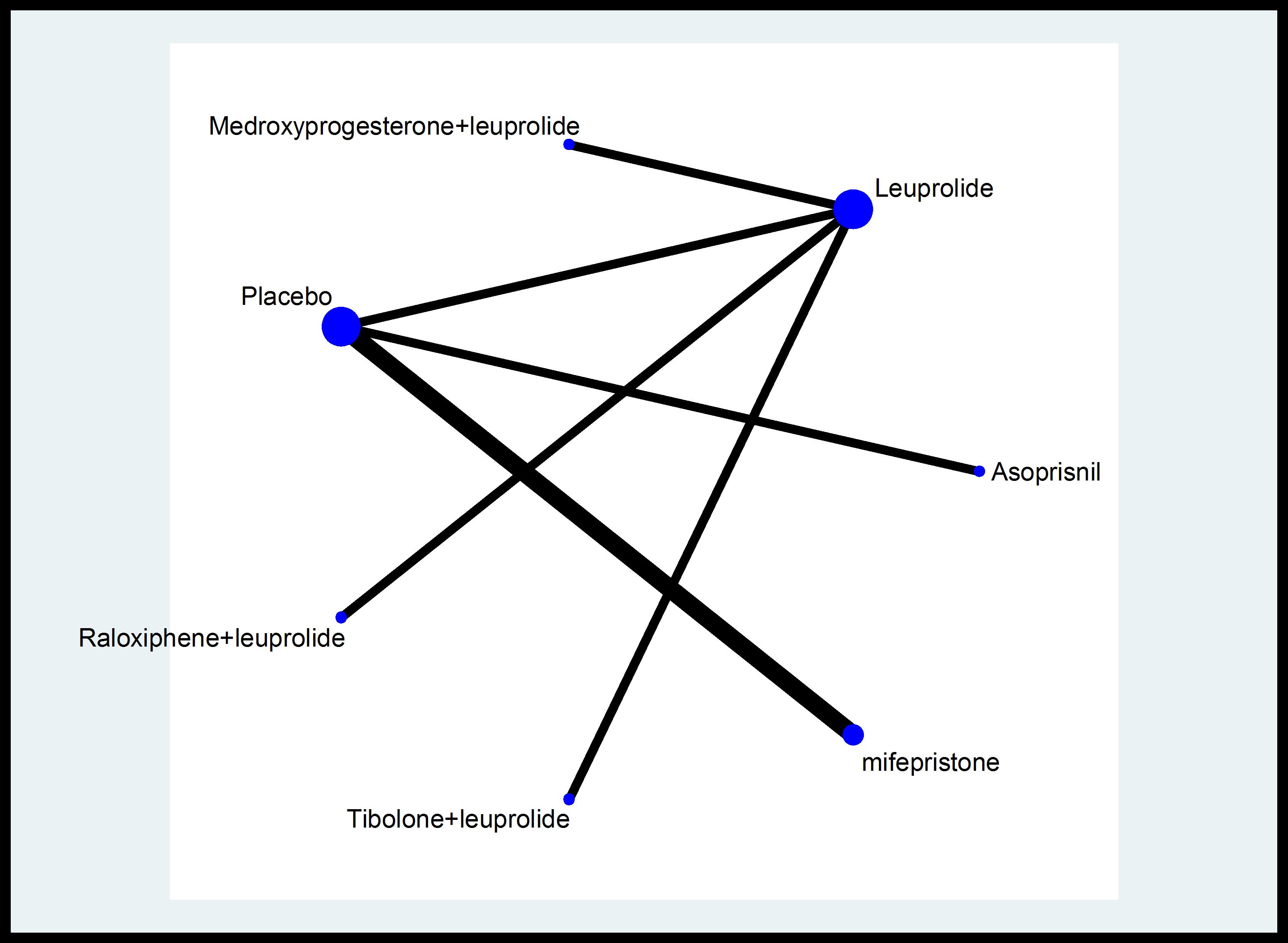

Supplement: S15 Fig — (JPG) [file pone.0149631.s018.jpg]

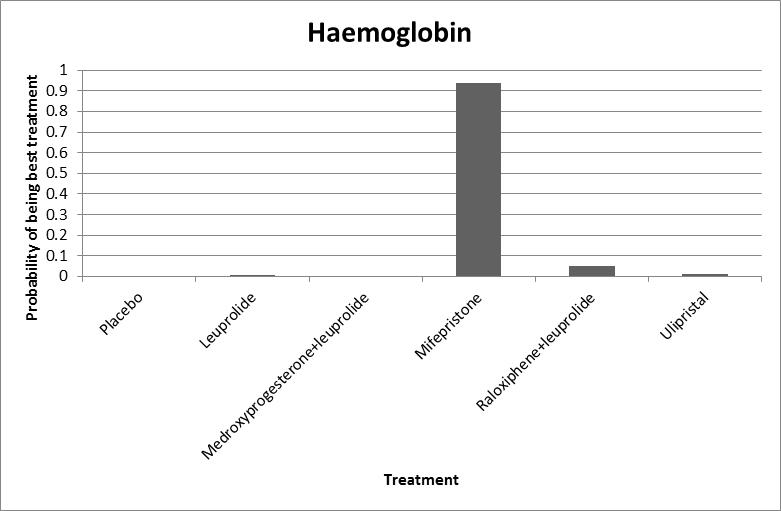

Supplement: S16 Fig — (JPG) [file pone.0149631.s019.jpg]

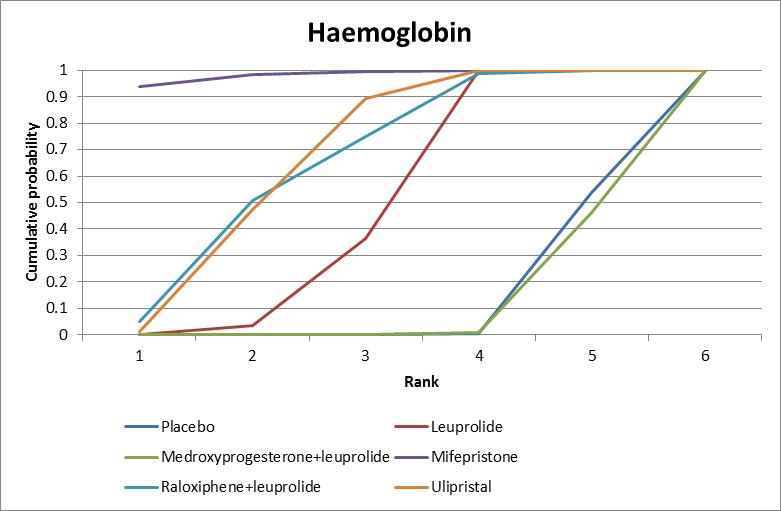

Supplement: S17 Fig — (JPG) [file pone.0149631.s020.jpg]

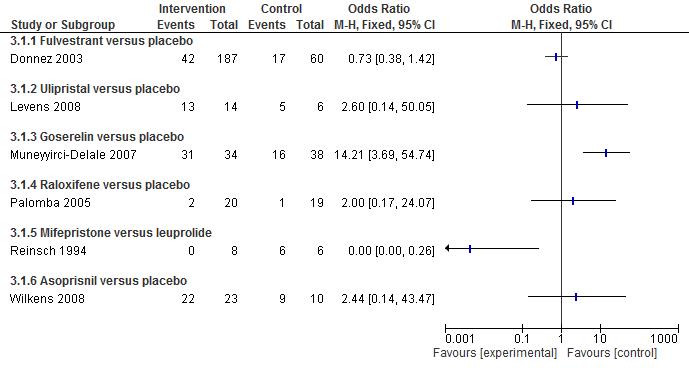

Supplement: S18 Fig — (JPG) [file pone.0149631.s021.jpg]

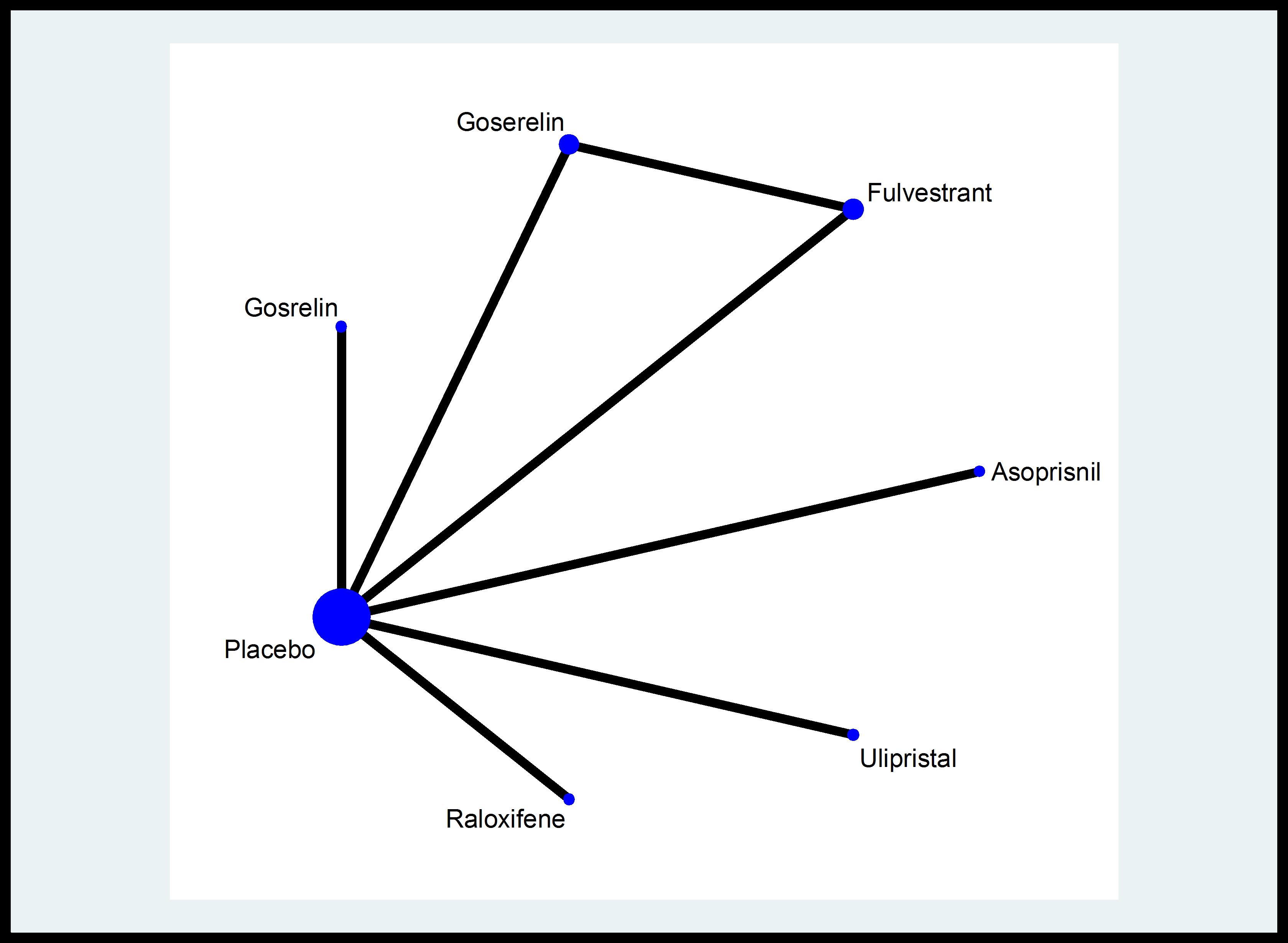

Supplement: S19 Fig — (JPG) [file pone.0149631.s022.jpg]

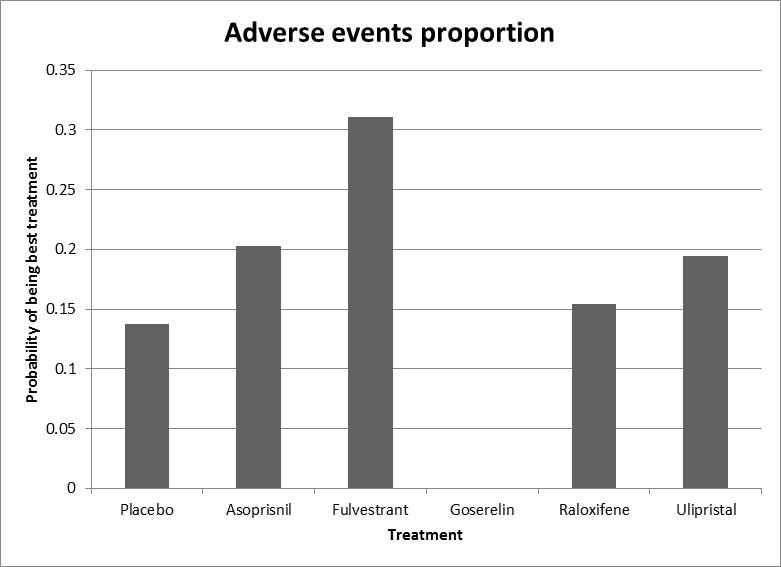

Supplement: S20 Fig — (JPG) [file pone.0149631.s023.jpg]

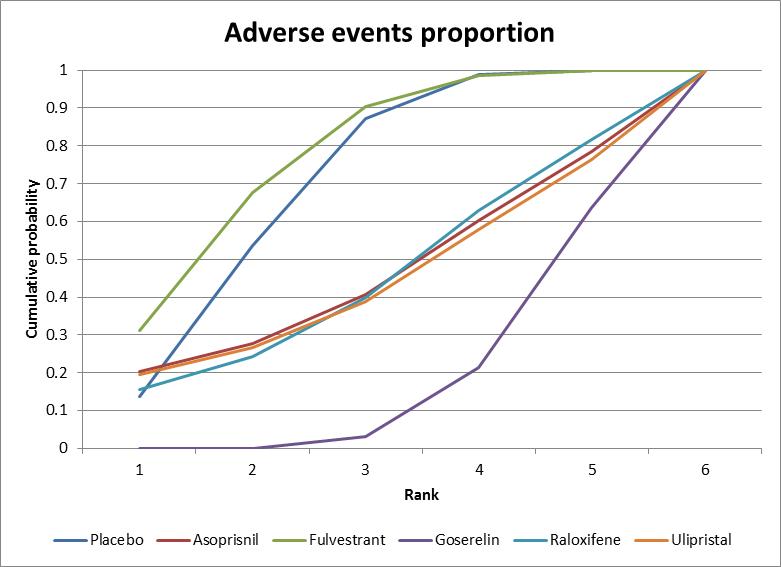

Supplement: S21 Fig — (JPG) [file pone.0149631.s024.jpg]

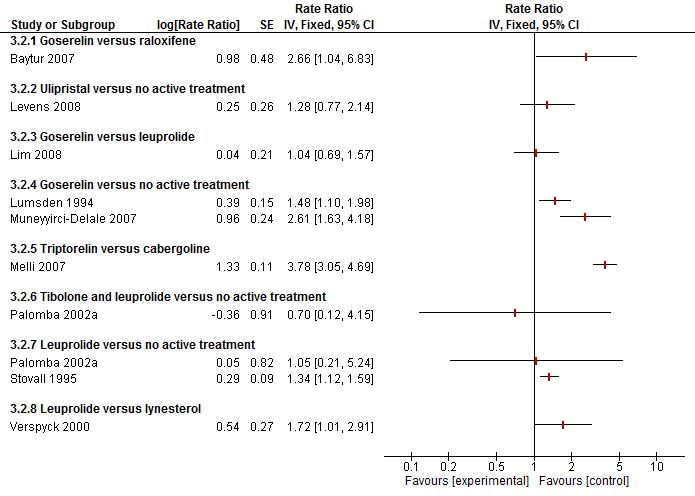

Supplement: S22 Fig — (JPG) [file pone.0149631.s025.jpg]

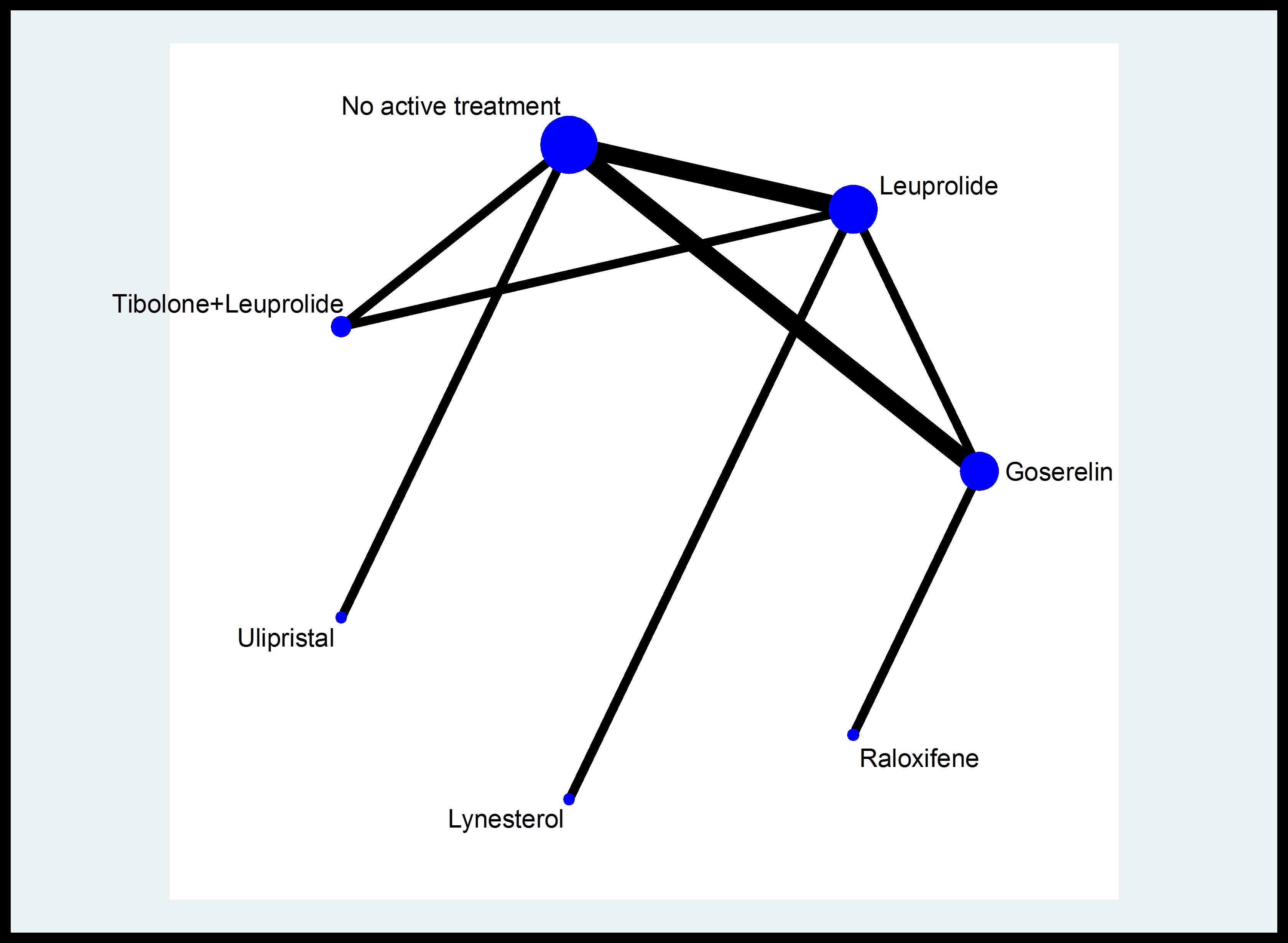

Supplement: S23 Fig — (JPG) [file pone.0149631.s026.jpg]

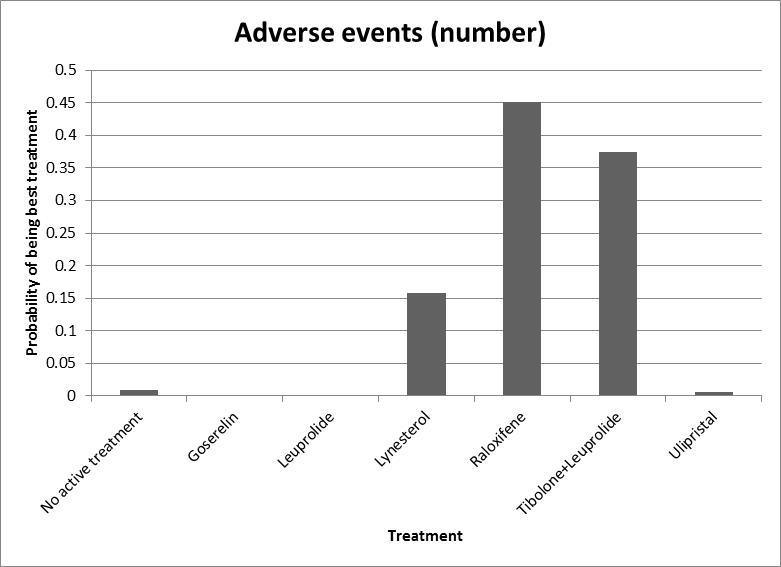

Supplement: S24 Fig — (JPG) [file pone.0149631.s027.jpg]

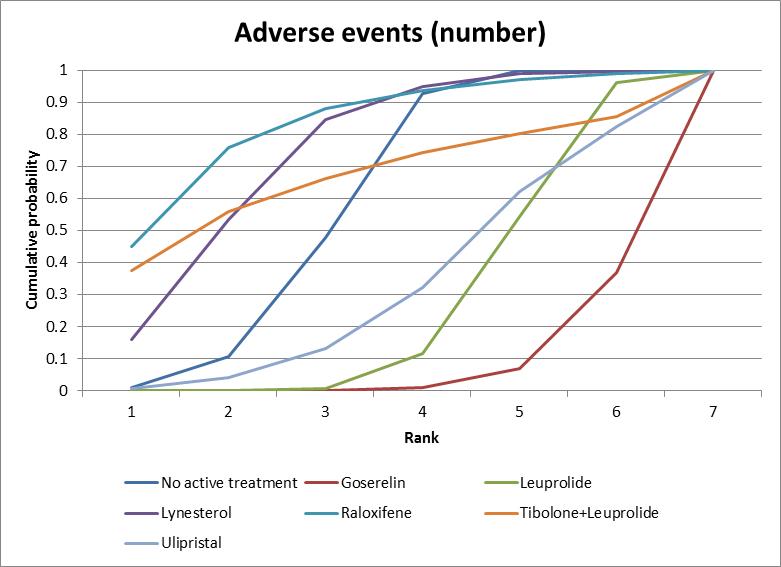

Supplement: S25 Fig — (JPG) [file pone.0149631.s028.jpg]

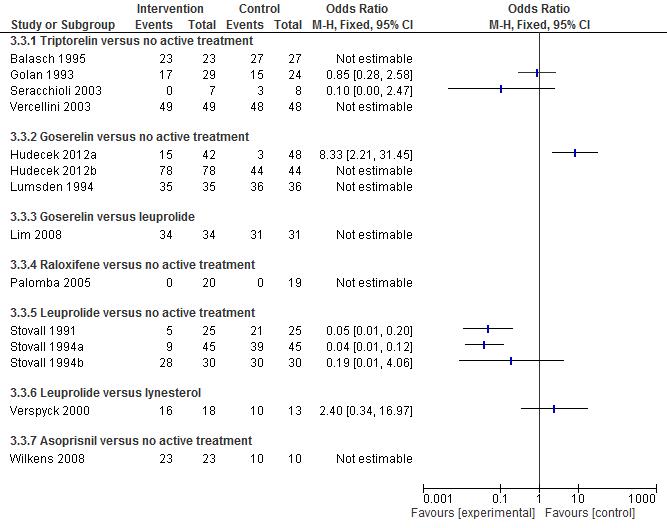

Supplement: S26 Fig — (JPG) [file pone.0149631.s029.jpg]

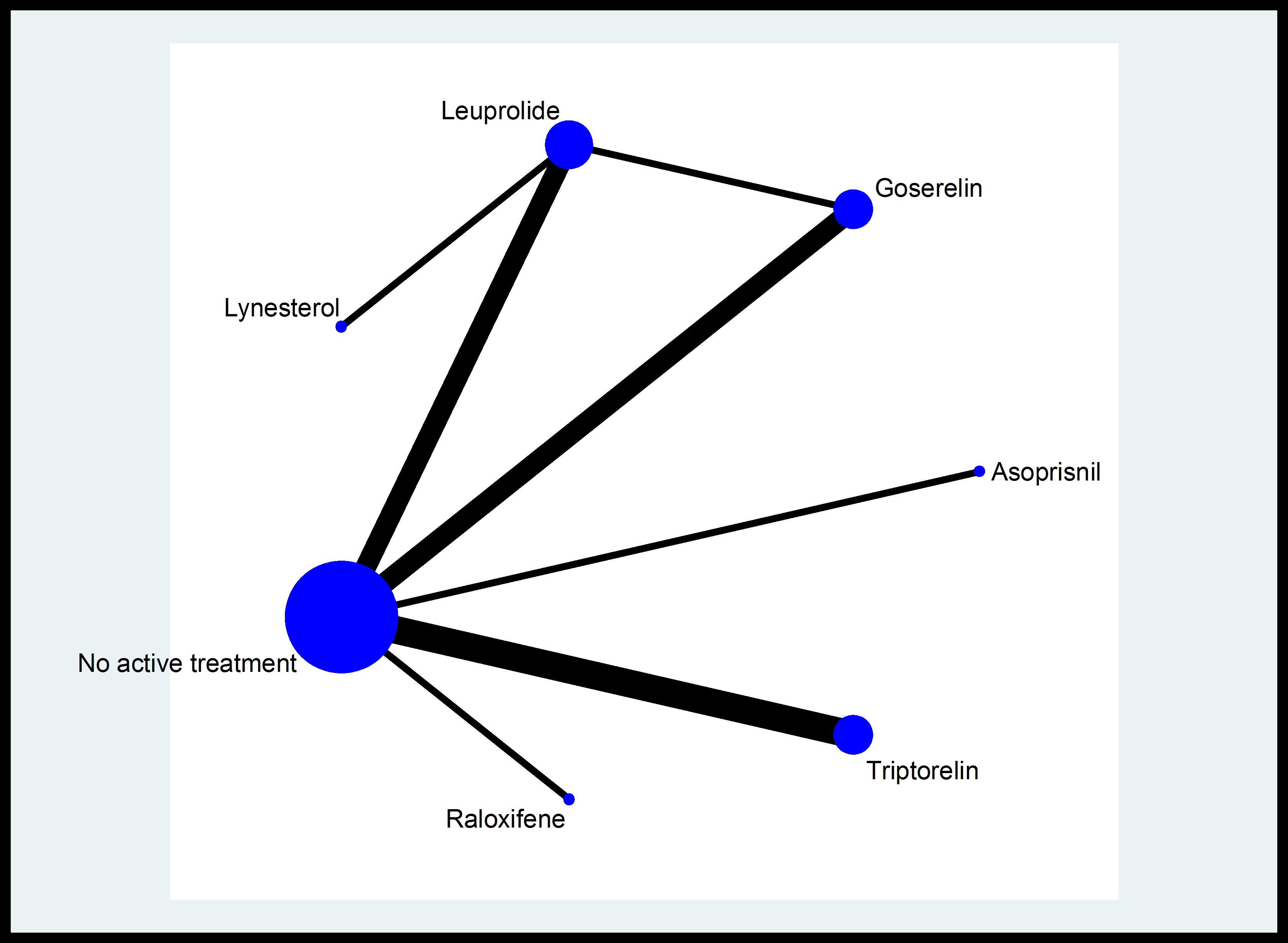

Supplement: S27 Fig — (JPG) [file pone.0149631.s030.jpg]

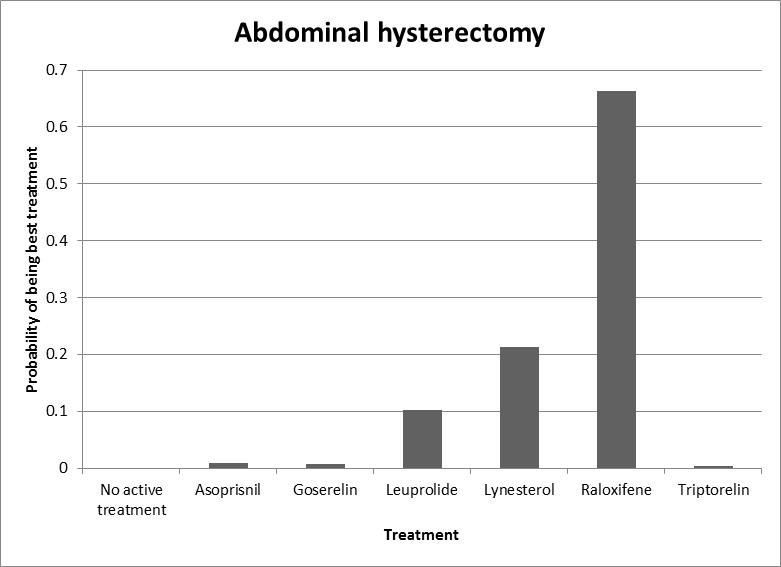

Supplement: S28 Fig — (JPG) [file pone.0149631.s031.jpg]

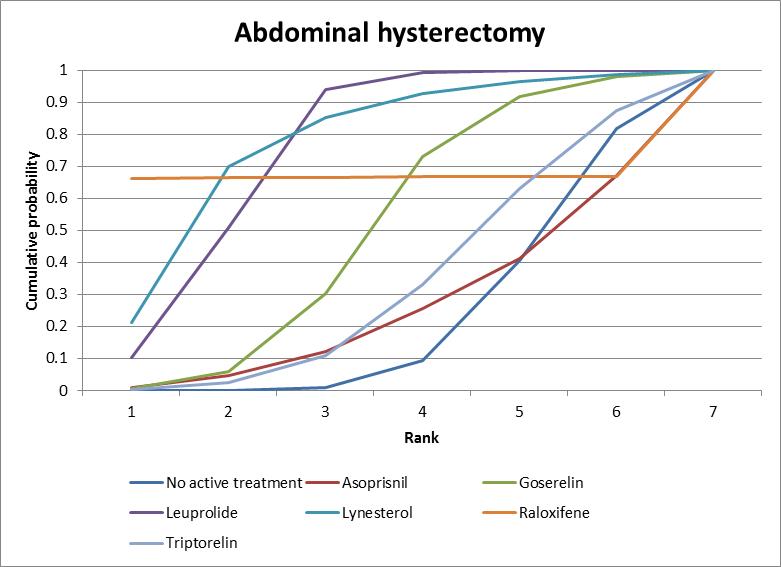

Supplement: S29 Fig — (JPG) [file pone.0149631.s032.jpg]

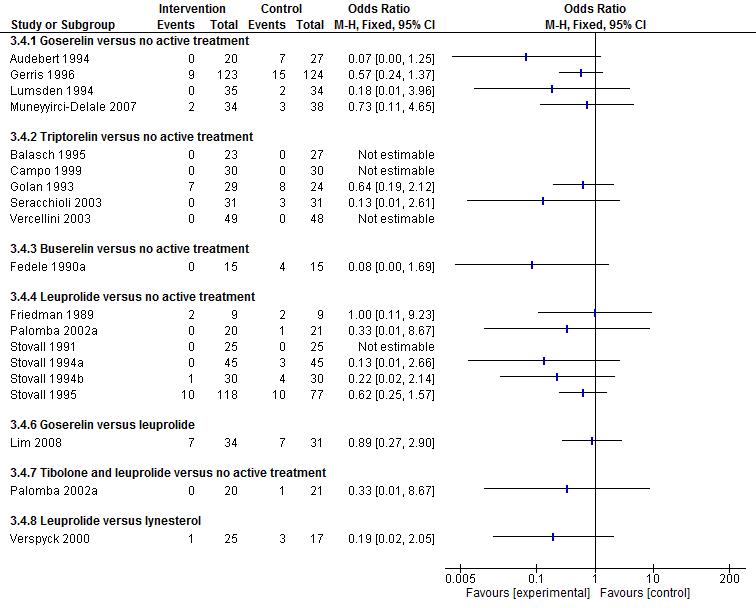

Supplement: S30 Fig — (JPG) [file pone.0149631.s033.jpg]

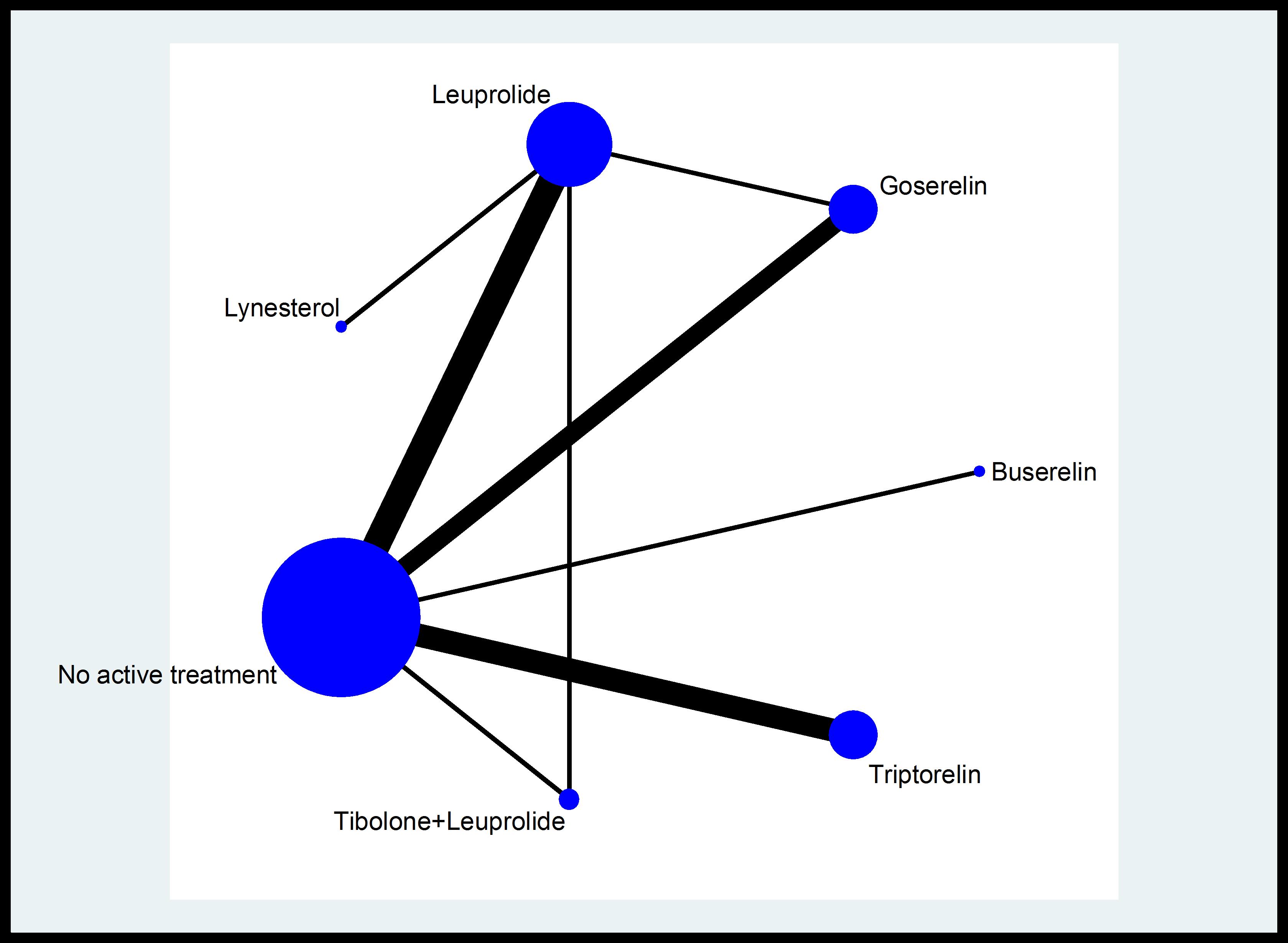

Supplement: S31 Fig — (JPG) [file pone.0149631.s034.jpg]

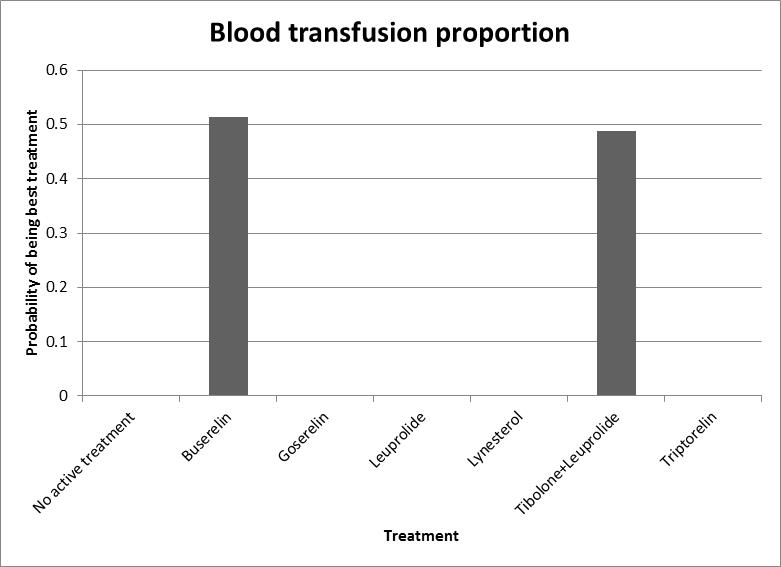

Supplement: S32 Fig — (JPG) [file pone.0149631.s035.jpg]

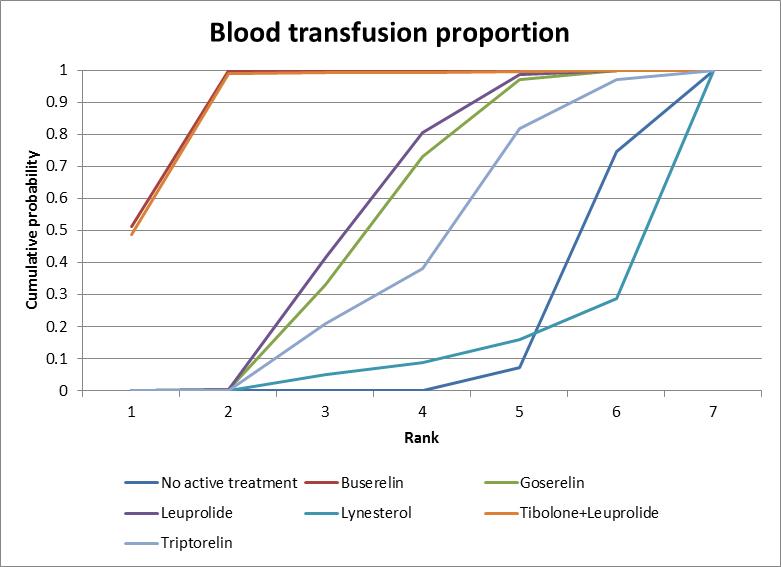

Supplement: S33 Fig — (JPG) [file pone.0149631.s036.jpg]

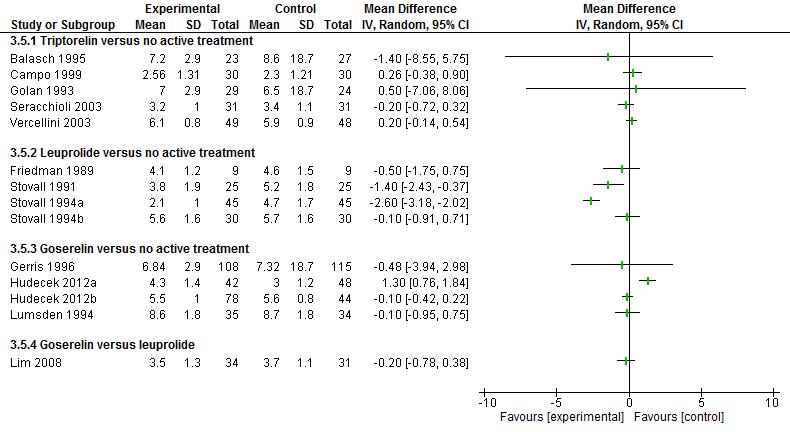

Supplement: S34 Fig — (JPG) [file pone.0149631.s037.jpg]

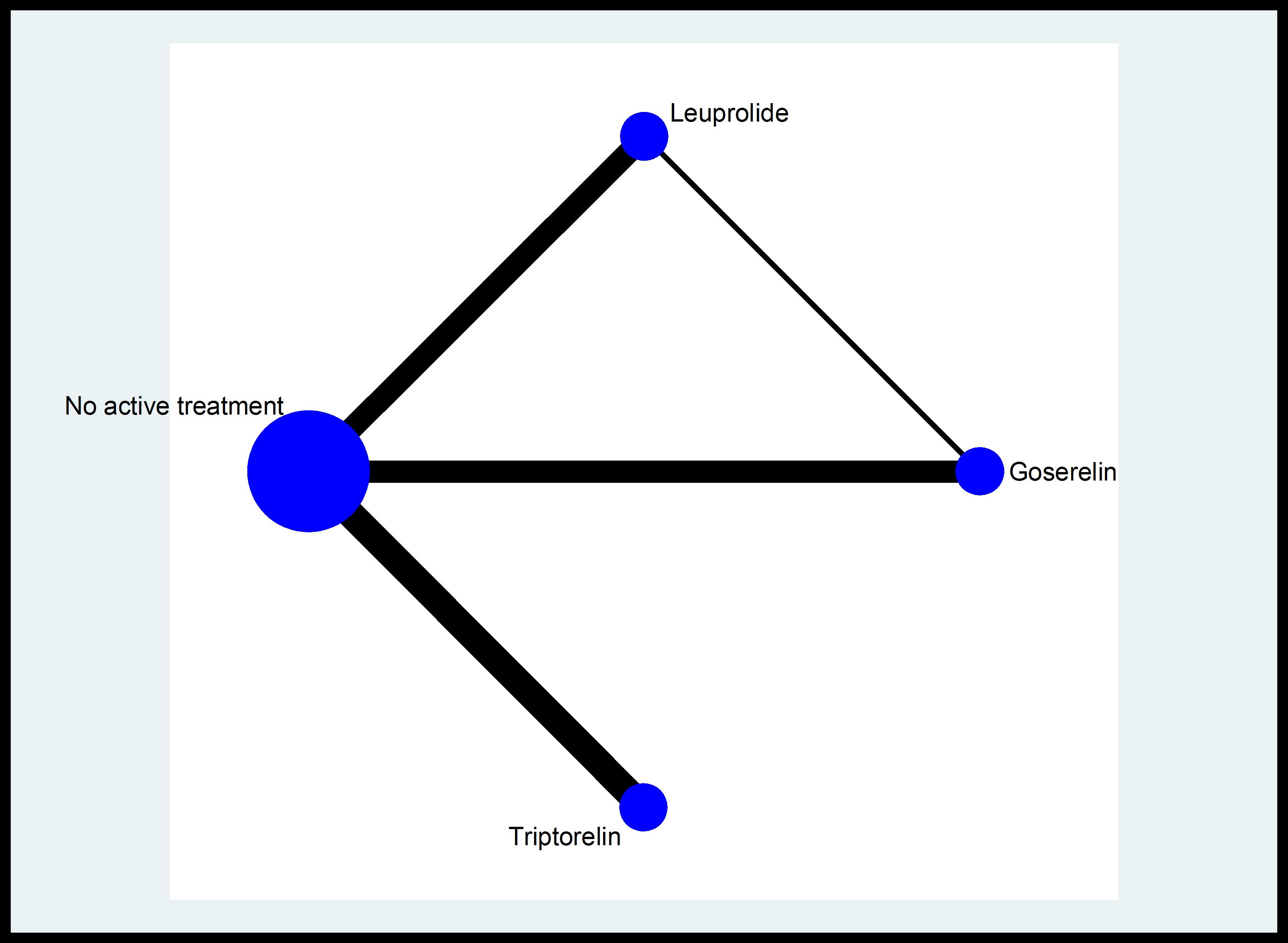

Supplement: S35 Fig — (JPG) [file pone.0149631.s038.jpg]

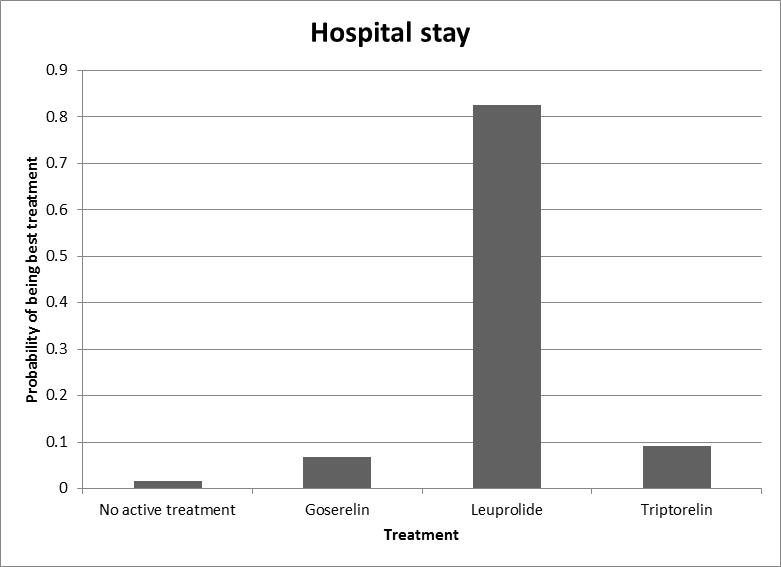

Supplement: S36 Fig — (JPG) [file pone.0149631.s039.jpg]

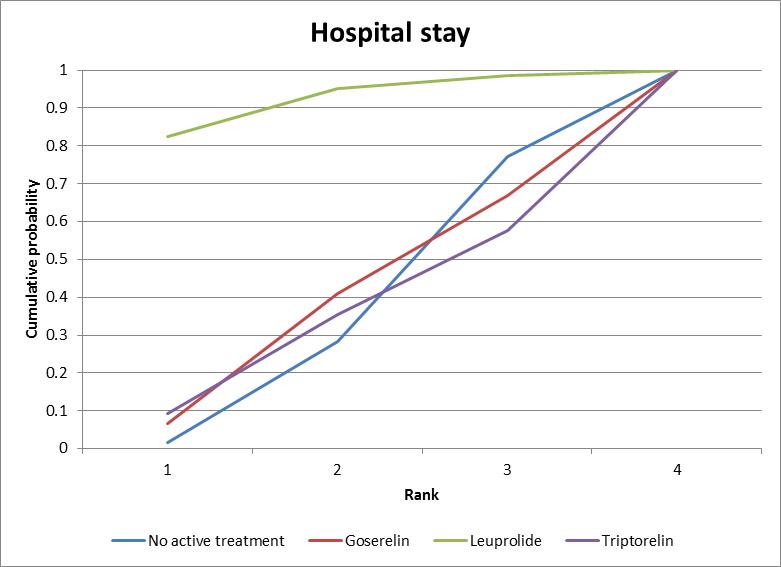

Supplement: S37 Fig — (JPG) [file pone.0149631.s040.jpg]

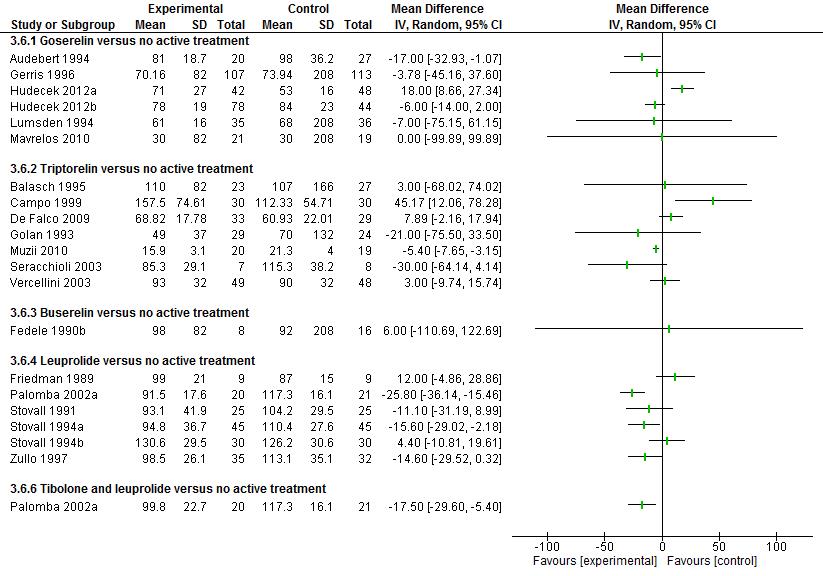

Supplement: S38 Fig — (JPG) [file pone.0149631.s041.jpg]

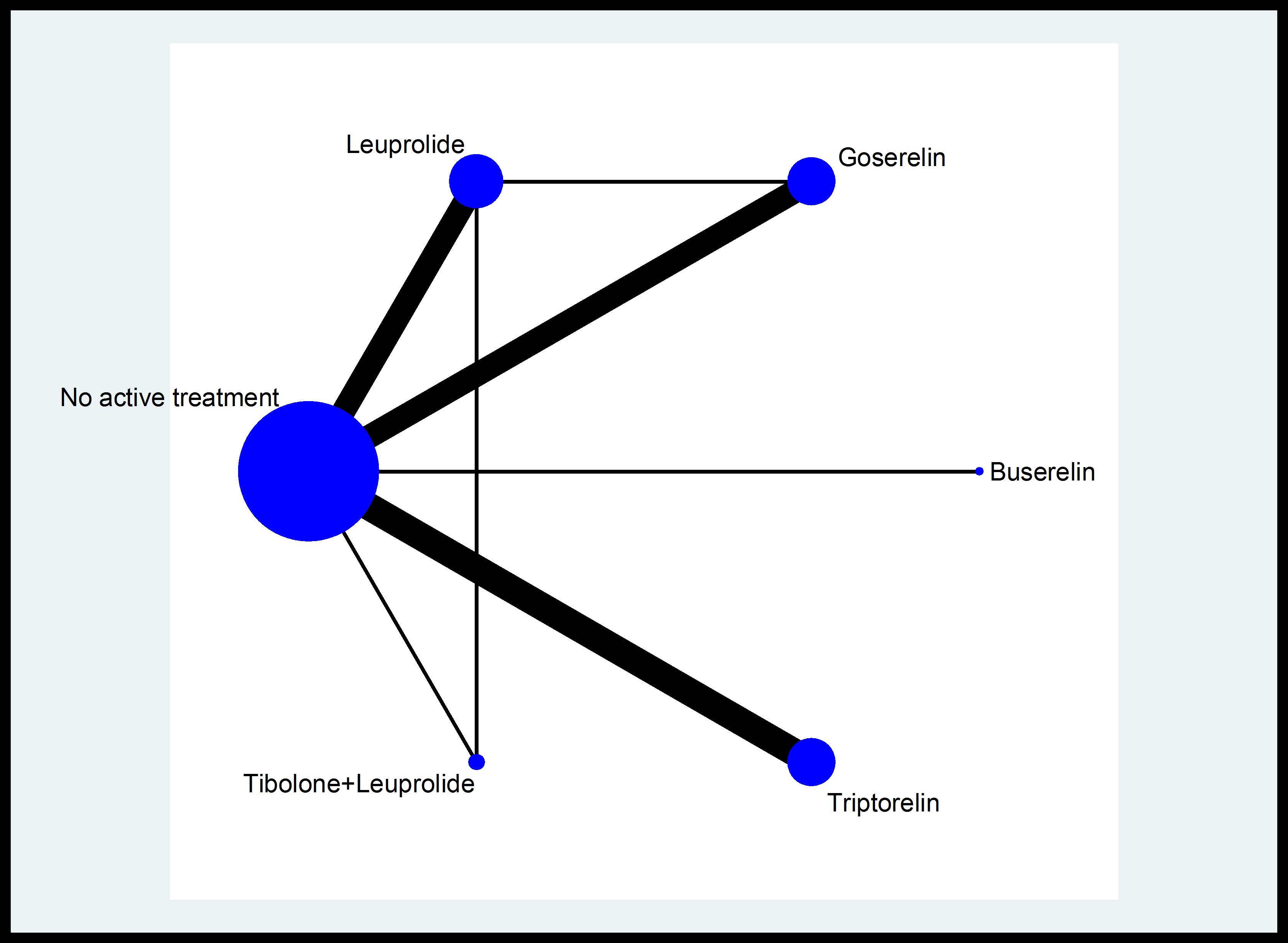

Supplement: S39 Fig — (JPG) [file pone.0149631.s042.jpg]

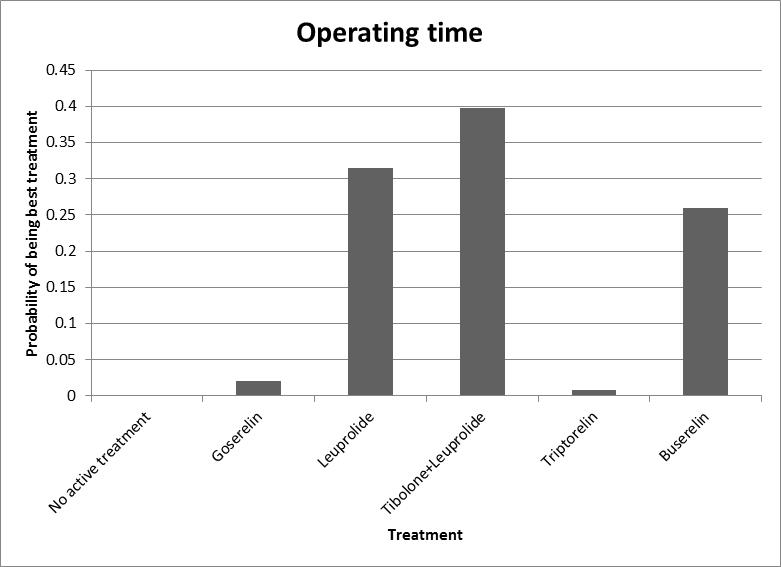

Supplement: S40 Fig — (JPG) [file pone.0149631.s043.jpg]

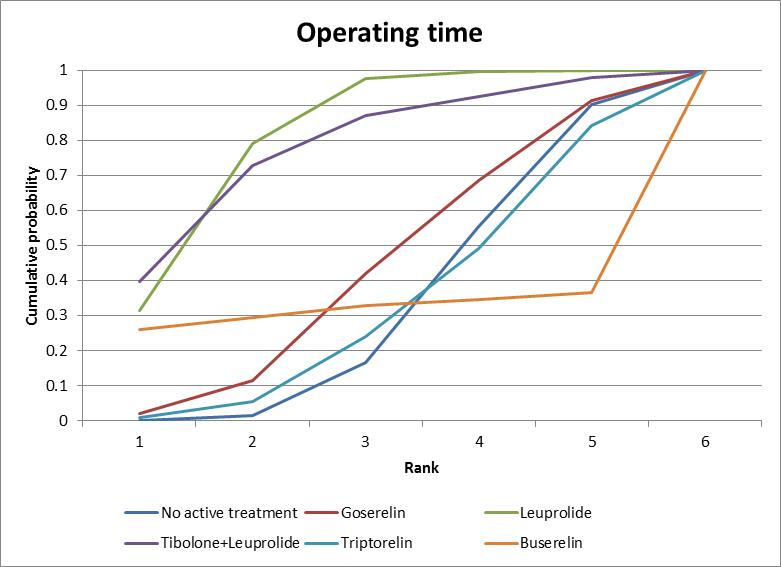

Supplement: S41 Fig — (JPG) [file pone.0149631.s044.jpg]

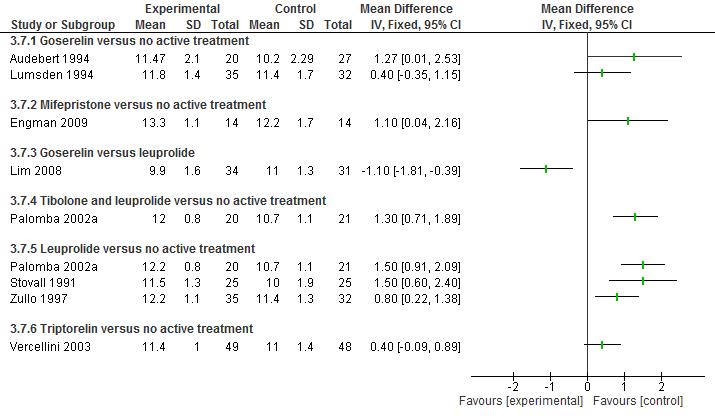

Supplement: S42 Fig — (JPG) [file pone.0149631.s045.jpg]

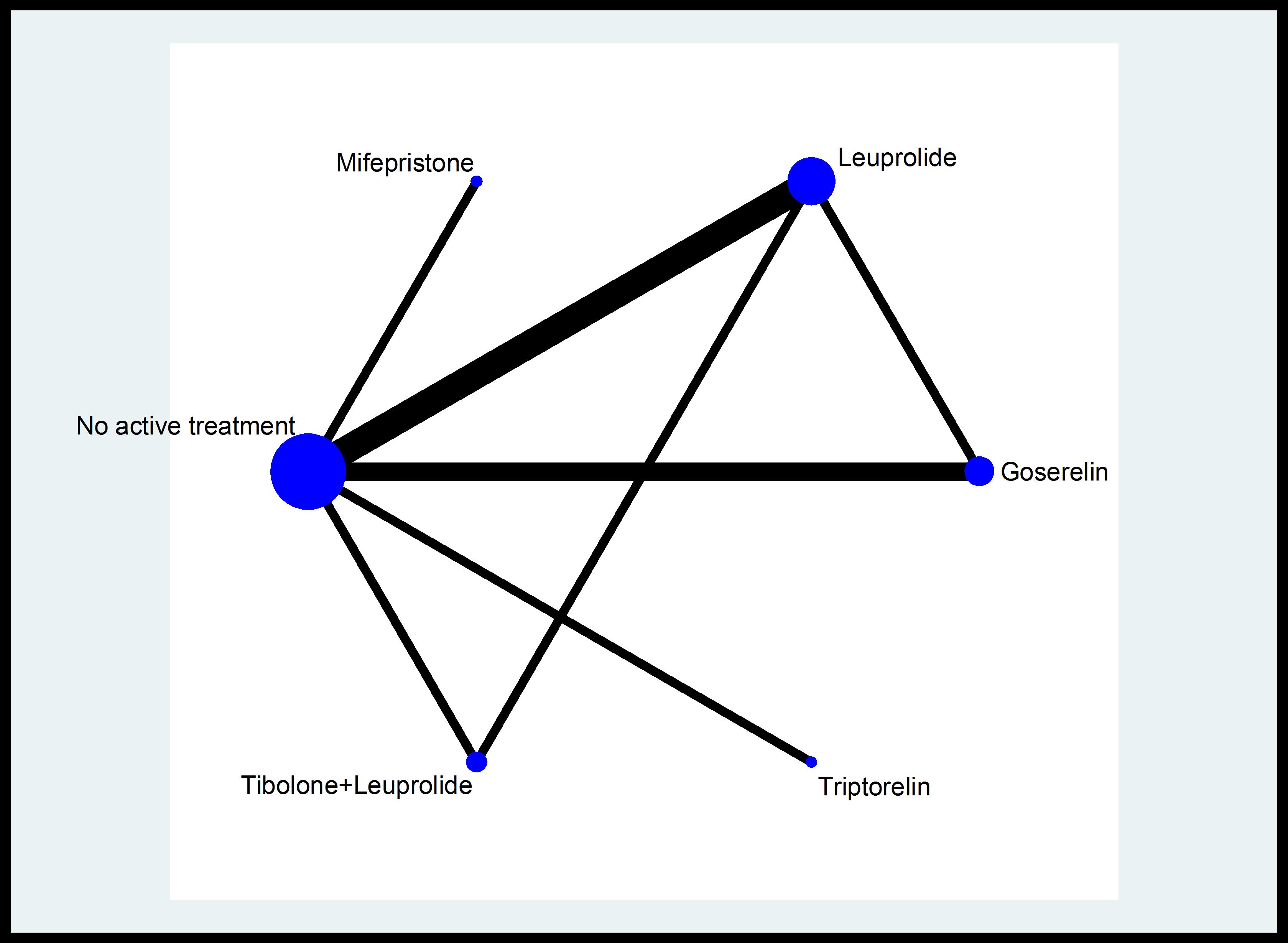

Supplement: S43 Fig — (JPG) [file pone.0149631.s046.jpg]

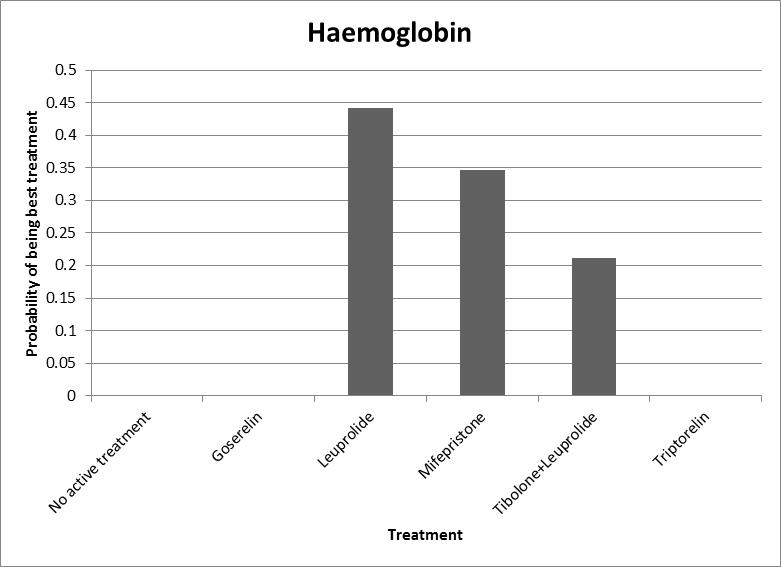

Supplement: S44 Fig — (JPG) [file pone.0149631.s047.jpg]

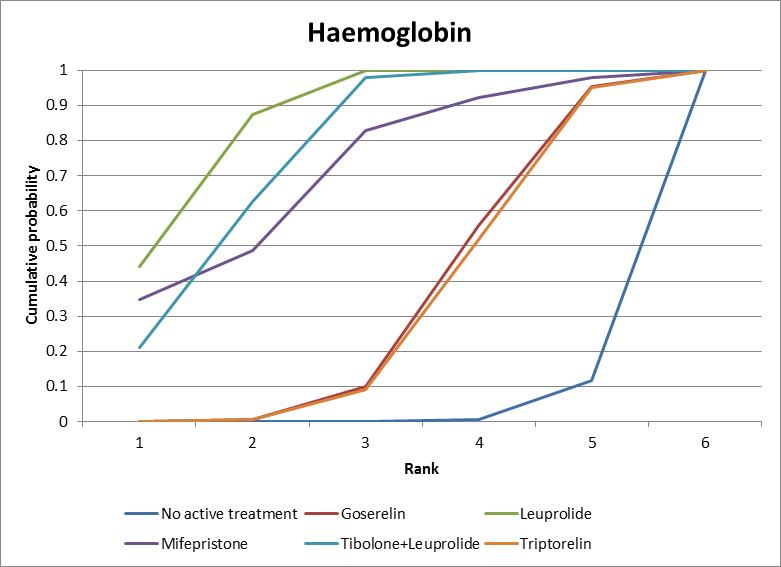

Supplement: S45 Fig — (JPG) [file pone.0149631.s048.jpg]
